# Assessment of the Concentration of 51 Elements in the Liver and in Various Parts of the Human Brain—Profiling of the Mineral Status

**DOI:** 10.3390/nu15122799

**Published:** 2023-06-19

**Authors:** Jacek Baj, Beata Kowalska, Wojciech Flieger, Elżbieta Radzikowska-Büchner, Alicja Forma, Marcin Czeczelewski, Paweł Kędzierawski, Kaja Karakuła, Michał Flieger, Dariusz Majerek, Grzegorz Teresiński, Ryszard Maciejewski, Jolanta Flieger

**Affiliations:** 1 Department of Anatomy, Medical University of Lublin, 20-090 Lublin, Poland; wwoj24@wp.pl (W.F.); aforma@onet.pl (A.F.); ryszard.maciejewski@umlub.pl (R.M.); 2 Department of Water Supply and Wastewater Disposal, Lublin University of Technology, 20-618 Lublin, Poland; b.kowalska@pollub.pl; 3 Department of Plastic, Reconstructive and Maxillary Surgery, CSK MSWiA, 02-507 Warszawa, Poland; elzbieta.radzikowska@gmail.com; 4 Department of Forensic Medicine, Medical University of Lublin, 20-090 Lublin, Poland; marcin.czeczelewski@gmail.com (M.C.); pawelkedzierawski1@gmail.com (P.K.); michalflieeeger@gmail.com (M.F.); grzegorz.teresinski@umlub.pl (G.T.); 5 I Department of Psychiatry, Psychotherapy, and Early Intervention, Medical University of Lublin, 20-439 Lublin, Poland; kaja.karakula@gmail.com; 6 Department of Applied Mathematics, University of Technology, 20-618 Lublin, Poland; d.majerek@pollub.pl; 7 Department of Analytical Chemistry, Medical University of Lublin, 20-093 Lublin, Poland

**Keywords:** multi-elemental analysis, trace elements, micronutrient, mineral status, human tissues, brain, liver, ICP-MS

## Abstract

The anthropogenic environment and diet introduce many metals into the human body, both essential and toxic. Absorption leads to systemic exposure and accumulation in body fluids and tissues. Both excess and deficiency of trace elements are health hazards. The primary aim of the present study was to evaluate the concentration of 51 elements in liver samples and 11 selected brain regions obtained at post-mortem examination from a population of adults living in south-eastern Poland (*n* = 15). A total of 180 analyses were performed by inductively coupled plasma mass spectrometry in two independent replicates. The collected data show very high individual variability in the content of the investigated elements. Macroelements such as sodium, magnesium, phosphorus, potassium, calcium, iron, and zinc occurred in the highest concentrations and with the greatest statistically significant variations. Although the elemental content of the brain and liver differed significantly, the strongest positive correlation between liver and polus frontalis was observed for the essential element selenium (0.9338) and the strongest negative one for manganese (−0.4316) and lanthanum (−0.5110). The brain areas studied have different requirements for phosphorus, manganese, iron, and molybdenum. In addition, males had a significantly (*p* < 0.05) higher brain content of lanthanides and actinides than females. The results of this study show that the inhabitants of south-eastern Poland are exposed to a fairly uniform accumulation of aluminum and vanadium in the brain, which have the highest affinity to the thalamus dorsalis. This result proves that there is environmental exposure to these elements.

## 1. Introduction

Essential elements play a fundamental role in the physiological processes that determine growth, health, and reproductive capacity [1]. The human body does not synthesize bioelements, which must be supplied through the diet [2]. Any disruption in the supply of essential elements leads to dyshomeostasis and reduces the ability to adapt to adverse environmental conditions, increasing susceptibility to diseases, including COVID-19 [3,4,5]. Depending on the body needs, all elements are classified as macroelements (oxygen—O, carbon—C, hydrogen—H, nitrogen—N, calcium—Ca, phosphorus—P, potassium—K, sodium—Na, sulphur—S, chlorine—Cl, magnesium—Mg), trace elements (iron—Fe, zinc—Zn, fluorine—F, strontium—Sr, molybdenum—Mo, copper—Cu, bromine—Br, silicon—Si, cesium—Cs, iodine—I, manganese—Mn, aluminum—Al, lead—Pb, cadmium—Cd, boron—B, rubidium—Rb) and the ultra-trace elements (selenium—Se, cobalt—Co, vanadium—V, chromium—Cr, arsenic—As, nickel—Ni, lithium—Li, barium—Ba, titanium—Ti, silver—Ag, tin—Sn, beryllium—Be, gallium—Ga, germanium—Ge, mercury—Hg, scandium—Sc, zirconium—Zr, bismuth—Bi, antimony—Sb, uranium—U, thorium—Th, rhodium—Rh) [6]. It is interesting to note that the total content of structural and functional elements in the body is proportional to their presence in the Earth’s crust. For example, Ca, K, Na, Mg, Fe, and Zn are the most abundant both in the Earth’s crust and in the composition of living organisms (WebElements.com, (accessed on 30 April 2023)) [7].

The role of biometals in biochemical processes in the body is very broad and includes cell signaling, maintenance of membrane potential, immune defense, cellular energy supply, and metabolism of amino acids, lipids, proteins, and carbohydrates [8]. Biometals are associated with 25–30% of proteins involved in the structure and function of physiological processes [9], and 4–10% of all proteins are Zn-metalloproteins. In turn, Ca, Mg, Mn, Zn, and Co are co-factors of metalloproteins involved in sugar metabolism and glycosylation [9]. Some elements may have similar functions; others may have synergistic and antagonistic effects [10,11]. For example, the altered status of Fe in the body affects the homeostasis of Mn in such a way that an excess of Fe enhances the neurotoxicity caused by Mn [12]. The quantitative aspect of inter-elemental interactions is a research trend that has been developing for years. As a result, it has been possible to identify deficiencies of essential trace elements in different populations. One example is China, where Keshan heart disease is caused by Se deficiency [13]. Endemic deficiencies or excesses of certain elements are known in some parts of the world, e.g., I deficiency in Asia, Africa, and Australia [14], excessive exposure to F in the Himalayan region of India, Pakistan, and Bangladesh, etc. [15,16,17].

Some elements are toxic at very low concentrations, e.g., Pb, Cd, Hg, As, Mo, Be, Ga, Sb, radon (Rn), Bi, and polonium (Po). It has been documented that an increase in environmental Pb is directly proportional to a decrease in IQ in children [18]. The adverse effects of overexposure to metals and metalloids such as Al, Sb, As, Ba, Be, Bi, B, Cd, Cr, Co, Cu, Ga, indium (In), Ge, gold (Au), Fe, Pb, Li, Mn, Hg, Mo, Ni, niobium (Nb), tantalum (Ta), platinum (Pt), palladium (Pd), iridium (Ir), Rh, osmium (Os), ruthenium (Ru), yttrium (Y), lanthanum (La), Se, Ag, tellurium (Te), thallium (Tl), Sn, Ti, tungsten (W), V, Zn, Zr and hafnium (Hf) are well known in occupational medicine. Bioelement analysis from the highly industrialized region of Upper Silesia in Poland allows the identification of very high levels of Al, around 8.28 μg/g, in human livers [19]. On the other hand, liver biopsy samples from the Hungarian population show Ni accumulation [20]. Biomonitoring of trace elements in the blood and urine of occupationally exposed workers by sector showed elevated exposure risks for Be and also for Al, Cr, and Mn [21]. Multi-element profiles in autopsied organs (brain, heart, kidney, liver, lung, muscle, pancreas, spleen, and thyroid) showed that the trace elements Br, Cl, Co, Cu, Fe, K, Mn, Na, Rb, Se, and Zn were not affected by external environmental factors or racial differences [22]. However, very high levels of Cd in the kidneys and liver of healthy Japanese appear to be of concern. A multicomponent hair profile has been developed for people with depression [7]. The test showed the presence of traces of Au and Pb, elevated levels of Mg and Ca, suggesting disturbances in bone metabolism, deficiencies of I and Zn, suggesting reduced metabolic activity, and low levels of Co, suggesting a deficiency of cyanocobalamin, which is common in the elderly and in vegetarians. It should be emphasized that simultaneous exposure to several toxic elements would result in a more severe intoxication than if they were administered separately.

To understand the role of individual metals at the cellular level and then at higher levels of the organ and the whole organism, it is necessary to quantify and visualize the metal content at a specific location. Metallomics studies offer many advantages, among others: they allow the construction of multi-element profiles of tissues and organs and their relationship to different disease entities, reflect different environmental conditions, exposure to pollutants, and dietary influences, and allow the identification of different combinatorial relationships between elements.

Several instrumental techniques can be used for the elemental analysis of biomedical samples [7,22,23,24,25]. Scanning electron microscopy (SEM) with energy dispersive X-ray analysis (EDX) or Raman spectroscopy and laser ablation inductively coupled mass spectrometry (LA-ICP-MS) can be used for direct imaging of elements in biological samples [26]. Qualitative and quantitative analysis of trace and ultratrace elements can be performed using instrumental neutron activation analysis (INAA) [22]. Multi-element profiling using inductively coupled plasma optical emission spectroscopy (ICP-OES) and inductively coupled plasma mass spectrometry (ICP-MS) are the most widely used techniques, as they allow the simultaneous determination of many elements in a single sample weighing less than 1 g [27,28,29]. In addition to quantitative multi-element analysis, ICP-MS allows the precise identification and measurement of specific isotopes of the element being analyzed. The analysis of non-metals can also be carried out with very good sensitivity. Additional advantages such as high accuracy, measurement precision, and minimal interferences mean that ICP-MS exceeds the capabilities of other analytical techniques for trace analysis of biomedical samples.

Our previous work in the field of metallomics has investigated the accumulation of seven trace elements, including Ca, Co, Cr, Cu, Fe, K and Mg, Mn, Na, Zn, and Se in the liver of people with alcohol use disorder (AUD) [30,31], the elemental composition of the meninges, i.e., dura mater and arachnoid [32], the optic chiasm by ICP-MS [33] and the aqueous humor of cataract patients [34,35,36]. Other authors have also used ICP-MS to analyze selected metals. Krebs et al. determined the six elements Fe, Cu, Mg, Mn, Ca, and Zn in samples from 13 areas of the human brain and correlated elemental composition with age and interhemispheric differences [37]. McDonald et al. quantified gadolinium (Gd) used as a contrast agent in magnetic resonance imaging (MRI) [38]. Panayi et al. determined the concentrations of Cd and Zn in brain samples from patients with Alzheimer’s disease and senile involutional cortical lesions [39].

Knowledge of the distribution of elements in tissues is essential for understanding their role in human physiology and the etiology of disease, as well as the threats posed by the human anthropogenic environment. From this perspective, there is a need to establish a multi-element dataset for further use in correlative studies. Trace element levels in human tissues have been investigated over the last decade, but most studies have been limited to a few selected elements. Thanks to ICP-MS, which ensures the quantification of elements with excellent sensitivity and resolution, this platform can be extended to more elements and more specific sites [40]. In this study, tandem ICP-MS (ICP-MS/MS) was used to measure the levels of 51 elements in 180 samples from 15 patients in order to determine antagonistic, neutral, or synergistic relationships between the elements and their affinity for specific tissues. Because of the risk of neurodegenerative diseases, samples were taken from 11 different brain regions and from the liver, which is considered to be a detoxifying, metabolizing, filtering, and storing organ that can communicate with the brain via specific neurons. The aim was also to determine the accumulation of toxic trace metals, which can serve as an indicator of exposure, dietary intake, and poor quality of life in south-eastern Poland.

## 2. Materials and Methods

### 2.1. Population

The tissues analyzed for the metal contents were collected in the Department of Forensic Medicine, University of Lublin, Lublin, Poland. The study was approved by the Local Ethical Committee (Medical University of Lublin, Poland, KE-0254/152/2021, date of approval 24 June 2021), and the tissue collection was approved by the prosecutor’s office. The research was carried out in accordance with The Code of Ethics of the World Medical Association, Declaration of Helsinki, for experiments involving humans. The demographic characteristics of the groups are collected in Table 1. Tissue samples were taken post-mortem from individuals who died suddenly as a result of injuries sustained in road traffic accidents (*n* = 15), where the death occurred outside the hospital before the arrival of medical services, deaths occurred due to thoracic (most commonly aortic injury/rupture due to penetrating/blunt force and penetrating thoracic visceral injuries) and abdominal (blunt/penetrating abdominal trauma) injuries. According to the autopsy report, there were two cases of advanced cerebral atherosclerosis, two pleural adhesions, one cyst in the left kidney, one case of left testicular hydrops, and one scar of left ventricular infarction in the study group. There were no subjects with a history of mental illness. All subjects were residents of the Lublin region.

### 2.2. Sample Collection Procedure

The brain and liver tissues analyzed for elemental content were collected at autopsy. Sampling was performed by qualified forensic pathologists. Samples were collected using plastic knives and plastic forceps, which were disinfected with 5% (*v*/*v*) suprapure nitric acid solution and washed with Ultrapure Millipore Direct-Q 3UV-R (Merck, Darmstadt, Germany) with a resistivity of 18.2 MΩ cm. Each tissue sample of approximately 0.5 g was collected, washed thoroughly with ultrapure water, and blotted onto sterile blotting paper. Liver samples were taken from the 6th intercostal space. Brain samples were taken from eleven brain areas: A—polus frontalis (frontal pole), B—gyrus precentralis (precentral gyrus), C—gyrus postcentralis (postcentral gyrus), D—cortex cingularis (gyrus cinguli cingulate gyrus), E—hippocampus (hippocampus), F—caput nuclei caudati (head of caudate nucleus), G—fasciculus longitudinalis superior cerebri (superior longitudinal fasciculus of brain, SLF), H—fasciculus longitudinalis inferior cerebri (inferior longitudinal fasciculus of brain, ILF), I—thalamus dorsalis (dorsal thalamus), J—nucleus accumbens septi (nucleus accumbens septi, NAc), K—insula (insula), L—hepar (liver). Sample storage containers were made of metal-free materials, namely sterile polypropylene containers (Thermo Fisher, Waltham, MA, USA). Parts of the brain under investigation are presented in Supplementary Materials (Figure S3). All samples were weighed and stored at −80 °C until further analysis. Two independent measurements were taken for each sample. The final experimental results were presented as an arithmetic mean.

### 2.3. Sample Preparation

The procedure of the human tissues pre-treatment was proposed previously [30,31,32,33]. In brief, wet mineralization of each 0.3–0.5 g sample was performed via the addition of 7 mL of 69% HNO_3_ ultrapure for trace metal analysis (Baker, Radnor, PA, USA), followed by heating to 190 °C in closed Teflon containers in the microwave mineralization system Multiwave 5000 (Anton Paar, Graz, Austria). After mineralization, 1 mL of 35% HCl ultrapure for trace metal analysis (Baker, Radnor, PA, USA) was added to stabilize some elements (As, Hg, Se, Mo, Tl, Ag). Finally, the samples were diluted to 25 mL with ultrapure water obtained in the purification system.

### 2.4. ICP-MS Measurements

Agilent 8900 ICP-MS Triple Quad (Agilent, Santa Clara, CA, USA) was employed for analysis of 51 elements (^9^Be, ^23^Na, ^24^Mg, ^27^Al, ^31^P, ^39^K, ^44^Ca, ^47^Ti, ^51^V, ^52^Cr, ^55^Mn, ^56^Fe, ^59^Co, ^60^Ni, ^63^Cu, ^66^Zn, ^71^Ga, ^85^Rb, ^88^Sr, ^90^Zr, ^75^As, ^75→91^As, ^78^Se, ^78→94^Se, ^95^Mo, ^105^Pd, ^107^Ag, ^111^Cd, ^118^Sn, ^121^Sb, ^133^Cs, ^137^Ba, ^139^La, cerium ^140^Ce, praseodymium ^141^Pr, neodymium ^146^Nd, samarium ^147^Sm, europium ^153^Eu, ^157^Gd, terbium ^159^Tb, dysprosium ^163^Dy, holmium ^165^Ho, erbium ^166^Er, thulium ^169^Tm, ytterbium ^172^Yb, ^178^Hf, ^195^Pt, ^201^Hg, ^202^Hg, ^205^Tl, ^208^Pb, ^209^Bi, ^232^Th, ^238^U). Most elements were analyzed in He mode (5.5 mL min^−1^ helium flow). Se and As were analyzed in O_2_ mode (gas O_2_ flow rate-30%). The plasma was working in general-purpose mode with 1.550 kW RF power, the nebulizer gas flow was 1.07 L min^−1^, the auxiliary gas flow was 0.9 L min^−1^, and the plasma gas flow was 15 L min^−1^. Acquisition time was from 0.1 to 2 s, depending on the predicted concentration of the element. Due to the lack of certified reference material, the internal standard ISTD (scandium ^45^Sc, ^89^Y, ^89→105^Y, lutetium ^175^Lu) Inorganic Ventures, Christiansburg, VA, USA, with a concentration of 0.5 µg g^−1^ was used for the analysis. ISTD was added automatically using a standard mixing connector, the so-called mixing tee. The obtained recoveries were in the range of 80–120%. ICP commercial analytical standards were purchased from Agilent Technologies, Santa Clara, CA, USA (Multi-Element Calibration Standard 2A-Hg, Environmental Calibration Standard, Multi-Element Calibration Standard 2A), Merck Millipore, Darmstadt, Germany (ICP-Multi-Element Calibration Standard XVII, ICP-Multi-Element Calibration Standard VI, P ICP standard), Honeywell Fluka™, Charlotte, NC, USA (Platinum Standard for ICP, Palladium Standard for ICP), and Inorganic Ventures, Christiansburg, VA, USA (Rare Earth, Standards). The validation report, including background equivalent concentration-BEC, detection limit-DL, ISTD, calibration equation, and the correlation coefficient R, together with individual calibration curves, is presented in Supplementary Materials (Figure S1).

### 2.5. Statistics

To describe the measured quantities, the median was used as a measure of central tendency, and the interquartile range (IQR) [41] was used to assess dispersion since almost all element concentrations had asymmetric distributions. The median is less affected by extreme values and can be a more robust measure in non-normal distributions [42].

The Kruskal-Wallis test, which is a non-parametric counterpart of the ANOVA test, was used to compare the concentration of individual elements between different brain areas. This test was used because the assumptions of the ANOVA test of normality of the distribution of the dependent variable across groups and homogeneity of variance were not met [43]. The effect size of the brain areas was measured using ${\hat{\varepsilon }}_{ordinal}^{2}=\frac{H}{\left(n^{2}-1\right)/\left(n-1\right)},$ where H is the value obtained from Kruskal-Wallis test and n in the total number of observations [44]. If the effect of brain areas was significant, Dunn’s post-hoc tests were conducted [45], with correction for the number of repeated tests by Holm [46].

When the genders were compared in terms of elemental concentration, the Wilcoxon-Mann-Whiteney test was used. The application of this test was dictated once again by the fact that the assumptions of the *t*-test were not met [47].

Pearson’s correlation coefficient was used to test the correlation between the concentrations of each element in the brain and liver areas.

All statistical analyses were performed using the R (2023) [48] statistical environment and libraries that allow for the aforementioned analyses [48,49,50,51,52].

## 3. Results

### 3.1. Descriptive Statistics for ICP-MS/MS Elemental Measurements

Descriptive statistics of the analyzed measurement dataset are provided in Supplementary Materials (Table S1), where the results of the medians, quartile ranges, and the mean values of the two independent measurements, together with the standard deviations, are collected.

The macroelements such as Na, Mg, P, K, Ca, Fe, and Zn occurred in the investigated tissue samples with the highest concentration exceeding 80 µg g^−1^. The group of elements with concentrations higher than 0.1 µg g^−1^ and lower than 80 µg g^−1^ includes Al, Mn, Cu, Rb, Sr, Se, Ba, Ce, Mo, Ti, Tl, Sn, Ni, Cr, Bi, Zr, while the remaining elements were quantified at a concentration lower than 0.1 µg g^−1^. In this group, the rare earth metals were quantified at the lowest content levels in fractions of the 1 ng g^−1^ units.

**Figure 1:**
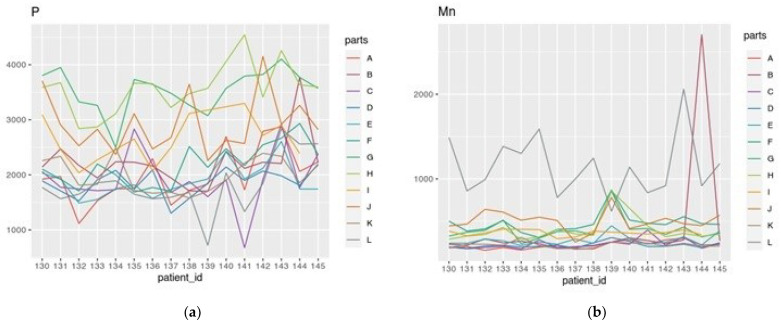
The content of P (**a**) and Mn (**b**) (mean values from two independent measurements) in ng g^−1^ values in the different brain (A–K) and liver (L) tissue samples taken from the investigated subjects (*n* = 15, ID number 130–145).

Among the collected dataset, very high individual variability can be observed in the content of individual elements. The greatest variation concerns macroelements such as Na, Mg, P (Figure 1a), and K, the microelements such as Cu, and the toxic metals Sr, Al, Cs, and Hg. Moreover, significantly higher levels of elements such as Mn (Figure 1b), Fe, Co, Zn, Se, Mo, and Cd are detected in the liver compared to the brain.

P is found in large amounts in various brain structures and liver tissue. However, it can be seen that the brain contains much higher levels of P, which is an important component of phospholipids. The highest P content was found in the superior longitudinal fasciculus and the inferior longitudinal fasciculus, 3541.54 and 3556.00 µg g^−1^, respectively. For comparison, the liver sample was found to be almost twice as low at 1899.47 µg g^−1^. Ca, on the other hand, is present at a relatively constant level of around 100 µg g^−1^ in the tissues examined. It should be noted that the largest difference in Mg levels between tissues is slightly more than 20%. The highest concentration of Mg was found in areas of the brain, such as the superior longitudinal fasciculus and the inferior longitudinal fasciculus, and the nucleus accumbens, which is the main component of the basal ganglia. Zn was found in liver samples at an average concentration around three times higher than in samples from different parts of the brain. However, there were two cases in the study group where Zn levels were an order of magnitude higher than the others. The autopsy report showed that these patients had advanced cerebrovascular atherosclerosis. Significantly elevated levels of Zn were found in three regions of the brain: the precentral gyrus, the cingulate gyrus, and the inferior longitudinal fasciculus.

**Figure 2:**
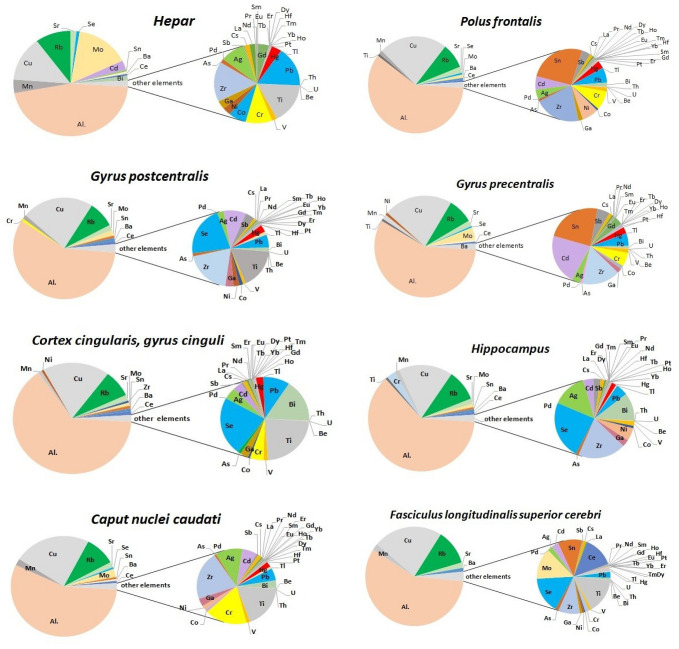

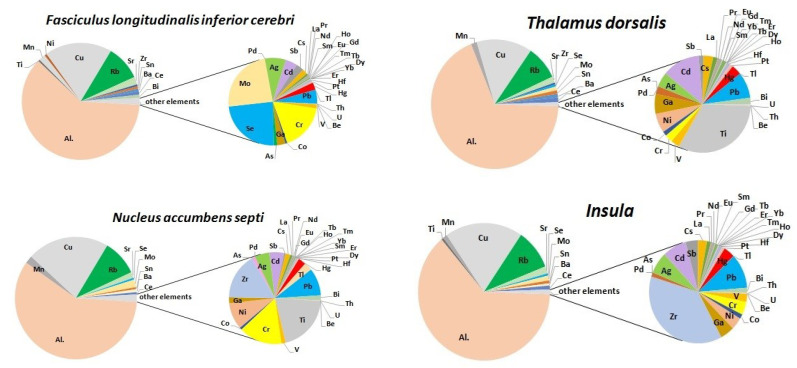
Pie of pie chart showing the percentage of 44 elements (mean values from two independent measurements) in 12 different tissue samples taken from the investigated subjects (*n* = 15). The series was split by a value of 100 ng g^−1^.

Pie charts were used to compare the proportions of elements and to visualize their percentages (Figure 2). Elements such as Ca, K, Na, P, Mg, Zn, and Fe, which are present in the highest concentration, were removed from the plot to allow showing the proportions of trace elements in the examined tissue.

As shown in Figure 1 and Table S1, all brain samples contain significant amounts of Al above 100 ng g^−1^. The highest levels were measured in the thalamus dorsalis and insula samples. The average levels of Al in the tissues are similar. It is noteworthy that in the case of Al, a significant dispersion of results, from a fairly constant level observed in the precentral gyrus (5510.75 ± 1.1 ng g^−1^) to quite varied for the nucleus accumbens (12,894.81 ± 9420.16 ng g^−1^) are observed. In addition to Al, Cu, Rb, Mn, and Sr were detected in all samples above 100 ng g^−1^. High concentrations of Ba, Ce, and Se were also found. Other elements occasionally detected include Mo, Sn, Cr, Ni, Bi, Zr, Tl, and Ti. Cd was found in liver samples.

### 3.2. Are There Any Statistically Significant Differences in the Levels of the Individual Elements between the Brain Areas under Investigation (A to K)?

As the data distributions are not normal, the Kruskal-Wallis test was used to test the hypothesis of equality of concentration of individual elements in different brain areas. Of all the elements tested, only a few (Na, Mg, Al, P, K, Ca, V, Mn, Fe, Co, Cu, Zn, Rb, Se, Mo, Cs, and La) showed significant differences between the different brain areas (Table S2). Comparison graphs are plotted for those elements that differed significantly between brain areas (Figure S2). The test result is shown above each graph, together with an estimate of the effect size (${\hat{\varepsilon }}_{ordinal}^{2}$). The results of Dunn’s post-hoc test, expressed by whiskers connecting significantly different groups, and the *p*-value with Holm’s correction for multiple comparisons are plotted.

In some graphs, no significant differences can be observed in post-hoc tests (no whiskers connecting the groups) (Al, V). This is the case for items where the significance (the *p*-value) of the global test (comparing all brain areas together) is only slightly less than 0.05. In such cases, post-hoc tests may fail to detect the significant differences because the Holm correction overestimates the *p*-value.

**Figure 3:**
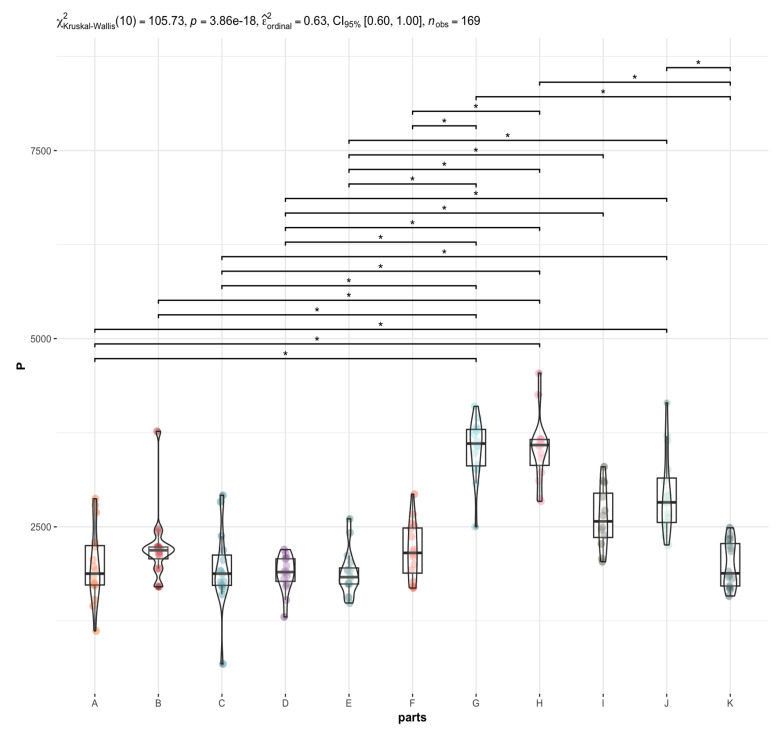
Statistically significant differences in the P content in different brain areas (A to K) with the Kruskal-Wallis test results. The whiskers connect significantly different groups (*p*_Holm-adj_. = 0.03 − 7.36 × 10^−7^). An asterisk symbol (*) indicates statistically significant differences, *p* < 0.05.

**Figure 4:**
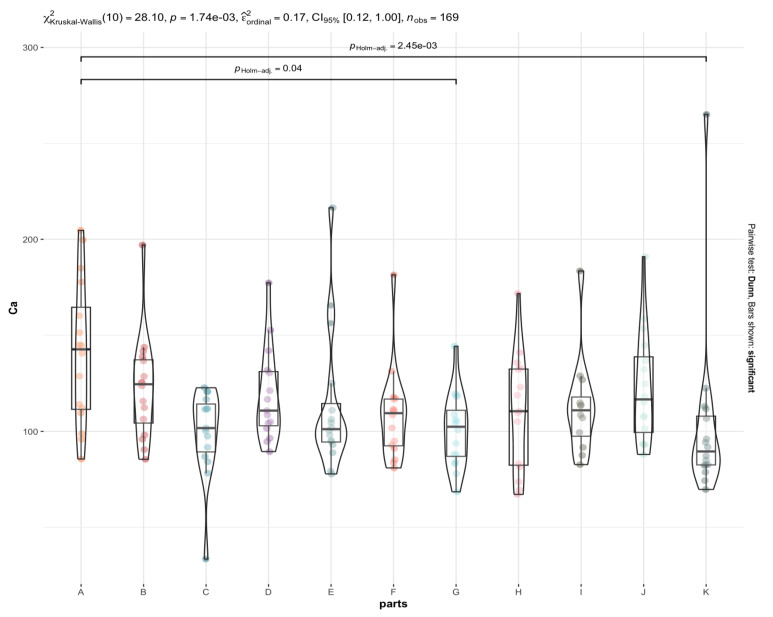
Statistically significant differences in the Ca content in different brain areas (A to K) with the Kruskal-Wallis test results and the *p*-value with Holm’s correction for multiple comparisons. The whiskers connect significantly different groups.

**Figure 5:**
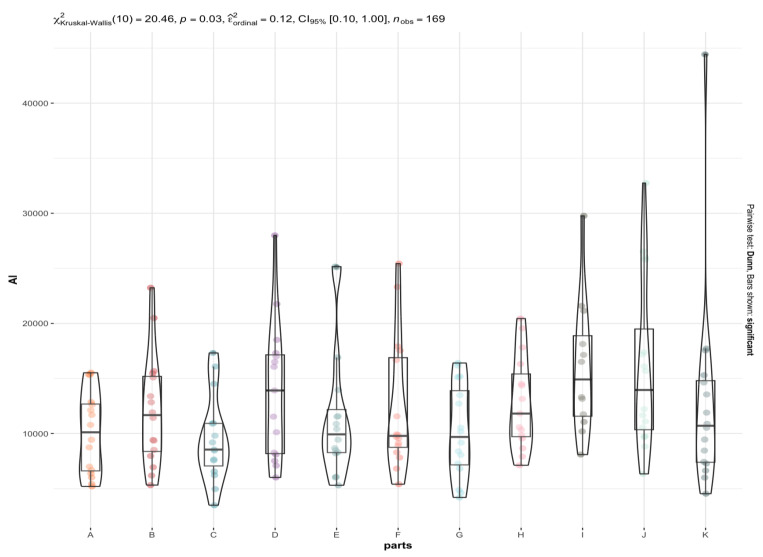
Statistically significant differences in the Al content in different brain areas (A to K) with the Kruskal-Wallis test results.

The most statistically significant differences between elemental contents in different brain regions are observed for elements such as P (Figure 3), Mn, Fe, and Mo, and to a lesser extent for Na, Mg, Cu, Zn, Rb, K, Cs, and Se. In contrast, the least significant differences were observed for Ca (Figure 4) Co, and La. For example, the La content is significantly higher in the area of the nucleus accumbens than in the postcentral gyrus, while the Ca content is significantly higher in the frontal pole than in the superior longitudinal fasciculus and an insula. Co has the highest affinity for the head of the caudate nucleus and nucleus accumbens, while it has the lowest affinity for the superior longitudinal fasciculus, which connects the corners of the dorsolateral surface of the brain. With regard to the other elements, it can be concluded that their distribution in the brain is rather homogeneous (Table S2, Figure 2, Figure 3, Figure 4 and Figure 5). Examples of statistical analyses performed for an element whose content is highly variable in different brain areas (P), weakly variable (Ca), and fairly uniform (Al) are shown in Figure 3, Figure 4, and Figure 5, respectively.

As far as V and Al (Figure 5) are concerned, they appear to be uniformly distributed in the brain region. However, it should be noted that the highest concentrations of both metals were observed in the thalamic area.

**Figure 6:**
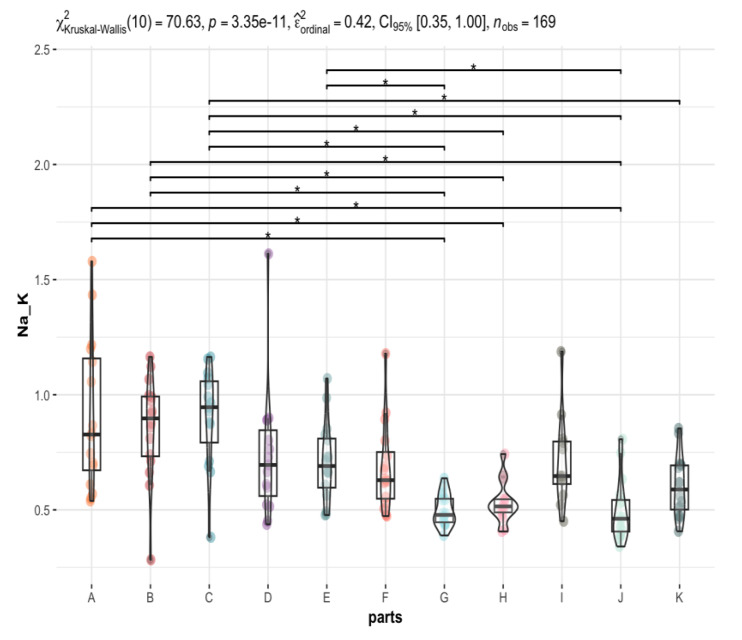
Statistically significant differences in the Na/K ratio content in different brain areas (A to K) with the Kruskal-Wallis test results. The whiskers connect significantly different groups (*p*_Holm-adj_. = 0.05 − 9.6 × 10^−5^).

As mentioned earlier, there are statistically significant differences in Na and K content in different parts of the brain. The ratio of Na to K is also significantly different between the 12 pairs of areas compared (Table 2, Figure 6). This shows that different brain regions have different requirements for these essential macroelements.

### 3.3. Are Elemental Levels in Different Areas of the Brain Different for Men and Women?

Most of the differences between the male and female groups in the elemental content of the different brain areas concern the rare earth metals of the lanthanide group: Ce, Pr, Nd, promethium (Pm), Sm, Eu, Tb, Dy, Ho, Er, Tm, Yb. Men have higher levels of these metals in the brain regions examined. Metals from the actinide group Th, U were also found in significantly higher concentrations in the male group than in the female group in most of the areas examined (C, D, E, F, G, K). There were also significant differences between the groups in the levels of Na, particularly in areas A, E, F, G, H, and K, where men had significantly higher levels of this metal than women. A detailed statistical analysis can be found in Supplementary Materials (Table S3). The elements for which there are statistically significant differences between the male and female groups are summarised in Table 3 for each brain area (A to K). As can be seen, the least significant differences in the elemental composition of the tissues studied between the male and female groups concern the liver and the cingulate gyrus and the inferior longitudinal fasciculus of the brain.

### 3.4. What Are the Correlations between the Content of the Different Elements in the Liver (L) and the Different Brain Areas (A to K)?

The correlation matrices between the total concentration of elements in the whole brain (A to K) and the liver samples are shown in Table 4. As can be seen, there is a statistically significant difference between the contents of these two organs (*p* < 0.05).

**Figure 7:**
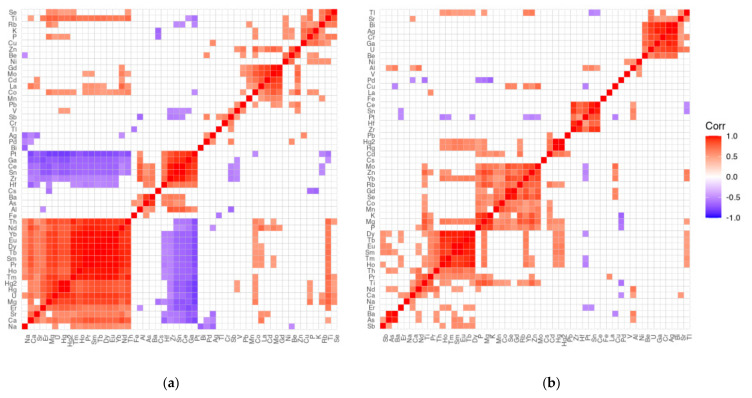
Spearman rank-order correlation matrix for the (**a**) brain and (**b**) liver.

The correlation matrices (Figure 7a,b), generated separately for the brain and liver, show the existence of positive (red) and negative (blue) correlations, expressed by the Spearman rank order correlation coefficient. In the liver, there are few positive correlations, which are weak in strength, whereas in the brain, both positive and negative correlations appear in larger numbers.

It is possible to statistically analyze the significance of correlations between different brain areas and liver samples individually. As there are many such correlations for a fixed pair of areas within each element, they have been included in Supplementary Materials (Table S4).

Most of the correlations are positive; only a few are weakly negatively correlated. The strongest positive correlations (R > 0.8) are as follows: A: Se (0.9338), Ce (0.8305); B: As (0.8690), Ba (0.8739); D: Se (0.9247); E: Se (0.9166), Ce (0.9043), Sn (0.8082); F: Zr (0.8372), Se (0.9394), Sn (0.8929), Ce (0.8769), Pt (0.8260), U (0. 8821); G: Ni (0.9537), Se (0.9034), Cs (0.8064), Bi (0.9222); H: Se (0.9003), Ag (0.9791), Ce (0.8822), Pt (0.8574), Bi (0.999); I: Se (0.8707); J: Se (0.8242), Sn (0.8110), Dy (0.8199); K: Se (0.9330), Pd (0.9996).

The strongest negative correlations occurred for the few elements between their liver content and the following brain areas: A: V (−0.2907), B: Nd (−0.2257), C: Sr (−0.4251), D: La (−0.5110), E: Nd (−0.2435), F: Ca (−0.2431), G: La (−0.3588), H: Mn (−0.4316), I: V (−0.3157), J: Ba (−0.2254), K: Cr (−0.2832).

## 4. Discussion

### 4.1. P in the Brain vs. Liver

The study observed a high accumulation of P in the brain compared to its levels in the liver. In addition, statistical analysis showed that there were many significant differences in its content between different areas of the brain. Thus t distribution of P in the brain is not homogeneous. The highest levels are found in the superior longitudinal fasciculus and the inferior longitudinal fasciculus. The superior longitudinal fasciculus is the largest associative fiber bundle system in the brain that connects large areas of the frontal and parietal lobes [54]. As such, it is likely to be involved in multiple functional correlates. It is known that there is enormous variation in SLF volume within the population, which would seem to explain the observed high individual variability in P content.

### 4.2. Xenobiotic Toxic Metals

In addition to micronutrients, xenobiotic toxic metals are also absorbed and can accumulate in tissues, leading to structural and physiological damage. Of particular note is the high average level of Al in the brain. Al is known to be a toxic element for the human body. The toxic effects of Al mainly affect the nervous system because Al can cross the blood-brain barrier and accumulate in the brain.

Al, similar to V, appears to be evenly distributed in the brain region. However, it should be noted that the highest concentrations of both metals were observed in the thalamic area. V, on the other hand, is an essential element that most likely has a regulatory function as part of phosphate enzymes. Due to the relatively widespread presence of V in food sources (e.g., soya, apples, mushrooms, or eggs), there is a likelihood of overdoses resulting in damage to the nervous system, headaches, and behavioral changes, as well as cardiovascular disease or gastrointestinal, renal and hepatic disorders. The presence of V compounds in the air is not irrelevant to the results obtained, as they are contained in the so-called PM2.5 (<2.5 μm) and PM10 (<10 μm) particulate matter, the sources of which include car exhausts and the burning of coal, wood, and oil. The south-eastern part of Poland is characterized by above-average concentrations of particulate matter (higher than 75 µg m^−3^) and has therefore been classified as Zone C [55]. A special air protection program was only implemented in 2013.

As for Al, its content was very high in both brain and liver tissues. Although there were differences between brain areas (A to K) and between individuals, these did not reach statistical significance. There was also no significant correlation between brain and liver Al levels. A similar result was obtained in the previous study, which was a multi-element analysis of post-mortem tissue from the optic chiasm and optic nerves [33]. It should be noted that the presence and importance of Al in the brain is mainly analyzed in relation to neurological disorders, i.e., amyotrophic lateral sclerosis, senile dementia, Parkinson’s disease, and, to some extent, Alzheimer’s disease and autism spectrum disorder (ASD) [56,57,58]. Although it is known that Al can diffuse into the brain by crossing the blood-brain barrier, the specific pattern of its distribution is variable and depends mainly on exposure to the element. There is evidence that Al accumulates in the brain of people with AUD [59], particularly in beer drinkers [60]. Al enters the brain at all stages of a person’s life. A wide range of measured Al levels in brain tissue homogenates can be found in the literature, ranging from 0.1 to 4.5 μg Al g^−1^ tissue (dry weight). It should be emphasized that higher levels, even up to 47.4 μg g^−1^ dry weight, have been reported in neurodegenerative diseases, e.g., Alzheimer’s disease, amyloid angiopathy, Al-related encephalopathies, and others [61]. In our study, the average Al level in the whole brain is about 12.5 μg g^−1^, indicating an accumulation of this element. It may indicate a high exposure to Al of the subjects studied, and a high potential risk of neurogenerative diseases, but information on such exposure was not available.

### 4.3. The Rare Earth Metal Content

The study found statistically significant differences in the brain content of rare earth metals between the male and female groups. These metals belong to a very rarely analyzed group of trace elements. They are found in tissues at very low levels, below 1 ng g^−1^. Aleksandar Stojsavljević et al. [62] published the first study providing compositional levels of essential and toxic trace elements, rare earth elements, and noble metals in human placental tissues. The concentration of rare earth elements in the tissues reported in this paper is similar to our study and is less than 1 ng g^−1^. In the study by Baj et al. [32], the meninges, i.e., the dura mater and the arachnoid, were analyzed for the first time for their elemental composition. Significant differences were found in metals from the lanthanide family. The authors found that there is a significant affinity of elements from the lanthanide family to the arachnoid meninx compared to the dura mater. Advances in modern medical technology have increased the potential human exposure to lanthanides. It appears that both the central and peripheral nervous systems are highly susceptible to their effects. This is due to the fact that the trivalent ion radii of the lanthanides (from La^3+^ 122 to Lu^3+^ 85 pm) are similar to the key signaling ion of the divalent calcium ion (Ca^2+^ 106 pm). Lanthanide cations can block ion channels in neurons and regulate neurotransmitter turnover, release, and synaptic activity. Lanthanides also act as modulators of several ionotropic receptors and can affect numerous signaling mechanisms and the apoptosis-related endoplasmic reticulum pathways [63]. Several lanthanide ions can cause oxidative neuronal damage and functional impairment by promoting the production of reactive oxygen species. In contrast, Ce and Y oxides have some promising neuroprotective properties, being able to reduce free radical damage to cells and even alleviate motor impairment and cognitive function in animal models of multiple sclerosis and mild traumatic brain injury. In summary, lanthanides affect a variety of neurophysiological processes and alter a wide range of brain functions. Therefore, detailed research is needed to elucidate the mechanism of their neural functions and to find the reason for the difference in their levels in male and female brains.

### 4.4. The Strongest Positive Intercorrelation between Brain and Liver for Se

Only Se showed a very strong positive correlation between brain and liver samples, regardless of sampling location. It should be noted that global Se levels in the brain (106 ± 19.51 ng g^−1^) were almost twice as low as in liver tissue (236 ± 191 ng g^−1^).

Se in the body is not stored in any organ, and its excess is excreted from the body mainly by methylation, leading to the formation of methylselenol and 1-β-methylseleno-*N*-acetyl-D-galactosamine. Even the symptoms of selenosis caused by excessive Se intake are reversed when supplementation is reduced [64]. The different distribution of this element in the brain and liver is rather due to different requirements for this element. Our work is in agreement with the experiment carried out on rats [65]. Buckman et al. found that Se levels in the liver and brain tissue increased with increasing dietary intake. It was observed that when the dietary supply of Se is limited, there is a loss of this element as well as a decrease in the activity of the selenoenzyme glutathione peroxidase (GPx) in the liver, but not in the brain, where sequestration of this element takes place. In contrast, high Se intake did not increase GPx in any tissue compared with normal intake, but Se was retained in brain tissue. In the liver, Se retention was greater in rats fed a normal diet compared with animals fed a low or high Se diet [65]. The higher amount of Se in the liver, therefore, indicates that the Se supply in the study group was adequate and that the subjects were not exposed to excess Se.

### 4.5. The Strongest Negative Intercorrelation between Brain and Liver for Mn and La

In this study, negative correlations were found between the brain levels of Mn (−0.4316) and La (−0.5110) and their levels in the liver. More than tripled Mn (1144/332.8 ng g^−1^) and more than quadrupled La (12.72/3.05 ng g^−1^) in the liver may indicate a liver need for these elements or increased exposure to them. While the role of Mn has been extensively reported in the literature, little is known about the requirements and functions of La [63]. As an essential element, Mn is involved in the activation of enzymes such as arginase, glutamine synthetase (GS), pyruvate carboxylase, and Mn superoxide dismutase (Mn-SOD) and is involved in the metabolism of glucose, lipids, and proteins. As an antioxidant, Mn is involved in reducing oxidative stress [66]. Other authors have also reported Mn accumulation in several mitochondria-rich organs, e.g., the pancreas and liver [67,68]. However, excess Mn in the brain is detrimental and can cause Parkinson’s-like symptoms [69]. Previous studies have shown that Mn can cross the blood-brain barrier and accumulate most in the hypothalamus or pituitary gland [70]. In our study, the affinity of different brain areas for Mn can be ordered as follows: insula > hippocampus > precentral gyrus~head of caudate nucleus.

## 5. Conclusions

Multi-element analysis of various brain areas and liver tissue samples taken at autopsy helped to establish ranges for 51 elements in the population of Lublin residents studied. On the basis of ICP-MS measurements, it was found that (i) an alarmingly high level of Al in the brain, which, despite high individual variability, was evenly distributed in different brain areas; (ii) the greatest variation in requirement was shown by P, the highest amount of which was detected in the superior longitudinal fasciculus and the inferior longitudinal fasciculus of the brain; (iii) the levels of elements determined in the liver were statistically significantly different from their content in the brain, with a particular preference for the liver shown by Mn, Fe, Co, Zn, Se, Mo and Cd; (iv) some of the inter-elemental correlations (liver vs. brain) reached high positive correlation coefficients, e.g., for Se, only a few were weakly negatively correlated, e.g., for Mn; (v) there were no statistically significant differences in the elemental composition of the liver between males and females, but these groups differed in the levels of lanthanides and actinides in the brain. The determination of metal concentrations in autopsy tissues can be useful for multi-element mapping and for learning about inter-element correlations between different organs. Future studies should include groups selected not only by place of residence, age, gender, type of addiction but also, and most importantly, by disease entities, which will shed new light on their etiology and progression.

## Figures and Tables

**Table 1:** The demographic characteristic of the groups enrolled in the study.

Population	Gender	%	BMI ^1^	Min-Max Age	Median Age	Mean Age ± SD
*n* = 15	Female *n* = 4	26.67	21.85 ± 4.54	20–41	33.0	31.33 ± 10.59
	Male *n* = 11	73.33	27.29 ± 6.98	49–86	68.5	66.38 ± 11.90

^1^ Body mass index.

**Table 2:** Statistically significant differences in the Na/K ratio content in different brain areas.

A N = 16	B N = 16	C N = 15	D N = 15	E N = 16	F N = 16	G N = 16	H N = 15	I N = 12	J N = 16	K N = 16	*p*-Value
0.83 (0.67, 1.16)	0.90 (0.73, 0.99)	0.95 (0.79, 1.06)	0.70 (0.56, 0.85)	0.69 (0.60, 0.81)	0.63 (0.55, 0.75)	0.48 (0.45, 0.55)	0.51 (0.49, 0.55)	0.65 (0.61, 0.80)	0.46 (0.41, 0.54)	0.59 (0.50, 0.69)	<0.001

**Table 3:** The elements in each part of the brain (A to K) for which there are statistically significant differences between the male and female groups.

Area of the Brain	Alkali and Alkaline Earth Metals	Transition Metals	Rare Earth Elements	Actinides	*p*-Block Metals	Metalloids	Non-Metals
A	Na	Ti, Cr	Nd, Sm, Eu, Tb, Dy, Er, Tm, Yb	-	Bi	-	-
B	Be	Ti	Sm, Eu, Tb, Dy, Ho, Er, Tm, Yb	-	Bi	-	-
C		Cr	Pr, Nd, Sm, Eu, Tb, Dy, Ho, Er, Tm, Yb,	U	-	-	-
D	Cs	Pt	Pr	Th	-	-	-
E	Na		Pr, Sm, Eu, Tb, Dy, Ho, Er, Tm, Yb	Th	-	Sb	-
F	Na, Sr		Sm, Eu, Tb, Dy, Er, Tm, Yb	U	-	-	-
G	Na	Ni, Zr, Hg, Hf	Pr, Sm, Eu, Gd, Tb, Dy, Er, Yb	U	Sn, Ga	-	-
H	Na	-	-	-	-	-	Se
I	-	Mn, Zr, Mo, Pt	Ga, Ce, Gd	U	Sn	-	-
J	-	Hg	Eu, Dy, Ho, Er, Tm, Yb	-	-	-	-
K	Na, Mg	Ni	Pr, Nd, Sm, Eu, Tb, Dy, Ho, Er, Tm, Yb	Th, U	-	-	-
L	-	Ni, Mn, V	-	U	-	-	-

**Table 4:** Correlation Matrix (pearson-method) between the total concentration of elements in the brain (A to K) vs. the liver samples, *p*-value adjustment method: Holm (1979) [53].

Parameter 1	Parameter 2	r	95% CI	t(50)	*p*
brain	liver	0.99	[0.98, 0.99]	44.33	< 0.001 ***

*** indicates *p* < 0.001, which is statistically highly significant.

## Data Availability

The data presented in this study are available upon request from J.B.

## Supplementary Materials

The following supporting information can be downloaded at: https://www.mdpi.com/article/10.3390/nu15122799/s1, Figure S1: Background equivalent concentration-BEC, detection limit-DL, internal standard-ISTD, calibration equation, and the correlation coefficient R, together with individual calibration curves for elements examined using ICP-MS. Figure S2: Comparative graphs for elements significantly differentiated by their content in the examined areas of the brain. Above each graph, the results of Dunn’s post-hoc tests, expressed by whiskers connecting significantly different groups, and the *p*-value with Holm’s correction for the multiplicity of comparisons, are placed. Figure S3: Parts of the brain under investigation. Table S1: Descriptive statistics covering medians and quartile ranges (Table S1A), mean and standard deviation (Table S1B) for ICP-MS measurements of brain and liver samples taken from the entire population of the studied subjects.; Table S2: Kruskal-Wallis test to test the hypothesis that the concentrations of individual elements are equal in different brain areas. Elements showing significant differences between brain areas are shown in grey; Table S3: Statistical examination of whether the levels of individual elements differ statistically significantly between women and men for the brain regions from [A] to [K] and the liver samples [L]; Table S4: Study of the correlation between the content of individual elements in the liver (L) and various areas of the brain (from A to K).
