# Supplementary material for: Assessment of the Concentration of 51 Elements in the Liver and in Various Parts of the Human Brain—Profiling of the Mineral Status
Source: Nutrients. 2023 Jun 19;15(12):2799. doi: 10.3390/nu15122799 (PMC10302479; doi:10.3390/nu15122799)
Supplement: Supplementary file 1 [file nutrients-15-02799-s001.zip › Figure S1.pdf]

Calibration for D:\ICPMH\1\DATA\mozg1_27-02-23.b\001CALB.d

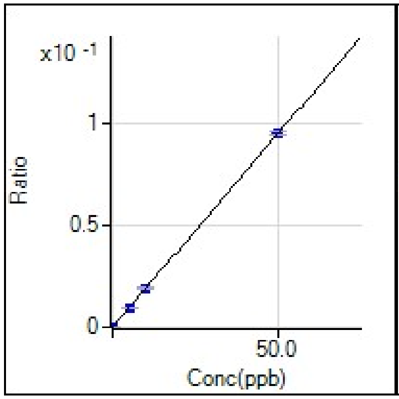

9 Be [No Gas]
ISTD: 45 Sc
y = 1.903E-3 x + 7.566E-6

R	1.0000
DL	0.002121
BEC	0.003976

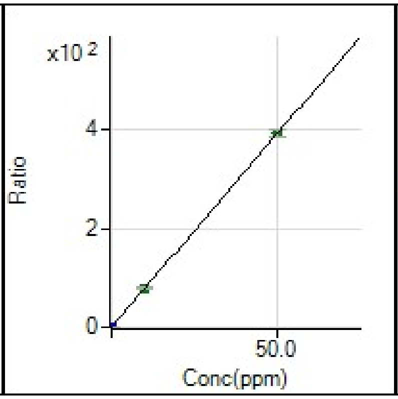

23 Na [He]
ISTD: 45 Sc
y = 7.852E0 x + 3.478E-1

R	1.0000
DL	0.003985
BEC	0.0443

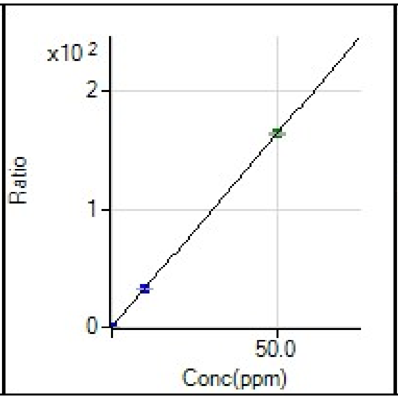

24 Mg [He]
ISTD: 45 Sc
y = 3.280E0 x + 4.907E-3

R	1.0000
DL	0.0008205
BEC	0.001496

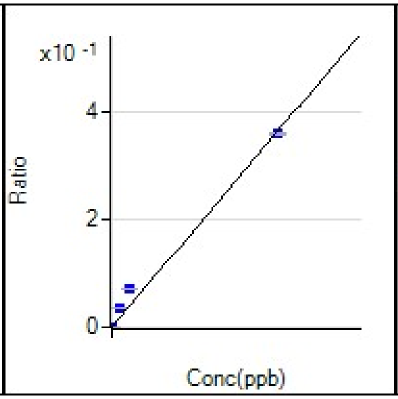

27 Al [He]
ISTD: 45 Sc
y = 3.626E-4 x + 1.012E-3

R	0.9957
DL	1.197
BEC	2.791

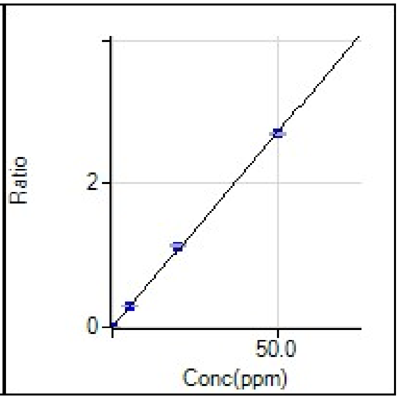

31 P [He]
ISTD: 45 Sc
y = 5.440E-2 x + 1.812E-3

R	0.9998
DL	0.01034
BEC	0.03331

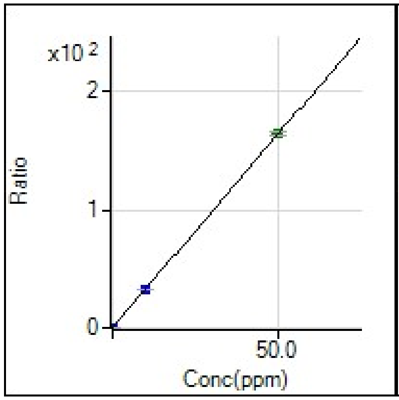

39 K [He]
ISTD: 45 Sc
y = 3.274E0 x + 3.792E-1

R	1.0000
DL	0.006145
BEC	0.1158

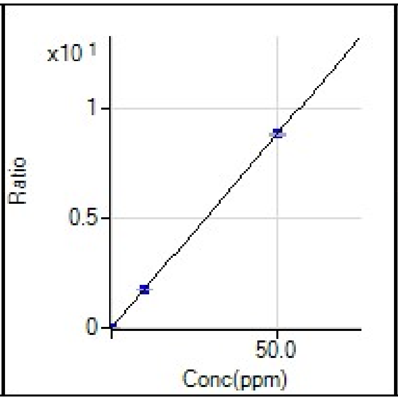

44 Ca [He]
ISTD: 45 Sc
y = 1.768E-1 x + 3.701E-3

R	1.0000
DL	0.003611
BEC	0.02094

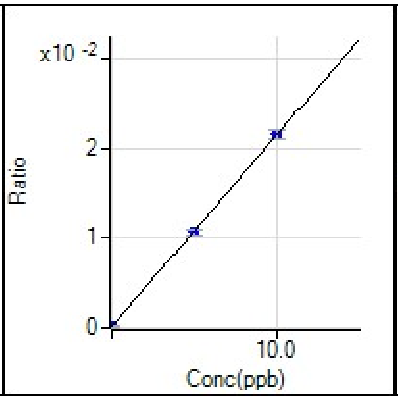

47 Ti [He]
ISTD: 45 Sc
y = 2.134E-3 x + 1.285E-4

R	1.0000
DL	0.0803
BEC	0.06021

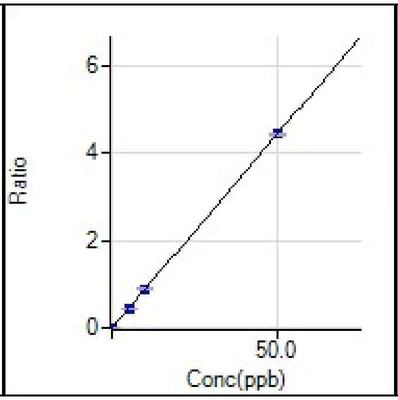

51 V [He]
ISTD: 45 Sc
y = 8.886E-2 x + 3.030E-4

R	1.0000
DL	0.006835
BEC	0.003409

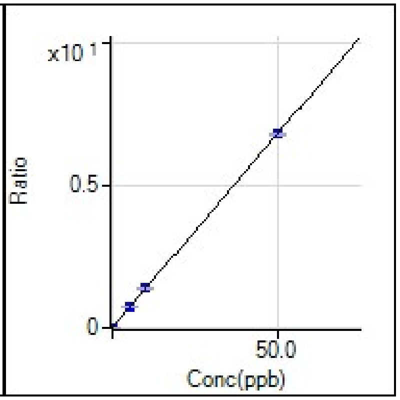

52 Cr [He]
ISTD: 45 Sc
y = 1.356E-1 x + 1.397E-2

R	1.0000
DL	0.03834
BEC	0.103

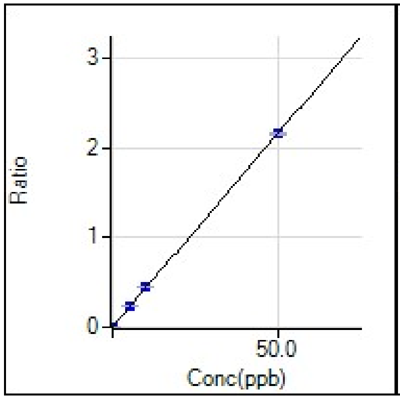

55 Mn [He]
ISTD: 45 Sc
y = 4.325E-2 x + 2.696E-3

R	1.0000
DL	0.04479
BEC	0.06233

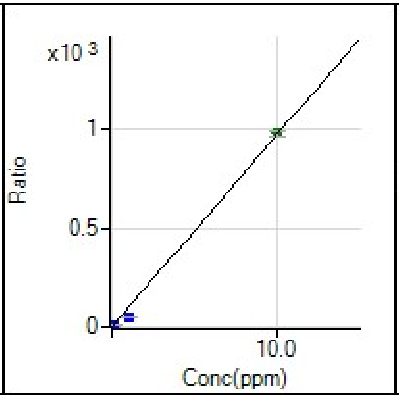

56 Fe [He]
ISTD: 45 Sc
y = 9.766E1 x + 1.412E-1

R	0.9988
DL	3.084E-05
BEC	0.001446

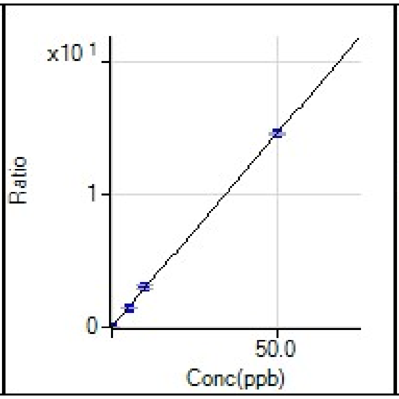

59 Co [He]
ISTD: 45 Sc
y = 2.925E-1 x + 2.537E-2

R	1.0000
DL	0.44
BEC	0.08674

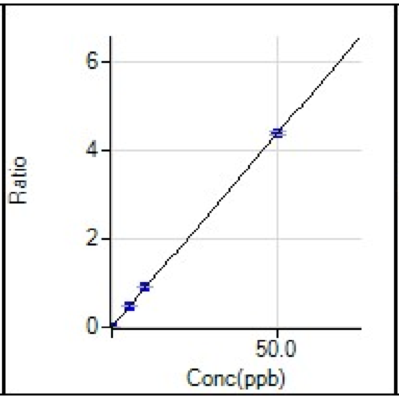

60 Ni [He]
ISTD: 45 Sc
y = 8.748E-2 x + 7.732E-3

R	1.0000
DL	0.03112
BEC	0.08839

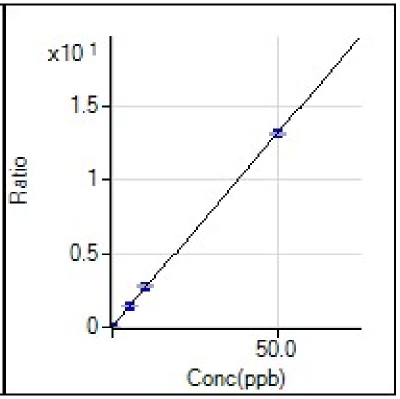

63 Cu [He]
ISTD: 45 Sc
y = 2.618E-1 x + 3.231E-2

R	0.9999
DL	0.02875
BEC	0.1234

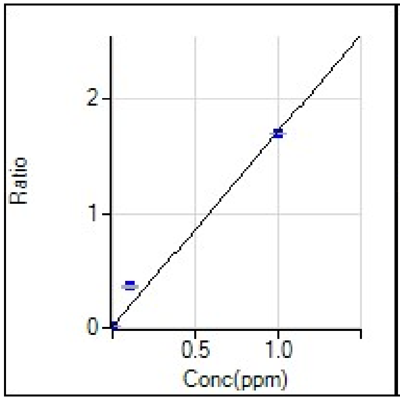

66 Zn [He]
ISTD: 89 Y
y = 1.702E0 x + 1.296E-2

R	0.9942
DL	0.0009462
BEC	0.007612

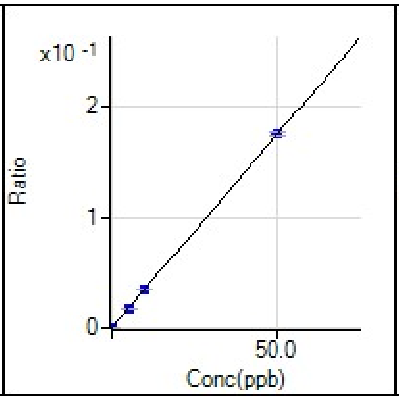

71 Ga [He]
ISTD: 89 Y
y = 3.510E-3 x + 4.641E-5

R	1.0000
DL	0.03435
BEC	0.01322

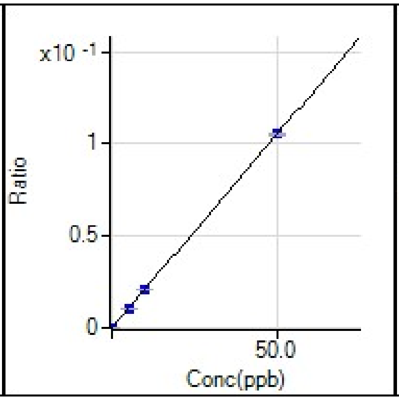

75 As [He]
ISTD: 89 Y
y = 2.105E-3 x + 3.583E-5

R	1.0000
DL	0.005685
BEC	0.01702

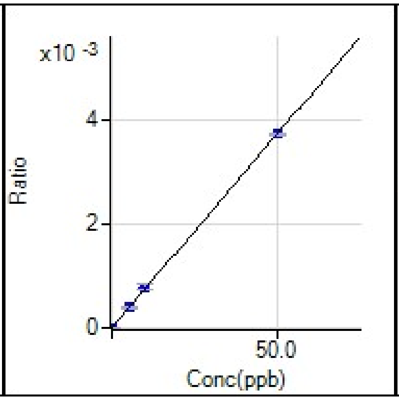

78 Se [He]
ISTD: 89 Y
y = 7.443E-5 x + 6.942E-6

R	1.0000
DL	0.1411
BEC	0.09326

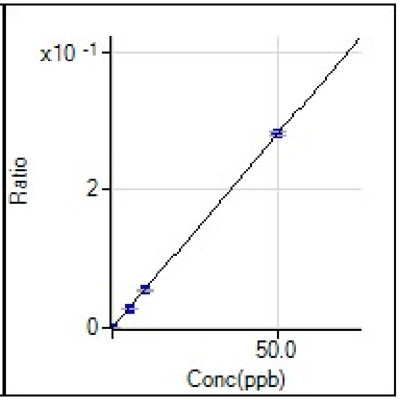

85 Rb [He]
ISTD: 89 Y
y = 5.610E-3 x + 3.120E-4

R	1.0000
DL	0.04905
BEC	0.05562

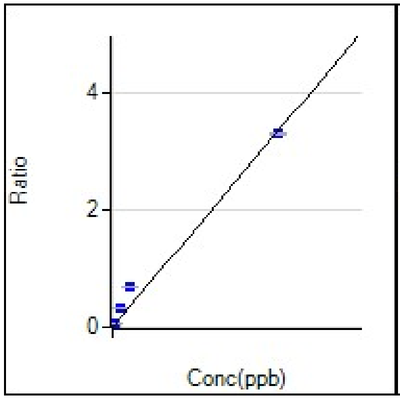

88 Sr [He]
ISTD: 89 Y
y = 3.339E-3 x + 1.965E-3
R 0.9941
DL 0.0471
BEC 0.5885

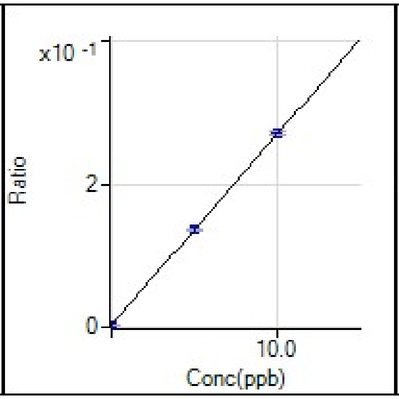

90 Zr [He]
ISTD: 89 Y
y = 2.668E-2 x + 3.282E-3
R 1.0000
DL 0.01889
BEC 0.123

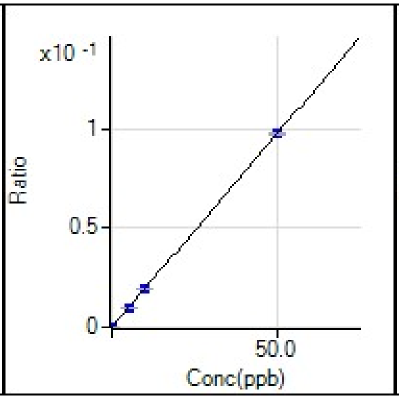

75 -> 91 As [O2]
ISTD: 89 -> 105 Y
y = 1.951E-3 x + 4.062E-5
R 1.0000
DL 0.003771
BEC 0.02082

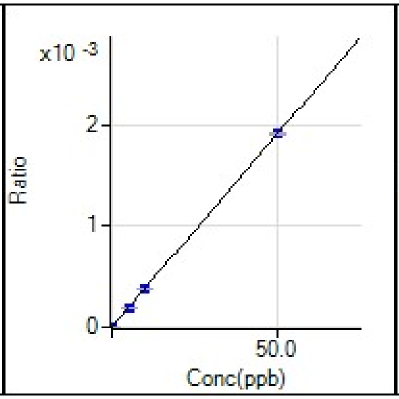

78 -> 94 Se [O2]
ISTD: 89 -> 105 Y
y = 3.816E-5 x + 4.803E-6
R 1.0000
DL 0.08514
BEC 0.1259

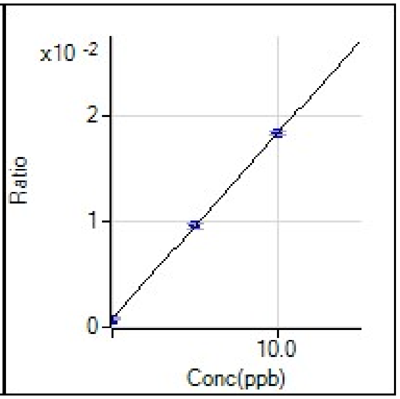

95 Mo [He]
ISTD: 89 Y
y = 1.754E-3 x + 8.165E-4
R 1.0000
DL 0.1182
BEC 0.4656

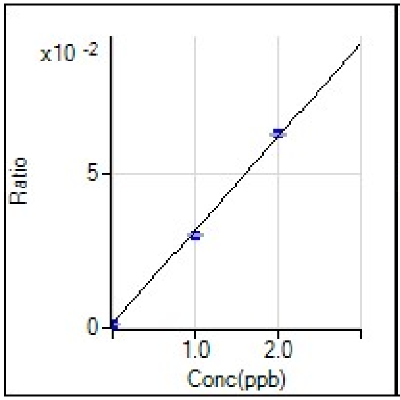

105 Pd [He]
ISTD: 89 Y
y = 3.010E-2 x + 1.510E-3
R 0.9991
DL 0.007312
BEC 0.05016

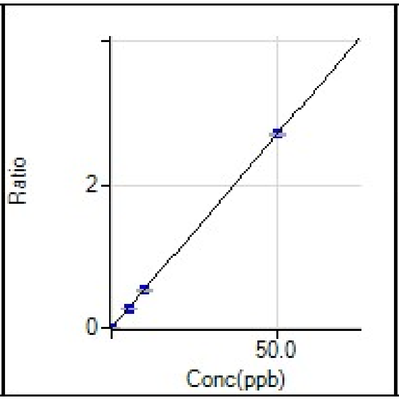

107 Ag [He]
ISTD: 89 Y
y = 5.415E-2 x + 6.548E-4
R 1.0000
DL 0.006478
BEC 0.01209

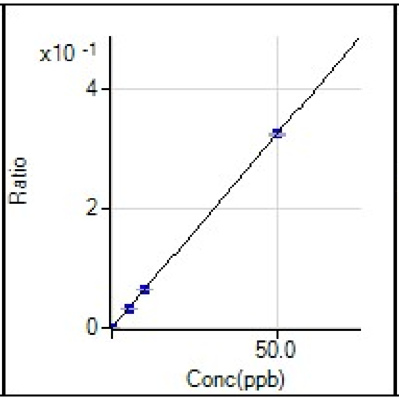

111 Cd [He]
ISTD: 89 Y
y = 6.493E-3 x + 1.037E-5
R 1.0000
DL 0.003168
BEC 0.001597

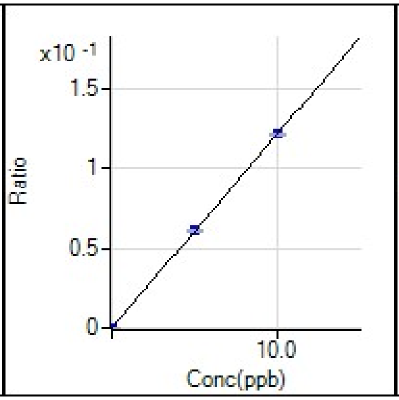

118 Sn [He]
ISTD: 89 Y
y = 1.218E-2 x + 1.003E-4
R 1.0000
DL 0.007261
BEC 0.008234

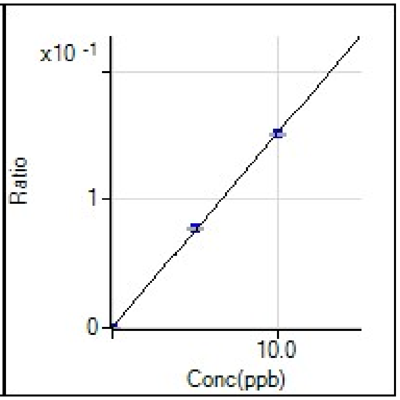

121 Sb [He]
ISTD: 89 Y
y = 1.516E-2 x + 3.546E-4
R 0.9999
DL 0.01288
BEC 0.0234

Calibration for D:\ICPMH\1\DATA\mozg1_27-02-23.b\001CALB.d

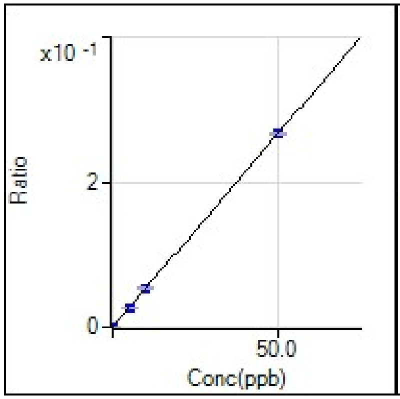

133 Cs [He]
ISTD: 175 Lu
y = 5.351E-3 x + 4.032E-5
R 1.0000
DL 0.005469
BEC 0.007535

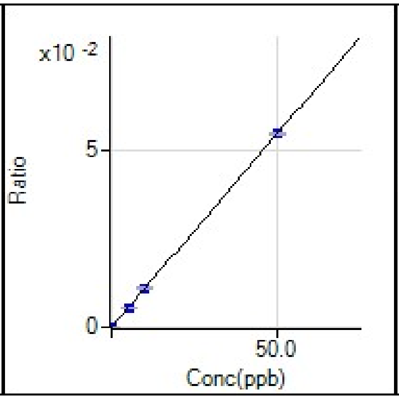

137 Ba [He]
ISTD: 175 Lu
y = 1.098E-3 x + 1.597E-5
R 1.0000
DL 0.02366
BEC 0.01455

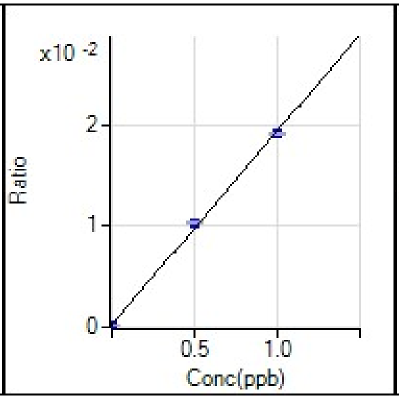

139 La [He]
ISTD: 175 Lu
y = 1.913E-2 x + 2.496E-4
R 0.9993
DL 0.004986
BEC 0.01305

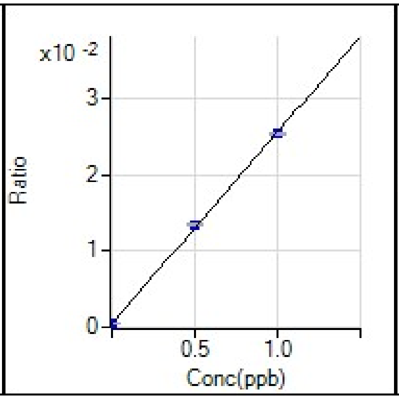

140 Ce [He]
ISTD: 175 Lu
y = 2.518E-2 x + 4.578E-4
R 0.9997
DL 0.004723
BEC 0.01818

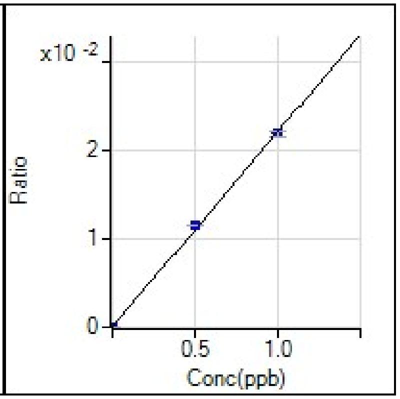

141 Pr [He]
ISTD: 175 Lu
y = 2.202E-2 x + 2.815E-5
R 0.9996
DL 0.001025
BEC 0.001278

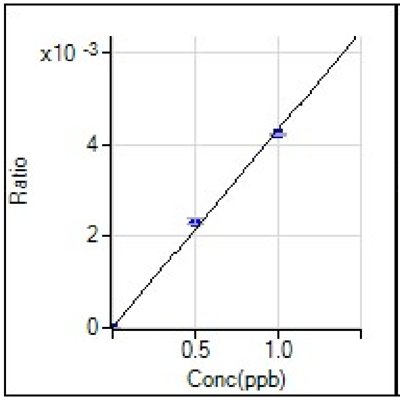

146 Nd [He]
ISTD: 175 Lu
y = 4.310E-3 x + 6.569E-6
R 0.9983
DL 0.001134
BEC 0.001524

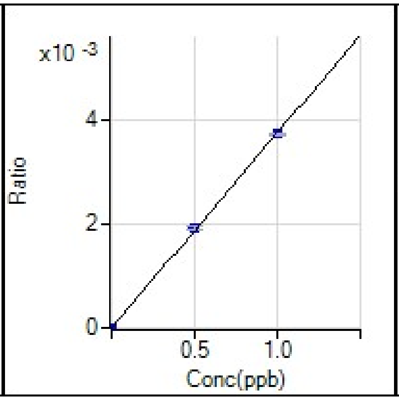

147 Sm [He]
ISTD: 175 Lu
y = 3.740E-3 x
R 0.9998
DL 0
BEC 0

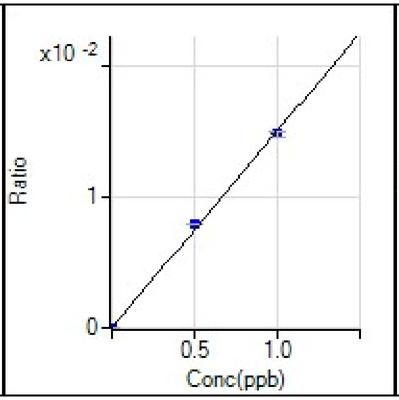

153 Eu [He]
ISTD: 175 Lu
y = 1.501E-2 x + 8.437E-6
R 0.9991
DL 0.0005568
BEC 0.0005622

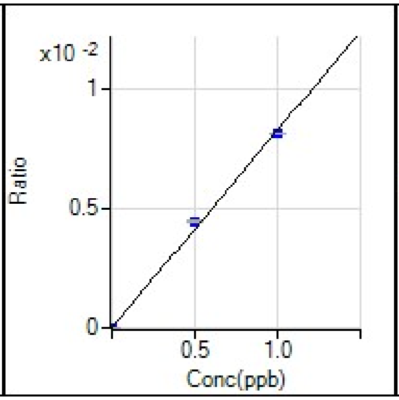

157 Gd [He]
ISTD: 175 Lu
y = 8.280E-3 x + 1.126E-5
R 0.9984
DL 0.002048
BEC 0.00136

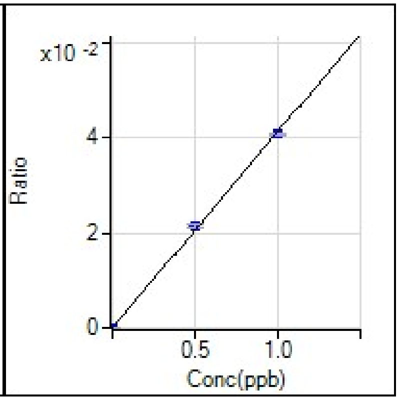

159 Tb [He]
ISTD: 175 Lu
y = 4.124E-2 x + 1.969E-5
R 0.9995
DL 0.000351
BEC 0.0004775

Calibration for D:\ICPMH\1\DATA\mozg1_27-02-23.b\001CALB.d

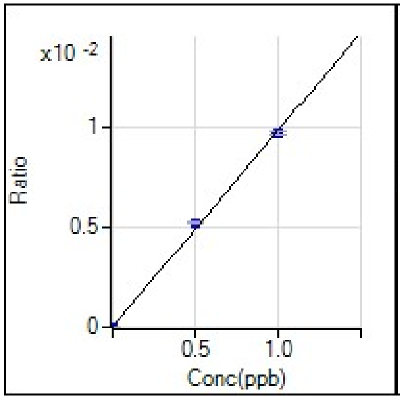

163 Dy [He]
ISTD: 175 Lu
y = 9.799E-3 x + 5.609E-6
R 0.9990
DL 0.00227
BEC 0.0005724

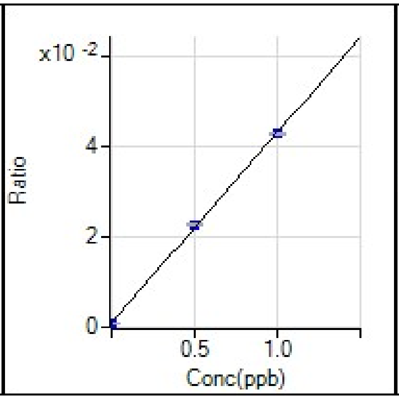

165 Ho [He]
ISTD: 175 Lu
y = 4.221E-2 x + 1.027E-3
R 0.9998
DL 0.003011
BEC 0.02434

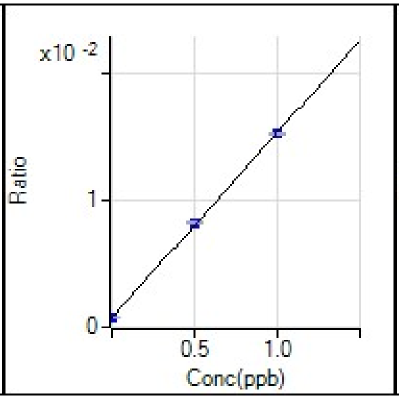

166 Er [He]
ISTD: 175 Lu
y = 1.462E-2 x + 7.522E-4
R 0.9999
DL 0.01275
BEC 0.05146

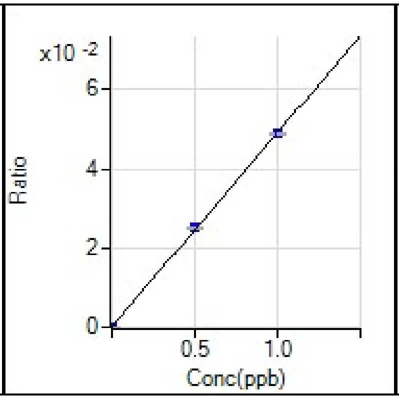

169 Tm [He]
ISTD: 175 Lu
y = 4.901E-2 x + 1.238E-4
R 0.9999
DL 0.001196
BEC 0.002526

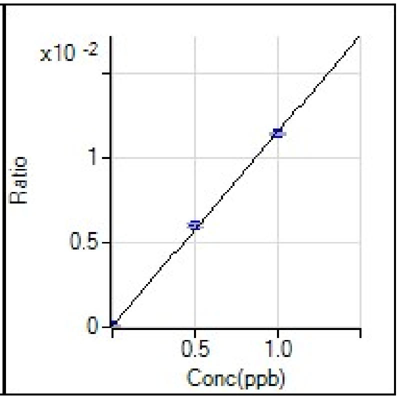

172 Yb [He]
ISTD: 175 Lu
y = 1.148E-2 x + 6.382E-5
R 0.9997
DL 0.004725
BEC 0.005557

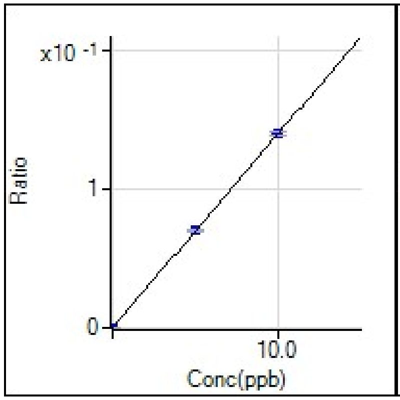

178 Hf [He]
ISTD: 175 Lu
y = 1.395E-2 x + 1.135E-4
R 1.0000
DL 0.005351
BEC 0.008141

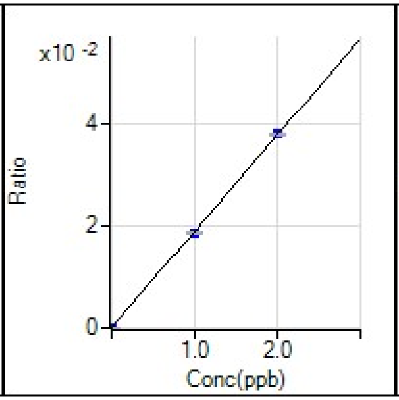

195 Pt [He]
ISTD: 175 Lu
y = 1.882E-2 x + 1.030E-4
R 0.9999
DL 0.001916
BEC 0.005473

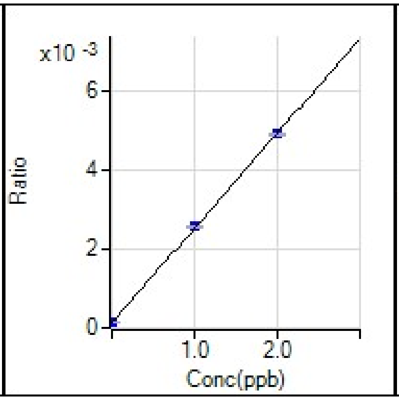

201 Hg [He]
ISTD: 175 Lu
y = 2.392E-3 x + 1.314E-4
R 0.9999
DL 0.01189
BEC 0.05493

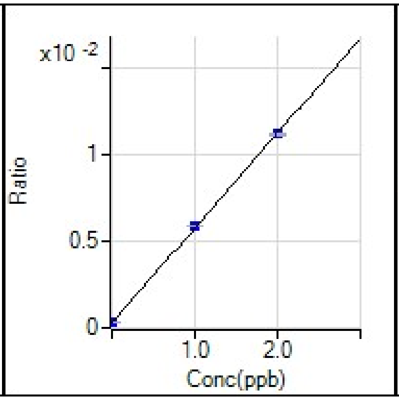

202 Hg [He]
ISTD: 175 Lu
y = 5.492E-3 x + 3.021E-4
R 0.9999
DL 0.01373
BEC 0.05501

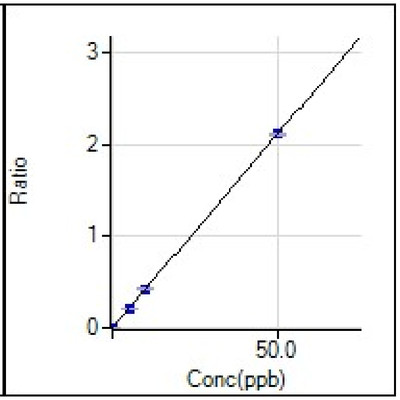

205 Tl [He]
ISTD: 175 Lu
y = 4.237E-2 x + 1.232E-4
R 1.0000
DL 0.0007525
BEC 0.002908

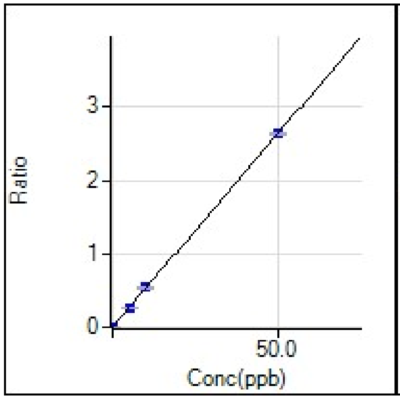

208 Pb [He]
ISTD: 175 Lu
y = 5.275E-2 x + 1.174E-3
R 1.0000
DL 0.003969
BEC 0.02227

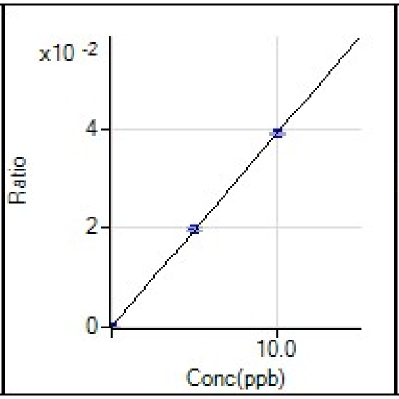

209 Bi [He]
ISTD: 175 Lu
y = 3.911E-3 x + 4.783E-5
R 1.0000
DL 0.007456
BEC 0.01223

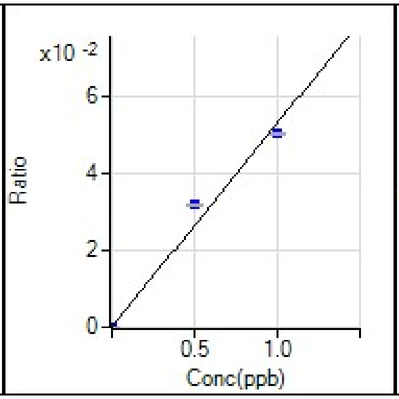

232 Th [He]
ISTD: 175 Lu
y = 5.299E-2 x + 6.187E-5
R 0.9888
DL 0.0008135
BEC 0.001168

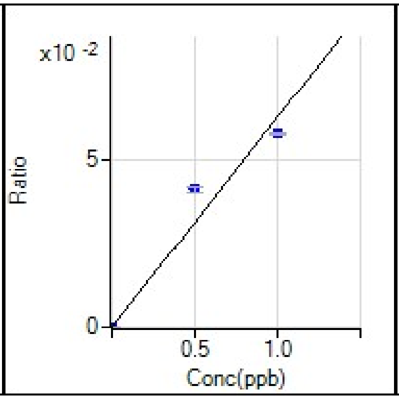

238 U [He]
ISTD: 175 Lu
y = 6.295E-2 x + 4.595E-5
R 0.9715
DL 0.0004065
BEC 0.0007299

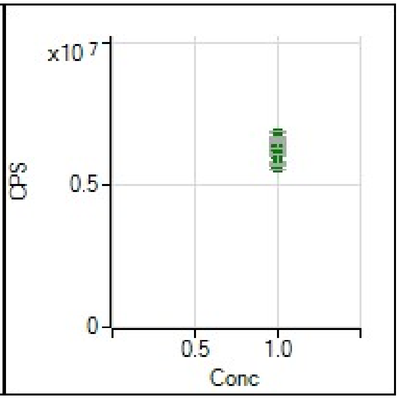

45 Sc [No Gas]
ISTD: ---
Excluded
R
DL
BEC

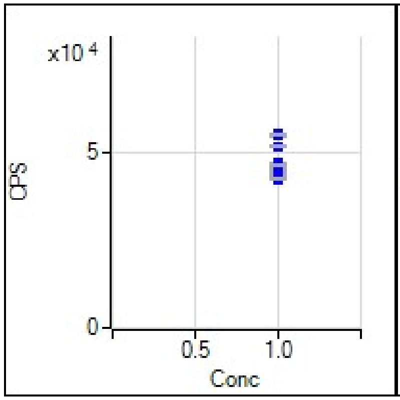

45 Sc [He]
ISTD: ---
Excluded
R
DL
BEC

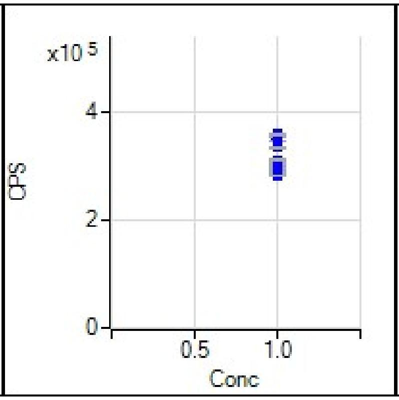

89 Y [He]
ISTD: ---
Excluded
R
DL
BEC

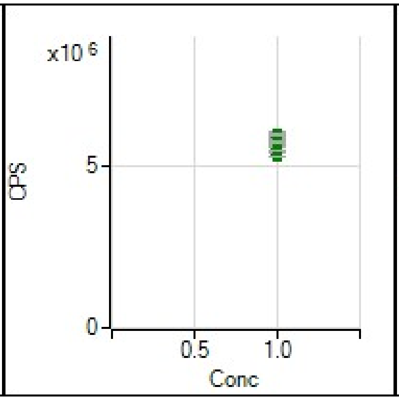

89 -> 105 Y [O2]
ISTD: ---
Excluded
R
DL
BEC

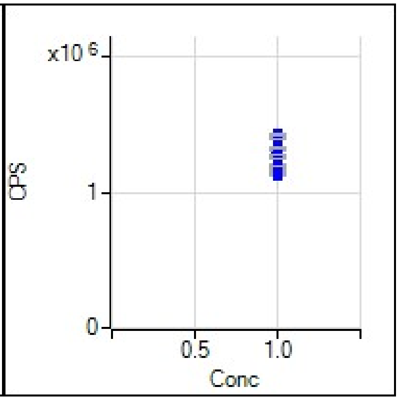

175 Lu [He]
ISTD: ---
Excluded
R
DL
BEC
