# Supplementary figures and images for: Assessment of the Concentration of 51 Elements in the Liver and in Various Parts of the Human Brain—Profiling of the Mineral Status
Source: Nutrients. 2023 Jun 19;15(12):2799. doi: 10.3390/nu15122799 (PMC10302479; doi:10.3390/nu15122799)

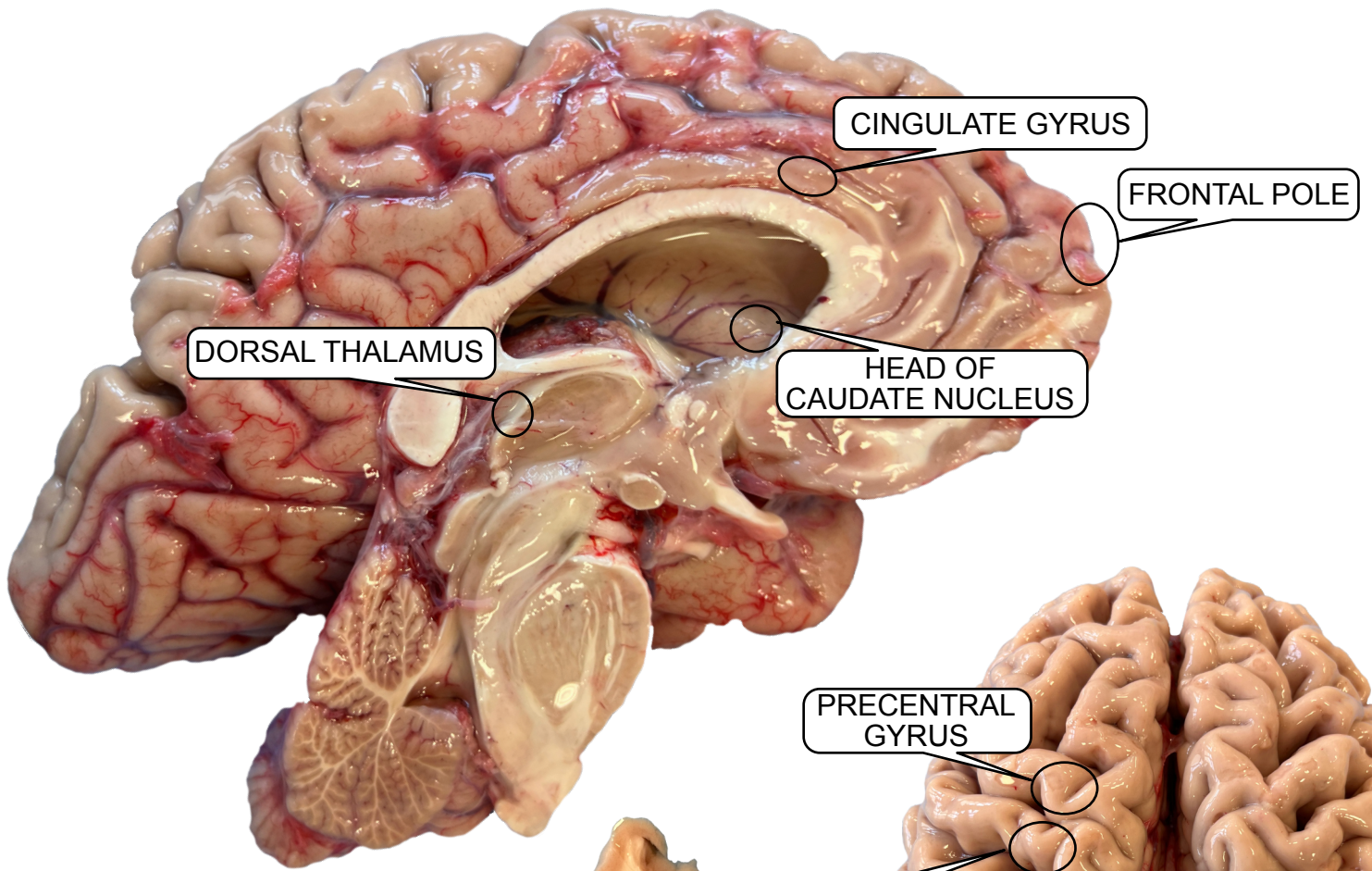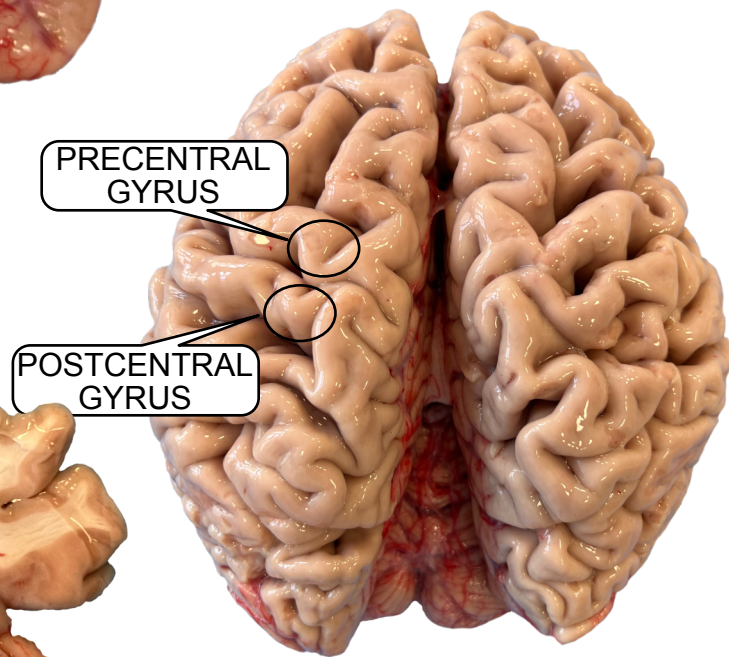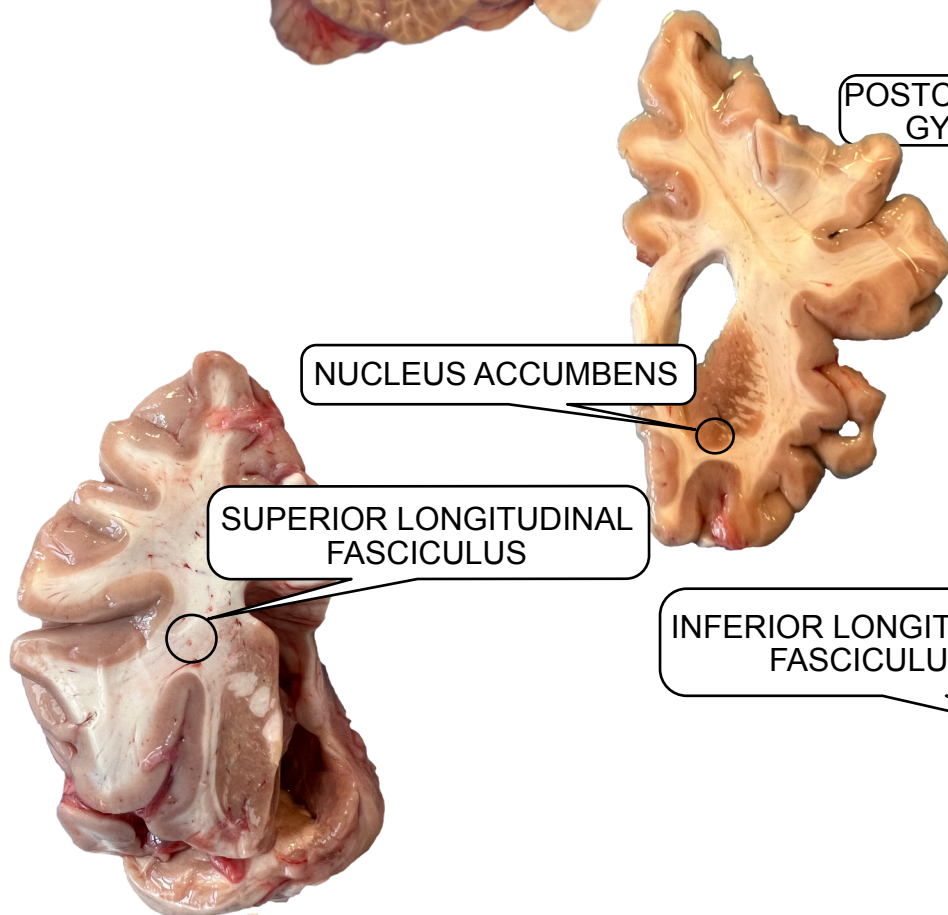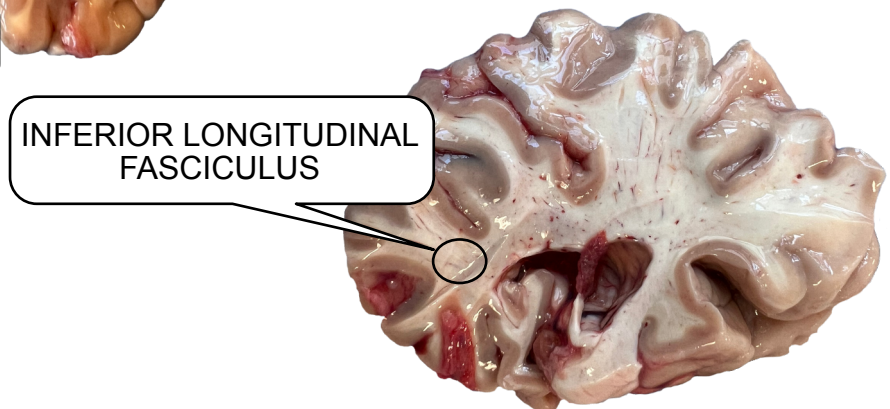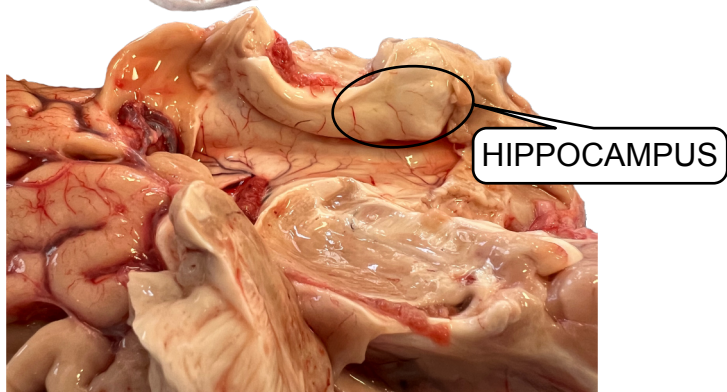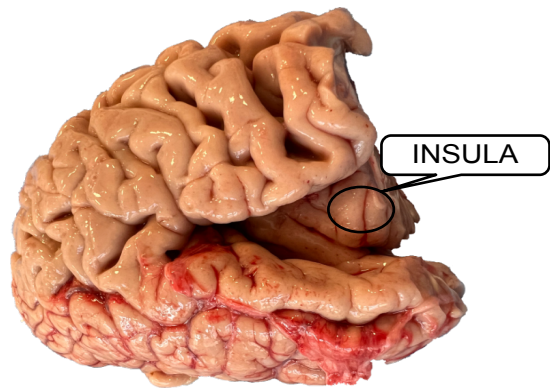

Supplement: Supplementary file 1 [file nutrients-15-02799-s001.zip › Figure S3.pdf]
