# Supplementary material for: Assessment of the Concentration of 51 Elements in the Liver and in Various Parts of the Human Brain—Profiling of the Mineral Status
Source: Nutrients. 2023 Jun 19;15(12):2799. doi: 10.3390/nu15122799 (PMC10302479; doi:10.3390/nu15122799)
Supplement: Supplementary file 1 [file nutrients-15-02799-s001.zip › Figure S2.doc]

**Figure S2.** Comparative graphs for elements significantly differentiated by their content in the examined areas of the brain. Above each graph, the results of Dunn's post-hoc tests, expressed by whiskers connecting significantly different groups, and the p-value with Holm's correction for the multiplicity of comparisons, are placed.
