# Supplementary material for: Assessment of the Concentration of 51 Elements in the Liver and in Various Parts of the Human Brain—Profiling of the Mineral Status
Source: Nutrients. 2023 Jun 19;15(12):2799. doi: 10.3390/nu15122799 (PMC10302479; doi:10.3390/nu15122799)
Supplement: Supplementary file 1 [file nutrients-15-02799-s001.zip › Table S1.doc]

**Table S1A,B** Descriptive statistics covering medians and quartile ranges (Table 1A), mean and standard deviation (Table 1B) for ICP-MS measurements of brain and liver samples taken from the entire population of the studied subjects.

A

|  | **A**  N =16*^1^* | **B**  N = 16*^1^* | **C**  N = 15*^1^* | **D**  N = 15*^1^* | **E**  N = 16*^1^* | **F**  N = 16*^1^* | **G**  N = 16*^1^* | **H**  N = 15*^1^* | **I**  N = 12*^1^* | **J**  N = 16*^1^* | **K**  N = 16*^1^* | **L**  N = 16*^1^* |
| --- | --- | --- | --- | --- | --- | --- | --- | --- | --- | --- | --- | --- |
| Be | 0.00 (0.00, 0.03) | 0.00 (0.00, 0.04) | 0.00 (0.00, 0.00) | 0.00 (0.00, 0.00) | 0.00 (0.00, 0.04) | 0.00 (0.00, 0.06) | 0.00 (0.00, 0.00) | 0.00 (0.00, 0.00) | 0.00 (0.00, 0.02) | 0.00 (0.00, 0.16) | 0.00 (0.00, 0.00) | 0.04 (0.00, 0.15) |
| Na | 1,799 (1,473, 2,051) | 1,788 (1,561, 1,901) | 1,869 (1,598, 1,993) | 1,571 (1,385, 1,747) | 1,519 (1,411, 1,607) | 1,598 (1,350, 1,670) | 1,186 (1,116, 1,257) | 1,215 (1,111, 1,313) | 1,627 (1,483, 1,760) | 1,315 (1,242, 1,494) | 1,556 (1,321, 1,643) | 912 (730, 1,153) |
| Mg | 83 (75, 101) | 90 (86, 96) | 85 (73, 97) | 85 (77, 100) | 90 (82, 101) | 94 (88, 120) | 108 (100, 129) | 122 (96, 130) | 105 (97, 114) | 119 (106, 130) | 92 (88, 102) | 105 (84, 117) |
| Al | 10,109 (6,623, 12,674) | 11,668 (8,390, 15,184) | 8,555 (7,069, 10,914) | 13,907 (8,190, 17,141) | 9,922 (8,282, 12,168) | 9,780 (8,756, 16,886) | 9,685 (7,167, 13,889) | 11,808 (9,706, 15,406) | 14,909 (11,568, 18,879) | 13,950 (10,343, 19,488) | 10,701 (7,405, 14,797) | 10,461 (7,698, 14,844) |
| P | 1,878 (1,727, 2,251) | 2,188 (2,075, 2,232) | 1,878 (1,722, 2,125) | 1,899 (1,776, 2,075) | 1,831 (1,740, 1,953) | 2,153 (1,885, 2,484) | 3,607 (3,310, 3,795) | 3,587 (3,317, 3,661) | 2,573 (2,358, 2,947) | 2,825 (2,558, 3,148) | 1,882 (1,713, 2,277) | 1,726 (1,587, 1,928) |
| K | 2,104 (1,922, 2,255) | 2,091 (1,879, 2,232) | 1,888 (1,713, 2,110) | 2,258 (2,189, 2,474) | 2,151 (1,979, 2,295) | 2,408 (2,123, 2,590) | 2,439 (2,332, 2,536) | 2,267 (2,152, 2,539) | 2,361 (2,091, 2,486) | 2,832 (2,532, 3,059) | 2,463 (2,358, 2,684) | 1,961 (1,621, 2,063) |
| Ca | 143 (111, 165) | 125 (104, 137) | 102 (89, 114) | 111 (103, 131) | 101 (94, 114) | 109 (92, 117) | 102 (87, 111) | 111 (82, 132) | 111 (97, 118) | 117 (99, 139) | 90 (83, 108) | 103 (92, 120) |
| Ti | 61 (42, 72) | 77 (49, 101) | 64 (37, 113) | 70 (51, 106) | 65 (49, 158) | 62 (50, 83) | 85 (51, 98) | 87 (67, 107) | 80 (61, 103) | 93 (60, 111) | 71 (46, 137) | 96 (67, 108) |
| V | 6.02 (5.37, 6.45) | 7.60 (6.24, 8.80) | 4.96 (4.29, 5.33) | 6.42 (5.66, 7.28) | 6.00 (4.73, 6.97) | 5.59 (5.27, 7.75) | 5.57 (4.27, 6.89) | 5.79 (4.69, 7.07) | 6.79 (4.99, 8.25) | 7.31 (5.72, 8.76) | 4.79 (4.06, 6.53) | 9.25 (5.85, 11.78) |
| Cr | 11 (0, 31) | 13 (3, 45) | 29 (5, 83) | 6 (1, 29) | 20 (3, 44) | 11 (3, 32) | 0 (0, 11) | 12 (5, 41) | 3 (0, 10) | 17 (4, 30) | 7 (1, 12) | 11 (0, 42) |
| Mn | 207 (183, 235) | 248 (224, 273) | 213 (192, 245) | 203 (194, 223) | 253 (236, 291) | 462 (407, 502) | 374 (338, 406) | 343 (319, 379) | 364 (337, 386) | 489 (446, 554) | 214 (200, 240) | 1,069 (903, 1,321) |
| Fe | 37,184 (30,834, 42,627) | 56,314 (51,842, 61,893) | 43,884 (38,075, 46,855) | 29,315 (26,442, 31,885) | 31,742 (28,977, 35,451) | 103,223 (98,176, 117,884) | 35,603 (29,674, 46,290) | 39,021 (34,790, 42,088) | 61,354 (49,218, 73,415) | 104,887 (82,107, 131,327) | 33,895 (30,147, 40,635) | 113,997 (83,334, 199,965) |
| Co | 2 (2, 3) | 3 (2, 4) | 3 (2, 5) | 3 (2, 4) | 2 (2, 3) | 3 (3, 6) | 2 (2, 3) | 3 (2, 3) | 4 (3, 5) | 4 (3, 5) | 2 (2, 4) | 35 (24, 40) |
| Ni | 4 (0, 31) | 1 (0, 11) | 1 (0, 12) | 3 (0, 7) | 7 (0, 13) | 0 (0, 5) | 0 (0, 4) | 2 (0, 14) | 3 (1, 5) | 1 (0, 9) | 5 (0, 13) | 4 (0, 14) |
| Cu | 3,619 (3,161, 4,626) | 4,484 (3,796, 5,247) | 3,980 (3,067, 4,598) | 3,665 (3,388, 4,481) | 2,854 (2,163, 3,598) | 5,314 (4,090, 5,962) | 4,038 (3,582, 4,739) | 3,546 (3,078, 4,444) | 3,077 (2,510, 4,486) | 5,609 (5,054, 6,606) | 3,776 (3,304, 4,690) | 3,404 (2,379, 5,038) |
| Zn | 14,358 (11,492, 17,490) | 10,828 (9,489, 13,180) | 11,298 (10,158, 14,056) | 12,897 (11,349, 14,851) | 14,539 (13,746, 16,748) | 12,737 (11,994, 14,115) | 8,549 (7,677, 8,874) | 8,223 (7,819, 9,762) | 11,897 (10,403, 12,896) | 13,586 (11,872, 15,540) | 13,549 (12,907, 15,096) | 41,866 (31,964, 49,886) |
| Ga | 3 (2, 5) | 4 (3, 6) | 4 (2, 19) | 4 (2, 26) | 4 (3, 20) | 4 (2, 14) | 3 (2, 5) | 6 (4, 20) | 8 (6, 23) | 6 (4, 12) | 4 (3, 18) | 5 (3, 24) |
| Rb | 1,290 (1,077, 1,679) | 1,542 (1,374, 1,637) | 1,405 (1,237, 1,565) | 1,591 (1,433, 2,056) | 1,599 (1,528, 1,755) | 1,984 (1,710, 2,366) | 2,059 (1,798, 2,193) | 1,923 (1,670, 2,095) | 1,936 (1,752, 2,398) | 2,423 (2,058, 2,733) | 1,936 (1,748, 2,115) | 2,945 (2,016, 3,106) |
| Sr | 328 (282, 426) | 415 (318, 490) | 275 (255, 350) | 353 (290, 408) | 284 (249, 381) | 350 (267, 438) | 313 (238, 382) | 410 (243, 561) | 300 (270, 441) | 365 (280, 466) | 330 (237, 382) | 418 (347, 526) |
| Zr | 35 (16, 68) | 28 (15, 65) | 32 (12, 100) | 63 (26, 198) | 27 (23, 133) | 42 (25, 114) | 26 (14, 52) | 102 (24, 192) | 78 (15, 170) | 70 (29, 173) | 43 (11, 146) | 58 (13, 128) |
| As | 1.8 (0.4, 6.4) | 1.2 (0.5, 7.7) | 1.7 (0.2, 6.9) | 2.5 (0.4, 8.7) | 1.6 (0.4, 7.1) | 1.7 (0.7, 4.8) | 2.8 (1.1, 10.1) | 4.1 (1.9, 8.1) | 5.1 (2.2, 8.7) | 3.7 (0.5, 8.1) | 1.4 (0.1, 5.7) | 4.3 (2.9, 9.9) |
| Se | 91 (79, 119) | 99 (91, 112) | 93 (79, 109) | 88 (82, 98) | 83 (70, 92) | 105 (103, 130) | 83 (70, 97) | 90 (70, 98) | 130 (113, 147) | 125 (116, 145) | 97 (88, 115) | 188 (165, 211) |
| Mo | 184 (139, 238) | 226 (175, 261) | 229 (195, 266) | 199 (169, 222) | 176 (143, 194) | 575 (464, 668) | 76 (46, 94) | 92 (83, 121) | 235 (226, 257) | 513 (323, 789) | 238 (193, 261) | 3,251 (1,960, 5,480) |
| Pd | 0.00 (0.00, 0.02) | 0.00 (0.00, 0.06) | 0.00 (0.00, 0.00) | 0.00 (0.00, 0.00) | 0.00 (0.00, 0.01) | 0.00 (0.00, 0.00) | 0.00 (0.00, 0.00) | 0.00 (0.00, 0.00) | 0.00 (0.00, 0.00) | 0.00 (0.00, 0.01) | 0.00 (0.00, 0.03) | 0.00 (0.00, 0.01) |
| Ag | 6 (3, 16) | 13 (6, 24) | 6 (3, 7) | 8 (7, 13) | 4 (1, 23) | 8 (5, 38) | 8 (4, 13) | 5 (3, 8) | 6 (3, 14) | 11 (6, 24) | 10 (6, 23) | 5 (4, 18) |
| Cd | 14 (10, 20) | 14 (9, 19) | 16 (9, 21) | 10 (7, 16) | 11 (8, 16) | 18 (14, 28) | 13 (6, 17) | 14 (8, 19) | 17 (12, 28) | 20 (14, 28) | 13 (9, 17) | 536 (360, 1,096) |
| Sn | 13 (5, 37) | 20 (6, 54) | 11 (6, 169) | 18 (7, 391) | 13 (6, 96) | 17 (7, 100) | 6 (5, 16) | 28 (12, 365) | 45 (19, 355) | 41 (11, 350) | 15 (6, 316) | 32 (16, 337) |
| Sb | 1 (0, 2) | 1 (0, 20) | 0 (0, 3) | 0 (0, 1) | 0 (0, 3) | 0 (0, 2) | 0 (0, 1) | 0 (0, 7) | 0 (0, 2) | 1 (0, 2) | 0 (0, 2) | 1 (0, 2) |
| Cs | 3.95 (3.50, 4.76) | 5.02 (3.98, 7.01) | 4.58 (3.59, 5.18) | 6.26 (5.01, 6.84) | 5.93 (4.49, 7.46) | 6.37 (5.21, 7.89) | 7.41 (5.51, 8.76) | 6.83 (5.50, 8.84) | 6.81 (5.83, 8.60) | 8.22 (7.01, 9.97) | 6.23 (5.17, 8.73) | 8.56 (6.73, 13.55) |
| Ba | 43 (35, 217) | 64 (29, 240) | 42 (23, 217) | 47 (35, 311) | 50 (31, 257) | 52 (21, 151) | 42 (30, 300) | 53 (35, 271) | 190 (56, 296) | 92 (33, 268) | 46 (16, 198) | 109 (23, 255) |
| La | 2.44 (2.02, 3.19) | 3.27 (2.20, 4.13) | 2.00 (1.70, 2.39) | 2.81 (2.22, 3.50) | 2.54 (2.09, 3.12) | 2.50 (2.17, 3.42) | 2.38 (1.73, 3.39) | 2.55 (2.05, 3.68) | 3.17 (2.40, 4.18) | 3.71 (3.11, 4.62) | 2.19 (1.71, 2.84) | 9.97 (6.74, 12.69) |
| Ce | 6 (3, 9) | 9 (5, 14) | 7 (3, 217) | 8 (3, 557) | 7 (3, 127) | 9 (5, 44) | 6 (3, 9) | 13 (5, 485) | 17 (10, 487) | 14 (7, 185) | 10 (5, 370) | 21 (15, 256) |
| Pr | 0.24 (0.00, 0.82) | 0.31 (0.00, 0.90) | 0.47 (0.00, 0.62) | 0.10 (0.00, 0.69) | 0.35 (0.00, 0.66) | 0.47 (0.00, 0.67) | 0.19 (0.00, 0.90) | 0.51 (0.00, 0.81) | 0.04 (0.00, 1.25) | 0.71 (0.00, 1.07) | 0.19 (0.00, 0.65) | 0.71 (0.24, 1.37) |
| Nd | 1.89 (1.26, 3.34) | 3.45 (1.88, 3.94) | 1.76 (1.11, 2.48) | 2.40 (1.75, 3.53) | 2.41 (1.87, 2.87) | 2.62 (1.81, 3.03) | 2.11 (1.05, 3.80) | 2.71 (1.53, 3.29) | 2.51 (1.68, 4.75) | 3.48 (2.62, 4.39) | 1.78 (1.28, 2.74) | 3.88 (2.42, 5.71) |
| Sm | 0.10 (0.00, 0.48) | 0.07 (0.00, 0.55) | 0.21 (0.00, 0.35) | 0.00 (0.00, 0.49) | 0.11 (0.00, 0.38) | 0.11 (0.00, 0.31) | 0.09 (0.00, 0.64) | 0.28 (0.00, 0.48) | 0.00 (0.00, 0.82) | 0.41 (0.00, 0.62) | 0.05 (0.00, 0.36) | 0.00 (0.00, 0.41) |
| Eu | 0.03 (0.00, 0.17) | 0.06 (0.00, 0.15) | 0.10 (0.00, 0.13) | 0.00 (0.00, 0.16) | 0.04 (0.00, 0.14) | 0.05 (0.00, 0.14) | 0.04 (0.00, 0.19) | 0.12 (0.00, 0.18) | 0.00 (0.00, 0.27) | 0.07 (0.00, 0.22) | 0.03 (0.00, 0.13) | 0.00 (0.00, 0.13) |
| Gd | 0.50 (0.00, 1.67) | 0.46 (0.00, 4.24) | 0.44 (0.34, 3.97) | 0.78 (0.24, 5.32) | 0.51 (0.18, 3.83) | 0.45 (0.22, 2.09) | 0.43 (0.00, 0.60) | 0.86 (0.49, 4.37) | 2.68 (0.73, 5.76) | 0.71 (0.48, 4.89) | 0.80 (0.25, 3.57) | 0.92 (0.26, 5.30) |
| Tb | 0.014 (0.000, 0.058) | 0.017 (0.000, 0.048) | 0.025 (0.000, 0.038) | 0.000 (0.000, 0.054) | 0.016 (0.000, 0.045) | 0.014 (0.000, 0.041) | 0.012 (0.000, 0.070) | 0.029 (0.000, 0.057) | 0.000 (0.000, 0.086) | 0.021 (0.000, 0.058) | 0.012 (0.000, 0.038) | 0.000 (0.000, 0.050) |
| Dy | 0.06 (0.00, 0.28) | 0.09 (0.00, 0.37) | 0.15 (0.00, 0.25) | 0.00 (0.00, 0.30) | 0.11 (0.00, 0.26) | 0.10 (0.00, 0.25) | 0.09 (0.00, 0.37) | 0.19 (0.00, 0.35) | 0.00 (0.00, 0.45) | 0.11 (0.00, 0.40) | 0.08 (0.00, 0.22) | 0.00 (0.00, 0.27) |
| Ho | 0.00 (0.00, 0.04) | 0.01 (0.00, 0.07) | 0.01 (0.00, 0.05) | 0.00 (0.00, 0.05) | 0.00 (0.00, 0.05) | 0.00 (0.00, 0.04) | 0.00 (0.00, 0.07) | 0.03 (0.00, 0.07) | 0.00 (0.00, 0.03) | 0.01 (0.00, 0.08) | 0.01 (0.00, 0.04) | 0.00 (0.00, 0.05) |
| Er | 0.06 (0.00, 0.31) | 0.01 (0.00, 0.25) | 0.17 (0.00, 0.21) | 0.00 (0.00, 0.41) | 0.13 (0.00, 0.32) | 0.11 (0.00, 0.35) | 0.11 (0.00, 0.34) | 0.22 (0.00, 0.36) | 0.10 (0.00, 0.37) | 0.05 (0.00, 0.30) | 0.06 (0.00, 0.30) | 0.05 (0.00, 0.36) |
| Tm | 0.007 (0.000, 0.023) | 0.013 (0.000, 0.039) | 0.008 (0.000, 0.023) | 0.000 (0.000, 0.028) | 0.003 (0.000, 0.023) | 0.008 (0.000, 0.028) | 0.006 (0.000, 0.037) | 0.008 (0.000, 0.028) | 0.000 (0.000, 0.029) | 0.008 (0.000, 0.033) | 0.005 (0.000, 0.025) | 0.000 (0.000, 0.025) |
| Yb | 0.07 (0.00, 0.23) | 0.08 (0.00, 0.30) | 0.13 (0.00, 0.21) | 0.00 (0.00, 0.27) | 0.10 (0.00, 0.24) | 0.06 (0.00, 0.26) | 0.08 (0.00, 0.25) | 0.18 (0.00, 0.27) | 0.00 (0.00, 0.32) | 0.10 (0.00, 0.29) | 0.07 (0.00, 0.24) | 0.00 (0.00, 0.30) |
| Hf | 1.54 (0.64, 2.77) | 1.75 (0.65, 4.01) | 1.94 (1.23, 3.27) | 2.17 (1.29, 4.77) | 1.48 (0.95, 3.41) | 1.54 (1.07, 2.86) | 1.38 (0.37, 1.76) | 4.23 (1.60, 4.71) | 2.08 (0.86, 4.56) | 2.25 (1.58, 3.75) | 2.83 (0.83, 4.29) | 1.91 (0.75, 3.83) |
| Pt | 1.79 (1.35, 2.46) | 2.13 (1.49, 2.74) | 1.59 (0.89, 2.58) | 2.11 (1.15, 3.85) | 1.69 (1.30, 2.23) | 1.78 (1.60, 2.07) | 1.94 (1.69, 2.32) | 2.37 (1.68, 3.43) | 2.22 (1.57, 3.02) | 2.37 (1.73, 3.25) | 2.06 (1.41, 2.43) | 1.95 (1.58, 3.13) |
| Hg | 8 (2, 21) | 9 (3, 16) | 16 (5, 19) | 10 (0, 18) | 5 (2, 11) | 8 (0, 17) | 7 (1, 12) | 10 (1, 16) | 6 (2, 12) | 7 (3, 23) | 8 (1, 10) | 14 (9, 29) |
| Hg2 | 7 (2, 21) | 9 (3, 16) | 16 (5, 19) | 10 (0, 18) | 5 (2, 11) | 8 (0, 17) | 6 (1, 12) | 10 (1, 16) | 6 (2, 12) | 6 (3, 23) | 8 (2, 11) | 14 (9, 29) |
| Tl | 0.00 (0.00, 0.19) | 0.00 (0.00, 0.11) | 0.00 (0.00, 0.12) | 0.00 (0.00, 0.09) | 0.00 (0.00, 0.10) | 0.00 (0.00, 0.26) | 0.00 (0.00, 0.11) | 0.00 (0.00, 0.06) | 0.00 (0.00, 0.03) | 0.00 (0.00, 0.12) | 0.00 (0.00, 0.11) | 0.05 (0.00, 0.20) |
| Pb | 18 (9, 24) | 19 (11, 27) | 17 (11, 31) | 25 (17, 32) | 14 (10, 22) | 10 (6, 30) | 10 (6, 24) | 22 (5, 29) | 21 (15, 28) | 22 (13, 35) | 18 (8, 28) | 58 (44, 98) |
| Bi | 3 (2, 7) | 3 (1, 5) | 2 (1, 3) | 3 (2, 6) | 2 (1, 3) | 3 (2, 8) | 2 (1, 4) | 2 (1, 3) | 2 (2, 4) | 5 (2, 7) | 2 (2, 5) | 3 (1, 6) |
| Th | 0.02 (0.00, 0.41) | 0.08 (0.00, 0.29) | 0.00 (0.00, 0.22) | 0.00 (0.00, 0.42) | 0.08 (0.00, 0.29) | 0.07 (0.00, 0.23) | 0.07 (0.00, 0.32) | 0.00 (0.00, 0.72) | 0.03 (0.00, 0.74) | 0.15 (0.00, 0.44) | 0.00 (0.00, 0.20) | 0.00 (0.00, 0.39) |
| U | 0.19 (0.02, 0.50) | 0.26 (0.00, 0.50) | 0.27 (0.00, 0.40) | 0.21 (0.04, 0.59) | 0.29 (0.05, 0.46) | 0.33 (0.03, 0.44) | 0.27 (0.00, 0.55) | 0.32 (0.02, 0.56) | 0.13 (0.00, 0.72) | 0.42 (0.09, 0.59) | 0.13 (0.00, 0.37) | 0.28 (0.01, 0.51) |
| *^1^* Median (IQR) | | | | | | | | | | | | |

**B**

|  | **A**  N = 16*^1^* | **B**  N = 16*^1^* | **C**  N = 15*^1^* | **D**  N = 15*^1^* | **E**  N =16*^1^* | **F**  N = 16*^1^* | **G**  N =16*^1^* | **H**  N = 15*^1^* | **I**  N = 12*^1^* | **J**  N = 16*^1^* | **K**  N =16*^1^* | **L**  N = 16*^1^* |
| --- | --- | --- | --- | --- | --- | --- | --- | --- | --- | --- | --- | --- |
| Be | 0.02 (0.04) | 0.04 (0.06) | 0.02 (0.06) | 0.02 (0.04) | 0.05 (0.12) | 0.05 (0.09) | 0.05 (0.15) | 0.01 (0.03) | 0.05 (0.11) | 0.14 (0.27) | 0.06 (0.19) | 0.13 (0.21) |
| Na | 1,835 (465) | 1,764 (321) | 1,715 (472) | 1,566 (277) | 1,487 (184) | 1,550 (248) | 1,188 (120) | 1,241 (158) | 1,605 (259) | 1,375 (235) | 1,492 (208) | 931 (253) |
| Mg | 91 (24) | 98 (32) | 85 (22) | 88 (14) | 92 (16) | 102 (21) | 112 (16) | 114 (18) | 105 (16) | 120 (18) | 93 (12) | 102 (29) |
| Al | 10,075 (3,693) | 12,079 (5,043) | 9,484 (3,950) | 13,846 (6,255) | 11,628 (6,041) | 12,369 (5,959) | 10,203 (4,196) | 12,798 (4,235) | 15,984 (6,085) | 16,093 (7,751) | 12,895 (9,337) | 12,536 (7,269) |
| P | 1,982 (492) | 2,230 (462) | 1,935 (532) | 1,880 (241) | 1,887 (299) | 2,181 (378) | 3,542 (391) | 3,541 (449) | 2,625 (412) | 2,928 (529) | 1,997 (307) | 1,819 (508) |
| K | 2,146 (578) | 2,151 (445) | 1,881 (460) | 2,244 (375) | 2,168 (354) | 2,345 (376) | 2,402 (213) | 2,365 (295) | 2,328 (401) | 2,845 (486) | 2,528 (306) | 1,854 (415) |
| Ca | 141 (37) | 123 (27) | 99 (23) | 119 (24) | 113 (37) | 109 (24) | 101 (19) | 110 (31) | 113 (26) | 122 (29) | 103 (46) | 104 (25) |
| Ti | 142 (303) | 157 (320) | 76 (47) | 97 (83) | 127 (164) | 82 (62) | 86 (44) | 99 (58) | 81 (28) | 106 (67) | 107 (100) | 85 (32) |
| V | 6.04 (1.35) | 7.45 (1.99) | 5.30 (1.89) | 6.59 (1.54) | 7.34 (5.83) | 6.76 (2.83) | 5.53 (2.09) | 6.04 (2.35) | 7.00 (2.61) | 7.79 (3.39) | 5.91 (4.04) | 10.36 (5.93) |
| Cr | 29 (60) | 27 (31) | 124 (276) | 19 (24) | 428 (1,582) | 75 (237) | 10 (23) | 64 (116) | 7 (9) | 44 (100) | 9 (11) | 50 (113) |
| Mn | 214 (44) | 398 (616) | 230 (61) | 215 (34) | 275 (63) | 472 (123) | 397 (137) | 380 (140) | 360 (36) | 501 (120) | 219 (35) | 1,144 (363) |
| Fe | 39,218 (11,447) | 59,162 (16,266) | 44,138 (11,333) | 29,117 (4,944) | 34,031 (9,477) | 107,254 (21,887) | 37,214 (9,082) | 40,152 (10,919) | 61,276 (16,656) | 104,815 (37,467) | 35,364 (8,104) | 137,786 (90,228) |
| Co | 3 (2) | 9 (23) | 7 (12) | 3 (2) | 4 (3) | 5 (5) | 2 (1) | 3 (1) | 4 (2) | 4 (2) | 3 (2) | 35 (16) |
| Ni | 26 (60) | 172 (659) | 10 (17) | 141 (504) | 20 (39) | 8 (18) | 5 (13) | 129 (423) | 26 (79) | 63 (171) | 9 (12) | 15 (34) |
| Cu | 3,980 (1,445) | 4,799 (1,678) | 3,868 (1,220) | 3,939 (823) | 2,996 (855) | 5,192 (1,187) | 4,040 (931) | 3,832 (1,106) | 3,508 (1,326) | 5,628 (1,174) | 3,881 (842) | 3,836 (1,738) |
| Zn | 14,320 (3,577) | 17,842 (26,354) | 12,104 (2,461) | 19,642 (25,964) | 15,648 (2,943) | 13,618 (2,663) | 8,268 (1,282) | 13,642 (17,019) | 11,581 (2,059) | 15,487 (5,980) | 14,201 (2,312) | 43,374 (18,895) |
| Ga | 7 (8) | 8 (11) | 15 (25) | 13 (14) | 12 (15) | 14 (24) | 6 (8) | 12 (10) | 15 (13) | 11 (11) | 11 (11) | 18 (31) |
| Rb | 1,423 (473) | 1,859 (1,377) | 1,419 (333) | 1,681 (433) | 1,725 (401) | 2,089 (482) | 2,040 (365) | 2,018 (409) | 2,051 (396) | 2,399 (468) | 1,936 (280) | 2,764 (1,026) |
| Sr | 343 (107) | 392 (124) | 292 (92) | 354 (115) | 299 (96) | 380 (146) | 313 (80) | 437 (262) | 366 (163) | 427 (252) | 345 (185) | 468 (197) |
| Zr | 64 (74) | 62 (80) | 71 (88) | 122 (117) | 82 (99) | 89 (101) | 53 (71) | 110 (89) | 108 (107) | 99 (94) | 85 (91) | 83 (83) |
| As | 3.2 (3.5) | 4.1 (4.9) | 4.4 (5.0) | 5.0 (5.8) | 4.5 (5.3) | 3.2 (3.6) | 8.1 (12.4) | 5.0 (3.6) | 5.8 (4.5) | 8.1 (15.2) | 3.0 (3.4) | 7.9 (7.8) |
| Se | 110 (59) | 100 (43) | 90 (32) | 96 (29) | 92 (45) | 128 (57) | 88 (36) | 93 (41) | 135 (51) | 138 (44) | 107 (39) | 236 (191) |
| Mo | 200 (79) | 1,384 (4,683) | 277 (154) | 193 (42) | 176 (70) | 593 (200) | 75 (36) | 96 (43) | 234 (66) | 544 (276) | 230 (53) | 4,291 (3,378) |
| Pd | 0.49 (1.92) | 0.22 (0.77) | 0.11 (0.40) | 0.01 (0.02) | 0.04 (0.14) | 0.03 (0.09) | 0.03 (0.10) | 0.00 (0.01) | 0.04 (0.13) | 0.11 (0.40) | 0.14 (0.52) | 0.05 (0.16) |
| Ag | 12 (13) | 16 (13) | 12 (19) | 16 (24) | 49 (152) | 49 (86) | 12 (13) | 30 (83) | 13 (15) | 28 (47) | 15 (16) | 49 (137) |
| Cd | 20 (21) | 159 (566) | 42 (92) | 13 (9) | 16 (17) | 26 (30) | 18 (23) | 17 (20) | 30 (41) | 28 (33) | 18 (20) | 800 (682) |
| Sn | 81 (148) | 98 (172) | 116 (189) | 177 (250) | 128 (230) | 128 (212) | 59 (142) | 155 (205) | 202 (237) | 166 (209) | 139 (202) | 148 (199) |
| Sb | 13 (48) | 22 (39) | 17 (50) | 3 (11) | 11 (31) | 4 (12) | 2 (7) | 11 (25) | 7 (22) | 3 (5) | 9 (24) | 2 (3) |
| Cs | 4.23 (1.24) | 5.80 (2.47) | 4.69 (1.63) | 5.78 (1.70) | 6.01 (2.26) | 6.88 (2.14) | 7.67 (3.13) | 7.60 (2.85) | 7.67 (2.75) | 8.53 (2.69) | 6.73 (2.12) | 9.93 (4.29) |
| Ba | 124 (147) | 144 (155) | 156 (191) | 166 (177) | 163 (175) | 123 (151) | 212 (297) | 153 (143) | 201 (162) | 263 (479) | 102 (106) | 172 (183) |
| La | 2.54 (0.77) | 3.86 (3.39) | 2.37 (1.37) | 2.98 (1.11) | 2.74 (1.17) | 3.06 (1.41) | 2.51 (1.06) | 2.94 (1.11) | 3.49 (1.67) | 4.23 (2.17) | 2.83 (2.42) | 12.72 (14.13) |
| Ce | 103 (212) | 123 (251) | 152 (261) | 238 (348) | 170 (318) | 148 (287) | 75 (195) | 201 (287) | 262 (329) | 173 (286) | 182 (281) | 183 (281) |
| Pr | 0.39 (0.42) | 0.50 (0.59) | 0.36 (0.36) | 0.38 (0.46) | 0.40 (0.43) | 0.44 (0.49) | 0.41 (0.47) | 0.48 (0.50) | 0.58 (0.76) | 0.68 (0.63) | 0.48 (0.81) | 0.89 (0.79) |
| Nd | 2.24 (1.32) | 2.98 (1.47) | 1.87 (1.13) | 2.73 (1.51) | 2.45 (1.31) | 2.78 (1.58) | 2.31 (1.57) | 2.72 (1.64) | 3.34 (2.44) | 3.93 (2.46) | 2.68 (3.23) | 4.03 (1.96) |
| Sm | 0.24 (0.29) | 0.25 (0.29) | 0.21 (0.23) | 0.24 (0.32) | 0.23 (0.28) | 0.21 (0.29) | 0.26 (0.32) | 0.29 (0.34) | 0.40 (0.57) | 0.39 (0.40) | 0.30 (0.61) | 0.25 (0.38) |
| Eu | 0.08 (0.09) | 0.09 (0.10) | 0.08 (0.09) | 0.08 (0.10) | 0.08 (0.09) | 0.08 (0.09) | 0.10 (0.12) | 0.10 (0.10) | 0.13 (0.17) | 0.13 (0.17) | 0.10 (0.17) | 0.08 (0.11) |
| Gd | 1.43 (2.05) | 28.51 (107.43) | 1.86 (2.25) | 2.57 (3.07) | 1.95 (2.71) | 2.45 (4.17) | 1.31 (2.35) | 2.28 (2.44) | 3.24 (2.96) | 2.56 (3.44) | 2.14 (2.45) | 21.82 (78.83) |
| Tb | 0.029 (0.033) | 0.028 (0.031) | 0.024 (0.028) | 0.028 (0.037) | 0.026 (0.031) | 0.029 (0.040) | 0.030 (0.036) | 0.033 (0.038) | 0.041 (0.057) | 0.039 (0.050) | 0.035 (0.064) | 0.027 (0.037) |
| Dy | 0.15 (0.18) | 0.18 (0.20) | 0.14 (0.16) | 0.15 (0.19) | 0.16 (0.19) | 0.16 (0.20) | 0.18 (0.20) | 0.20 (0.24) | 0.22 (0.31) | 0.21 (0.23) | 0.19 (0.31) | 0.15 (0.20) |
| Ho | 0.03 (0.05) | 0.04 (0.05) | 0.03 (0.04) | 0.03 (0.05) | 0.02 (0.03) | 0.02 (0.03) | 0.04 (0.06) | 0.05 (0.06) | 0.03 (0.06) | 0.04 (0.06) | 0.04 (0.06) | 0.02 (0.03) |
| Er | 0.16 (0.19) | 0.16 (0.22) | 0.13 (0.14) | 0.22 (0.29) | 0.16 (0.18) | 0.43 (1.05) | 0.19 (0.22) | 0.42 (0.87) | 0.36 (0.73) | 0.17 (0.20) | 0.19 (0.27) | 0.21 (0.28) |
| Tm | 0.014 (0.018) | 0.021 (0.023) | 0.017 (0.021) | 0.014 (0.017) | 0.014 (0.018) | 0.016 (0.019) | 0.019 (0.022) | 0.019 (0.025) | 0.015 (0.019) | 0.023 (0.031) | 0.018 (0.027) | 0.015 (0.021) |
| Yb | 0.11 (0.12) | 0.21 (0.35) | 0.12 (0.13) | 0.12 (0.15) | 0.13 (0.15) | 0.13 (0.16) | 0.16 (0.18) | 0.15 (0.16) | 0.15 (0.18) | 0.15 (0.17) | 0.14 (0.18) | 0.17 (0.27) |
| Hf | 2.03 (1.93) | 4.15 (8.54) | 3.59 (5.65) | 3.05 (2.36) | 2.21 (2.00) | 2.54 (2.51) | 1.61 (1.58) | 3.36 (2.03) | 2.80 (2.36) | 3.12 (2.85) | 2.66 (2.06) | 2.19 (1.74) |
| Pt | 2.19 (1.69) | 2.20 (0.90) | 1.94 (1.23) | 2.36 (1.40) | 2.07 (1.31) | 2.14 (1.14) | 2.08 (0.73) | 2.59 (1.07) | 2.46 (1.34) | 2.50 (1.05) | 2.09 (1.04) | 2.34 (1.25) |
| Hg | 12 (13) | 12 (12) | 14 (10) | 11 (12) | 7 (6) | 10 (10) | 8 (8) | 11 (11) | 8 (9) | 13 (14) | 8 (9) | 18 (14) |
| Hg2 | 12 (13) | 12 (12) | 14 (9) | 11 (11) | 7 (6) | 10 (10) | 8 (8) | 11 (11) | 8 (9) | 13 (14) | 8 (9) | 18 (14) |
| Tl | 0.30 (0.58) | 0.28 (0.70) | 5.89 (22.32) | 0.06 (0.11) | 1.89 (6.66) | 3.98 (11.69) | 1.89 (6.73) | 0.05 (0.09) | 0.03 (0.06) | 6.76 (26.71) | 0.08 (0.15) | 0.12 (0.15) |
| Pb | 26 (32) | 24 (21) | 23 (21) | 37 (52) | 23 (29) | 23 (25) | 15 (12) | 21 (17) | 21 (12) | 48 (100) | 25 (27) | 84 (85) |
| Bi | 6 (7) | 4 (4) | 7 (17) | 48 (173) | 42 (153) | 15 (29) | 5 (9) | 188 (717) | 5 (9) | 7 (8) | 3 (2) | 389 (1,540) |
| Th | 0.23 (0.31) | 0.18 (0.24) | 0.13 (0.21) | 0.25 (0.40) | 0.26 (0.41) | 0.20 (0.31) | 0.21 (0.30) | 0.30 (0.40) | 0.44 (0.62) | 0.48 (0.88) | 0.41 (0.91) | 0.21 (0.31) |
| U | 0.53 (0.88) | 0.31 (0.35) | 0.22 (0.22) | 0.38 (0.44) | 0.29 (0.24) | 0.38 (0.47) | 0.29 (0.28) | 0.39 (0.50) | 0.61 (1.06) | 0.65 (1.05) | 0.27 (0.41) | 0.44 (0.65) |
| *^1^* Mean (SD) | | | | | | | | | | | | |
