# Supplementary material for: Assessment of the Concentration of 51 Elements in the Liver and in Various Parts of the Human Brain—Profiling of the Mineral Status
Source: Nutrients. 2023 Jun 19;15(12):2799. doi: 10.3390/nu15122799 (PMC10302479; doi:10.3390/nu15122799)
Supplement: Supplementary file 1 [file nutrients-15-02799-s001.zip › Table S2.doc]

**Table S2**. Kruskal-Wallis test to test the hypothesis that the concentrations of individual elements are equal in different brain areas. Elements showing significant differences between brain areas are shown in grey.

|  | **A**, N = 16*^1^* | **B**, N = 16*^1^* | **C**, N = 15*^1^* | **D**, N = 15*^1^* | **E**, N = 16*^1^* | **F**, N = 16*^1^* | **G**, N = 16*^1^* | **H**, N = 15*^1^* | **I**, N = 12*^1^* | **J**, N = 16*^1^* | **K**, N = 16*^1^* | **p-value***^2^* |
| --- | --- | --- | --- | --- | --- | --- | --- | --- | --- | --- | --- | --- |
| Be | 0.00 (0.00, 0.03) | 0.00 (0.00, 0.04) | 0.00 (0.00, 0.00) | 0.00 (0.00, 0.00) | 0.00 (0.00, 0.04) | 0.00 (0.00, 0.06) | 0.00 (0.00, 0.00) | 0.00 (0.00, 0.00) | 0.00 (0.00, 0.02) | 0.00 (0.00, 0.16) | 0.00 (0.00, 0.00) | 0.6 |
| Na | 1,799 (1,473, 2,051) | 1,788 (1,561, 1,901) | 1,869 (1,598, 1,993) | 1,571 (1,385, 1,747) | 1,519 (1,411, 1,607) | 1,598 (1,350, 1,670) | 1,186 (1,116, 1,257) | 1,215 (1,111, 1,313) | 1,627 (1,483, 1,760) | 1,315 (1,242, 1,494) | 1,556 (1,321, 1,643) | <0.001 |
| Mg | 83 (75, 101) | 90 (86, 96) | 85 (73, 97) | 85 (77, 100) | 90 (82, 101) | 94 (88, 120) | 108 (100, 129) | 122 (96, 130) | 105 (97, 114) | 119 (106, 130) | 92 (88, 102) | <0.001 |
| Al | 10,109 (6,623, 12,674) | 11,668 (8,390, 15,184) | 8,555 (7,069, 10,914) | 13,907 (8,190, 17,141) | 9,922 (8,282, 12,168) | 9,780 (8,756, 16,886) | 9,685 (7,167, 13,889) | 11,808 (9,706, 15,406) | 14,909 (11,568, 18,879) | 13,950 (10,343, 19,488) | 10,701 (7,405, 14,797) | 0.025 |
| P | 1,878 (1,727, 2,251) | 2,188 (2,075, 2,232) | 1,878 (1,722, 2,125) | 1,899 (1,776, 2,075) | 1,831 (1,740, 1,953) | 2,153 (1,885, 2,484) | 3,607 (3,310, 3,795) | 3,587 (3,317, 3,661) | 2,573 (2,358, 2,947) | 2,825 (2,558, 3,148) | 1,882 (1,713, 2,277) | <0.001 |
| K | 2,104 (1,922, 2,255) | 2,091 (1,879, 2,232) | 1,888 (1,713, 2,110) | 2,258 (2,189, 2,474) | 2,151 (1,979, 2,295) | 2,408 (2,123, 2,590) | 2,439 (2,332, 2,536) | 2,267 (2,152, 2,539) | 2,361 (2,091, 2,486) | 2,832 (2,532, 3,059) | 2,463 (2,358, 2,684) | <0.001 |
| Ca | 143 (111, 165) | 125 (104, 137) | 102 (89, 114) | 111 (103, 131) | 101 (94, 114) | 109 (92, 117) | 102 (87, 111) | 111 (82, 132) | 111 (97, 118) | 117 (99, 139) | 90 (83, 108) | 0.002 |
| Ti | 61 (42, 72) | 77 (49, 101) | 64 (37, 113) | 70 (51, 106) | 65 (49, 158) | 62 (50, 83) | 85 (51, 98) | 87 (67, 107) | 80 (61, 103) | 93 (60, 111) | 71 (46, 137) | 0.7 |
| V | 6.02 (5.37, 6.45) | 7.60 (6.24, 8.80) | 4.96 (4.29, 5.33) | 6.42 (5.66, 7.28) | 6.00 (4.73, 6.97) | 5.59 (5.27, 7.75) | 5.57 (4.27, 6.89) | 5.79 (4.69, 7.07) | 6.79 (4.99, 8.25) | 7.31 (5.72, 8.76) | 4.79 (4.06, 6.53) | 0.022 |
| Cr | 11 (0, 31) | 13 (3, 45) | 29 (5, 83) | 6 (1, 29) | 20 (3, 44) | 11 (3, 32) | 0 (0, 11) | 12 (5, 41) | 3 (0, 10) | 17 (4, 30) | 7 (1, 12) | 0.12 |
| Mn | 207 (183, 235) | 248 (224, 273) | 213 (192, 245) | 203 (194, 223) | 253 (236, 291) | 462 (407, 502) | 374 (338, 406) | 343 (319, 379) | 364 (337, 386) | 489 (446, 554) | 214 (200, 240) | <0.001 |
| Fe | 37,184 (30,834, 42,627) | 56,314 (51,842, 61,893) | 43,884 (38,075, 46,855) | 29,315 (26,442, 31,885) | 31,742 (28,977, 35,451) | 103,223 (98,176, 117,884) | 35,603 (29,674, 46,290) | 39,021 (34,790, 42,088) | 61,354 (49,218, 73,415) | 104,887 (82,107, 131,327) | 33,895 (30,147, 40,635) | <0.001 |
| Co | 2.37 (2.21, 2.97) | 2.81 (2.38, 3.70) | 2.73 (2.09, 5.08) | 2.73 (2.13, 3.85) | 2.33 (2.04, 3.40) | 3.46 (2.96, 6.23) | 2.22 (2.12, 2.72) | 2.66 (1.85, 3.19) | 3.54 (3.13, 4.51) | 3.86 (3.02, 5.46) | 2.50 (2.37, 3.55) | 0.001 |
| Ni | 4 (0, 31) | 1 (0, 11) | 1 (0, 12) | 3 (0, 7) | 7 (0, 13) | 0 (0, 5) | 0 (0, 4) | 2 (0, 14) | 3 (1, 5) | 1 (0, 9) | 5 (0, 13) | >0.9 |
| Cu | 3,619 (3,161, 4,626) | 4,484 (3,796, 5,247) | 3,980 (3,067, 4,598) | 3,665 (3,388, 4,481) | 2,854 (2,163, 3,598) | 5,314 (4,090, 5,962) | 4,038 (3,582, 4,739) | 3,546 (3,078, 4,444) | 3,077 (2,510, 4,486) | 5,609 (5,054, 6,606) | 3,776 (3,304, 4,690) | <0.001 |
| Zn | 14,358 (11,492, 17,490) | 10,828 (9,489, 13,180) | 11,298 (10,158, 14,056) | 12,897 (11,349, 14,851) | 14,539 (13,746, 16,748) | 12,737 (11,994, 14,115) | 8,549 (7,677, 8,874) | 8,223 (7,819, 9,762) | 11,897 (10,403, 12,896) | 13,586 (11,872, 15,540) | 13,549 (12,907, 15,096) | <0.001 |
| Ga | 3 (2, 5) | 4 (3, 6) | 4 (2, 19) | 4 (2, 26) | 4 (3, 20) | 4 (2, 14) | 3 (2, 5) | 6 (4, 20) | 8 (6, 23) | 6 (4, 12) | 4 (3, 18) | 0.10 |
| Rb | 1,290 (1,077, 1,679) | 1,542 (1,374, 1,637) | 1,405 (1,237, 1,565) | 1,591 (1,433, 2,056) | 1,599 (1,528, 1,755) | 1,984 (1,710, 2,366) | 2,059 (1,798, 2,193) | 1,923 (1,670, 2,095) | 1,936 (1,752, 2,398) | 2,423 (2,058, 2,733) | 1,936 (1,748, 2,115) | <0.001 |
| Sr | 328 (282, 426) | 415 (318, 490) | 275 (255, 350) | 353 (290, 408) | 284 (249, 381) | 350 (267, 438) | 313 (238, 382) | 410 (243, 561) | 300 (270, 441) | 365 (280, 466) | 330 (237, 382) | 0.4 |
| Zr | 35 (16, 68) | 28 (15, 65) | 32 (12, 100) | 63 (26, 198) | 27 (23, 133) | 42 (25, 114) | 26 (14, 52) | 102 (24, 192) | 78 (15, 170) | 70 (29, 173) | 43 (11, 146) | 0.6 |
| As | 1.8 (0.4, 6.4) | 1.2 (0.5, 7.7) | 1.7 (0.2, 6.9) | 2.5 (0.4, 8.7) | 1.6 (0.4, 7.1) | 1.7 (0.7, 4.8) | 2.8 (1.1, 10.1) | 4.1 (1.9, 8.1) | 5.1 (2.2, 8.7) | 3.7 (0.5, 8.1) | 1.4 (0.1, 5.7) | 0.5 |
| Se | 91 (79, 119) | 99 (91, 112) | 93 (79, 109) | 88 (82, 98) | 83 (70, 92) | 105 (103, 130) | 83 (70, 97) | 90 (70, 98) | 130 (113, 147) | 125 (116, 145) | 97 (88, 115) | <0.001 |
| Mo | 184 (139, 238) | 226 (175, 261) | 229 (195, 266) | 199 (169, 222) | 176 (143, 194) | 575 (464, 668) | 76 (46, 94) | 92 (83, 121) | 235 (226, 257) | 513 (323, 789) | 238 (193, 261) | <0.001 |
| Pd | 0.00 (0.00, 0.02) | 0.00 (0.00, 0.06) | 0.00 (0.00, 0.00) | 0.00 (0.00, 0.00) | 0.00 (0.00, 0.01) | 0.00 (0.00, 0.00) | 0.00 (0.00, 0.00) | 0.00 (0.00, 0.00) | 0.00 (0.00, 0.00) | 0.00 (0.00, 0.01) | 0.00 (0.00, 0.03) | 0.4 |
| Ag | 6 (3, 16) | 13 (6, 24) | 6 (3, 7) | 8 (7, 13) | 4 (1, 23) | 8 (5, 38) | 8 (4, 13) | 5 (3, 8) | 6 (3, 14) | 11 (6, 24) | 10 (6, 23) | 0.5 |
| Cd | 14 (10, 20) | 14 (9, 19) | 16 (9, 21) | 10 (7, 16) | 11 (8, 16) | 18 (14, 28) | 13 (6, 17) | 14 (8, 19) | 17 (12, 28) | 20 (14, 28) | 13 (9, 17) | 0.3 |
| Sn | 13 (5, 37) | 20 (6, 54) | 11 (6, 169) | 18 (7, 391) | 13 (6, 96) | 17 (7, 100) | 6 (5, 16) | 28 (12, 365) | 45 (19, 355) | 41 (11, 350) | 15 (6, 316) | 0.3 |
| Sb | 1 (0, 2) | 1 (0, 20) | 0 (0, 3) | 0 (0, 1) | 0 (0, 3) | 0 (0, 2) | 0 (0, 1) | 0 (0, 7) | 0 (0, 2) | 1 (0, 2) | 0 (0, 2) | >0.9 |
| Cs | 3.95 (3.50, 4.76) | 5.02 (3.98, 7.01) | 4.58 (3.59, 5.18) | 6.26 (5.01, 6.84) | 5.93 (4.49, 7.46) | 6.37 (5.21, 7.89) | 7.41 (5.51, 8.76) | 6.83 (5.50, 8.84) | 6.81 (5.83, 8.60) | 8.22 (7.01, 9.97) | 6.23 (5.17, 8.73) | <0.001 |
| Ba | 43 (35, 217) | 64 (29, 240) | 42 (23, 217) | 47 (35, 311) | 50 (31, 257) | 52 (21, 151) | 42 (30, 300) | 53 (35, 271) | 190 (56, 296) | 92 (33, 268) | 46 (16, 198) | 0.8 |
| La | 2.44 (2.02, 3.19) | 3.27 (2.20, 4.13) | 2.00 (1.70, 2.39) | 2.81 (2.22, 3.50) | 2.54 (2.09, 3.12) | 2.50 (2.17, 3.42) | 2.38 (1.73, 3.39) | 2.55 (2.05, 3.68) | 3.17 (2.40, 4.18) | 3.71 (3.11, 4.62) | 2.19 (1.71, 2.84) | 0.014 |
| Ce | 6 (3, 9) | 9 (5, 14) | 7 (3, 217) | 8 (3, 557) | 7 (3, 127) | 9 (5, 44) | 6 (3, 9) | 13 (5, 485) | 17 (10, 487) | 14 (7, 185) | 10 (5, 370) | 0.2 |
| Pr | 0.24 (0.00, 0.82) | 0.31 (0.00, 0.90) | 0.47 (0.00, 0.62) | 0.10 (0.00, 0.69) | 0.35 (0.00, 0.66) | 0.47 (0.00, 0.67) | 0.19 (0.00, 0.90) | 0.51 (0.00, 0.81) | 0.04 (0.00, 1.25) | 0.71 (0.00, 1.07) | 0.19 (0.00, 0.65) | >0.9 |
| Nd | 1.89 (1.26, 3.34) | 3.45 (1.88, 3.94) | 1.76 (1.11, 2.48) | 2.40 (1.75, 3.53) | 2.41 (1.87, 2.87) | 2.62 (1.81, 3.03) | 2.11 (1.05, 3.80) | 2.71 (1.53, 3.29) | 2.51 (1.68, 4.75) | 3.48 (2.62, 4.39) | 1.78 (1.28, 2.74) | 0.13 |
| Sm | 0.10 (0.00, 0.48) | 0.07 (0.00, 0.55) | 0.21 (0.00, 0.35) | 0.00 (0.00, 0.49) | 0.11 (0.00, 0.38) | 0.11 (0.00, 0.31) | 0.09 (0.00, 0.64) | 0.28 (0.00, 0.48) | 0.00 (0.00, 0.82) | 0.41 (0.00, 0.62) | 0.05 (0.00, 0.36) | >0.9 |
| Eu | 0.03 (0.00, 0.17) | 0.06 (0.00, 0.15) | 0.10 (0.00, 0.13) | 0.00 (0.00, 0.16) | 0.04 (0.00, 0.14) | 0.05 (0.00, 0.14) | 0.04 (0.00, 0.19) | 0.12 (0.00, 0.18) | 0.00 (0.00, 0.27) | 0.07 (0.00, 0.22) | 0.03 (0.00, 0.13) | >0.9 |
| Gd | 0.50 (0.00, 1.67) | 0.46 (0.00, 4.24) | 0.44 (0.34, 3.97) | 0.78 (0.24, 5.32) | 0.51 (0.18, 3.83) | 0.45 (0.22, 2.09) | 0.43 (0.00, 0.60) | 0.86 (0.49, 4.37) | 2.68 (0.73, 5.76) | 0.71 (0.48, 4.89) | 0.80 (0.25, 3.57) | 0.5 |
| Tb | 0.014 (0.000, 0.058) | 0.017 (0.000, 0.048) | 0.025 (0.000, 0.038) | 0.000 (0.000, 0.054) | 0.016 (0.000, 0.045) | 0.014 (0.000, 0.041) | 0.012 (0.000, 0.070) | 0.029 (0.000, 0.057) | 0.000 (0.000, 0.086) | 0.021 (0.000, 0.058) | 0.012 (0.000, 0.038) | >0.9 |
| Dy | 0.06 (0.00, 0.28) | 0.09 (0.00, 0.37) | 0.15 (0.00, 0.25) | 0.00 (0.00, 0.30) | 0.11 (0.00, 0.26) | 0.10 (0.00, 0.25) | 0.09 (0.00, 0.37) | 0.19 (0.00, 0.35) | 0.00 (0.00, 0.45) | 0.11 (0.00, 0.40) | 0.08 (0.00, 0.22) | >0.9 |
| Ho | 0.00 (0.00, 0.04) | 0.01 (0.00, 0.07) | 0.01 (0.00, 0.05) | 0.00 (0.00, 0.05) | 0.00 (0.00, 0.05) | 0.00 (0.00, 0.04) | 0.00 (0.00, 0.07) | 0.03 (0.00, 0.07) | 0.00 (0.00, 0.03) | 0.01 (0.00, 0.08) | 0.01 (0.00, 0.04) | >0.9 |
| Er | 0.06 (0.00, 0.31) | 0.01 (0.00, 0.25) | 0.17 (0.00, 0.21) | 0.00 (0.00, 0.41) | 0.13 (0.00, 0.32) | 0.11 (0.00, 0.35) | 0.11 (0.00, 0.34) | 0.22 (0.00, 0.36) | 0.10 (0.00, 0.37) | 0.05 (0.00, 0.30) | 0.06 (0.00, 0.30) | >0.9 |
| Tm | 0.007 (0.000, 0.023) | 0.013 (0.000, 0.039) | 0.008 (0.000, 0.023) | 0.000 (0.000, 0.028) | 0.003 (0.000, 0.023) | 0.008 (0.000, 0.028) | 0.006 (0.000, 0.037) | 0.008 (0.000, 0.028) | 0.000 (0.000, 0.029) | 0.008 (0.000, 0.033) | 0.005 (0.000, 0.025) | >0.9 |
| Yb | 0.07 (0.00, 0.23) | 0.08 (0.00, 0.30) | 0.13 (0.00, 0.21) | 0.00 (0.00, 0.27) | 0.10 (0.00, 0.24) | 0.06 (0.00, 0.26) | 0.08 (0.00, 0.25) | 0.18 (0.00, 0.27) | 0.00 (0.00, 0.32) | 0.10 (0.00, 0.29) | 0.07 (0.00, 0.24) | >0.9 |
| Hf | 1.54 (0.64, 2.77) | 1.75 (0.65, 4.01) | 1.94 (1.23, 3.27) | 2.17 (1.29, 4.77) | 1.48 (0.95, 3.41) | 1.54 (1.07, 2.86) | 1.38 (0.37, 1.76) | 4.23 (1.60, 4.71) | 2.08 (0.86, 4.56) | 2.25 (1.58, 3.75) | 2.83 (0.83, 4.29) | 0.6 |
| Pt | 1.79 (1.35, 2.46) | 2.13 (1.49, 2.74) | 1.59 (0.89, 2.58) | 2.11 (1.15, 3.85) | 1.69 (1.30, 2.23) | 1.78 (1.60, 2.07) | 1.94 (1.69, 2.32) | 2.37 (1.68, 3.43) | 2.22 (1.57, 3.02) | 2.37 (1.73, 3.25) | 2.06 (1.41, 2.43) | 0.6 |
| Hg | 8 (2, 21) | 9 (3, 16) | 16 (5, 19) | 10 (0, 18) | 5 (2, 11) | 8 (0, 17) | 7 (1, 12) | 10 (1, 16) | 6 (2, 12) | 7 (3, 23) | 8 (1, 10) | >0.9 |
| Hg2 | 7 (2, 21) | 9 (3, 16) | 16 (5, 19) | 10 (0, 18) | 5 (2, 11) | 8 (0, 17) | 6 (1, 12) | 10 (1, 16) | 6 (2, 12) | 6 (3, 23) | 8 (2, 11) | >0.9 |
| Tl | 0.00 (0.00, 0.19) | 0.00 (0.00, 0.11) | 0.00 (0.00, 0.12) | 0.00 (0.00, 0.09) | 0.00 (0.00, 0.10) | 0.00 (0.00, 0.26) | 0.00 (0.00, 0.11) | 0.00 (0.00, 0.06) | 0.00 (0.00, 0.03) | 0.00 (0.00, 0.12) | 0.00 (0.00, 0.11) | >0.9 |
| Pb | 18 (9, 24) | 19 (11, 27) | 17 (11, 31) | 25 (17, 32) | 14 (10, 22) | 10 (6, 30) | 10 (6, 24) | 22 (5, 29) | 21 (15, 28) | 22 (13, 35) | 18 (8, 28) | 0.8 |
| Bi | 3 (2, 7) | 3 (1, 5) | 2 (1, 3) | 3 (2, 6) | 2 (1, 3) | 3 (2, 8) | 2 (1, 4) | 2 (1, 3) | 2 (2, 4) | 5 (2, 7) | 2 (2, 5) | 0.6 |
| Th | 0.02 (0.00, 0.41) | 0.08 (0.00, 0.29) | 0.00 (0.00, 0.22) | 0.00 (0.00, 0.42) | 0.08 (0.00, 0.29) | 0.07 (0.00, 0.23) | 0.07 (0.00, 0.32) | 0.00 (0.00, 0.72) | 0.03 (0.00, 0.74) | 0.15 (0.00, 0.44) | 0.00 (0.00, 0.20) | >0.9 |
| U | 0.19 (0.02, 0.50) | 0.26 (0.00, 0.50) | 0.27 (0.00, 0.40) | 0.21 (0.04, 0.59) | 0.29 (0.05, 0.46) | 0.33 (0.03, 0.44) | 0.27 (0.00, 0.55) | 0.32 (0.02, 0.56) | 0.13 (0.00, 0.72) | 0.42 (0.09, 0.59) | 0.13 (0.00, 0.37) | >0.9 |
| *^1^* Median (IQR) | | | | | | | | | | | | |
| *^2^* Kruskal-Wallis rank sum test | | | | | | | | | | | | |
