# Supplementary material for: Assessment of the Concentration of 51 Elements in the Liver and in Various Parts of the Human Brain—Profiling of the Mineral Status
Source: Nutrients. 2023 Jun 19;15(12):2799. doi: 10.3390/nu15122799 (PMC10302479; doi:10.3390/nu15122799)
Supplement: Supplementary file 1 [file nutrients-15-02799-s001.zip › Table S3.doc]

**Table S3.** Statistical examination whether the levels of individual elements differ statistically significantly between women and men for the brain regions from [A] to [K] and the liver samples [L].

[A]

| **Characteristic** | **F**, N = 4*^1^* | **M**, N = 12*^1^* | **p-value***^2^* |
| --- | --- | --- | --- |
| Be | 0.000 (0.000, 0.043) | 0.000 (0.000, 0.026) | 0.9 |
| Na | 1,403 (1,292, 1,517) | 1,873 (1,785, 2,257) | 0.013 |
| Mg | 77 (74, 81) | 95 (75, 114) | 0.3 |
| Al | 6,201 (5,820, 8,663) | 11,235 (8,318, 12,674) | 0.3 |
| P | 1,838 (1,699, 2,114) | 1,903 (1,727, 2,251) | 0.9 |
| K | 2,142 (2,052, 2,172) | 2,043 (1,922, 2,343) | 0.9 |
| Ca | 105 (93, 132) | 145 (125, 165) | 0.2 |
| Ti | 37 (32, 40) | 70 (52, 75) | 0.004 |
| V | 6.03 (5.18, 6.45) | 6.02 (5.37, 6.83) | 0.9 |
| Cr | 0 (0, 0) | 24 (3, 36) | 0.027 |
| Mn | 191 (188, 214) | 216 (178, 235) | 0.9 |
| Fe | 30,557 (29,983, 32,162) | 39,035 (36,692, 44,225) | 0.058 |
| Co | 2.36 (2.32, 2.55) | 2.46 (2.10, 3.01) | 0.9 |
| Ni | 19 (4, 35) | 0 (0, 12) | 0.2 |
| Cu | 3,838 (3,170, 4,413) | 3,619 (3,161, 4,626) | 0.9 |
| Zn | 12,781 (10,804, 15,335) | 14,358 (11,833, 17,824) | 0.4 |
| Ga | 2 (1, 6) | 4 (3, 5) | 0.13 |
| Rb | 1,290 (1,261, 1,430) | 1,389 (1,009, 1,679) | 0.7 |
| Sr | 365 (281, 436) | 328 (282, 413) | 0.9 |
| Zr | 74 (18, 134) | 35 (16, 45) | 0.9 |
| As | 0.12 (0.02, 1.74) | 2.97 (0.87, 6.53) | 0.11 |
| Se | 82 (81, 88) | 97 (78, 128) | 0.5 |
| Mo | 120 (115, 152) | 201 (165, 246) | 0.078 |
| Pd | 0.00 (0.00, 1.92) | 0.00 (0.00, 0.02) | 0.9 |
| Ag | 15 (5, 25) | 5 (3, 10) | 0.4 |
| Cd | 14 (9, 36) | 14 (11, 20) | 0.9 |
| Sn | 4 (3, 90) | 13 (6, 37) | 0.3 |
| Sb | 0 (0, 0) | 2 (0, 3) | 0.12 |
| Cs | 5.00 (3.50, 6.33) | 3.95 (3.50, 4.56) | 0.5 |
| Ba | 52 (43, 100) | 43 (32, 229) | 0.7 |
| La | 1.79 (1.51, 2.32) | 2.69 (2.27, 3.20) | 0.13 |
| Ce | 6 (4, 124) | 6 (3, 9) | 0.9 |
| Pr | 0.00 (0.00, 0.02) | 0.61 (0.00, 0.86) | 0.062 |
| Nd | 0.64 (0.59, 1.05) | 2.28 (1.75, 3.73) | 0.030 |
| Sm | 0.00 (0.00, 0.00) | 0.28 (0.00, 0.58) | 0.045 |
| Eu | 0.00 (0.00, 0.00) | 0.15 (0.00, 0.17) | 0.045 |
| Gd | 0.00 (0.00, 1.12) | 0.62 (0.26, 1.67) | 0.2 |
| Tb | 0.000 (0.000, 0.000) | 0.037 (0.000, 0.061) | 0.045 |
| Dy | 0.00 (0.00, 0.00) | 0.19 (0.00, 0.33) | 0.045 |
| Ho | 0.00 (0.00, 0.00) | 0.02 (0.00, 0.10) | 0.071 |
| Er | 0.00 (0.00, 0.00) | 0.24 (0.00, 0.39) | 0.045 |
| Tm | 0.000 (0.000, 0.000) | 0.015 (0.000, 0.027) | 0.045 |
| Yb | 0.00 (0.00, 0.00) | 0.21 (0.00, 0.24) | 0.045 |
| Hf | 1.64 (0.00, 3.89) | 1.54 (0.73, 2.45) | 0.9 |
| Pt | 2.39 (1.69, 2.96) | 1.65 (1.32, 2.04) | 0.3 |
| Hg | 2 (0, 5) | 9 (6, 26) | 0.10 |
| Hg2 | 2 (0, 6) | 10 (6, 26) | 0.10 |
| Tl | 0.00 (0.00, 0.00) | 0.00 (0.00, 0.72) | 0.2 |
| Pb | 20 (18, 49) | 14 (6, 24) | 0.4 |
| Bi | 11.1 (9.1, 16.6) | 2.7 (1.6, 3.6) | 0.002 |
| Th | 0.02 (0.00, 0.27) | 0.12 (0.00, 0.41) | 0.9 |
| U | 0.07 (0.00, 0.15) | 0.38 (0.08, 0.54) | 0.078 |
| *^1^* Median (IQR) | | | |
| *^2^* Wilcoxon rank sum test; Wilcoxon rank sum exact test | | | |

[B]

| **Characteristic** | **F**, N = 4*^1^* | **M**, N = 12*^1^* | **p-value***^2^* |
| --- | --- | --- | --- |
| Be | 0.05 (0.03, 0.09) | 0.00 (0.00, 0.01) | 0.019 |
| Na | 1,557 (1,534, 1,630) | 1,840 (1,723, 1,950) | 0.13 |
| Mg | 85 (82, 89) | 92 (88, 96) | 0.10 |
| Al | 14,239 (11,371, 16,441) | 10,416 (8,390, 13,487) | 0.6 |
| P | 2,185 (2,092, 2,280) | 2,188 (2,074, 2,231) | 0.9 |
| K | 2,174 (1,976, 2,336) | 2,071 (1,879, 2,223) | 0.7 |
| Ca | 120 (113, 129) | 125 (98, 137) | 0.9 |
| Ti | 40 (32, 43) | 87 (74, 110) | 0.002 |
| V | 9.17 (8.45, 9.65) | 7.07 (5.74, 8.08) | 0.078 |
| Cr | 2 (0, 27) | 17 (10, 45) | 0.4 |
| Mn | 238 (231, 258) | 258 (213, 273) | 0.9 |
| Fe | 58,885 (56,231, 61,119) | 55,794 (50,354, 61,893) | 0.4 |
| Co | 3 (2, 3) | 3 (2, 5) | 0.7 |
| Ni | 3 (2, 6) | 0 (0, 17) | 0.9 |
| Cu | 4,506 (3,632, 5,345) | 4,484 (3,842, 5,117) | 0.9 |
| Zn | 10,775 (9,967, 11,743) | 10,828 (9,397, 14,822) | 0.9 |
| Ga | 4 (2, 11) | 4 (3, 5) | 0.9 |
| Rb | 1,417 (1,402, 1,541) | 1,587 (1,364, 1,637) | 0.9 |
| Sr | 492 (462, 518) | 371 (260, 480) | 0.078 |
| Zr | 8 (0, 58) | 36 (19, 65) | 0.2 |
| As | 0.5 (0.4, 2.3) | 2.7 (0.8, 8.5) | 0.2 |
| Se | 96 (80, 99) | 103 (91, 117) | 0.3 |
| Mo | 196 (148, 242) | 230 (203, 261) | 0.6 |
| Pd | 0.00 (0.00, 0.78) | 0.00 (0.00, 0.06) | 0.9 |
| Ag | 14 (13, 17) | 10 (5, 26) | 0.6 |
| Cd | 11 (4, 33) | 14 (10, 19) | 0.6 |
| Sn | 4 (3, 101) | 23 (16, 54) | 0.2 |
| Sb | 0 (0, 2) | 3 (0, 40) | 0.4 |
| Cs | 6.21 (5.15, 7.01) | 4.96 (3.93, 6.84) | 0.5 |
| Ba | 69 (53, 127) | 57 (26, 257) | 0.9 |
| La | 4.23 (3.36, 4.42) | 3.16 (2.20, 3.51) | 0.4 |
| Ce | 11 (9, 143) | 8 (4, 14) | 0.4 |
| Pr | 0.07 (0.00, 0.15) | 0.58 (0.03, 0.92) | 0.12 |
| Nd | 3.53 (2.72, 3.75) | 2.95 (1.88, 3.94) | 0.9 |
| Sm | 0.00 (0.00, 0.00) | 0.32 (0.00, 0.58) | 0.045 |
| Eu | 0.00 (0.00, 0.00) | 0.12 (0.00, 0.20) | 0.045 |
| Gd | 0 (0, 1) | 1 (0, 4) | 0.2 |
| Tb | 0.000 (0.000, 0.000) | 0.039 (0.000, 0.063) | 0.045 |
| Dy | 0.00 (0.00, 0.00) | 0.31 (0.00, 0.38) | 0.045 |
| Ho | 0.00 (0.00, 0.00) | 0.06 (0.00, 0.08) | 0.045 |
| Er | 0.00 (0.00, 0.00) | 0.22 (0.00, 0.33) | 0.045 |
| Tm | 0.000 (0.000, 0.000) | 0.035 (0.000, 0.041) | 0.045 |
| Yb | 0.00 (0.00, 0.00) | 0.25 (0.00, 0.31) | 0.045 |
| Hf | 0.1 (0.0, 1.1) | 2.0 (1.3, 4.2) | 0.10 |
| Pt | 2.49 (1.90, 3.02) | 1.99 (1.38, 2.46) | 0.4 |
| Hg | 6 (4, 7) | 11 (3, 18) | 0.2 |
| Hg2 | 6 (4, 7) | 11 (3, 19) | 0.2 |
| Tl | 0.00 (0.00, 0.00) | 0.02 (0.00, 0.31) | 0.11 |
| Pb | 23 (19, 31) | 14 (10, 27) | 0.4 |
| Bi | 8.7 (6.6, 11.6) | 2.0 (1.3, 2.9) | 0.008 |
| Th | 0.04 (0.00, 0.10) | 0.12 (0.00, 0.36) | 0.4 |
| U | 0.02 (0.00, 0.10) | 0.36 (0.15, 0.54) | 0.10 |
| *^1^* Median (IQR) | | | |
| *^2^* Wilcoxon rank sum test; Wilcoxon rank sum exact test | | | |

[C]

| **Characteristic** | **F**, N = 4*^1^* | **M**, N = 11*^1^* | **p-value***^2^* |
| --- | --- | --- | --- |
| Be | 0.000 (0.000, 0.057) | 0.000 (0.000, 0.000) | 0.4 |
| Na | 1,592 (1,486, 1,742) | 1,870 (1,652, 2,017) | 0.3 |
| Mg | 79 (75, 88) | 87 (77, 97) | 0.5 |
| Al | 8,580 (5,916, 12,524) | 8,555 (7,617, 10,350) | 0.9 |
| P | 1,984 (1,859, 2,253) | 1,774 (1,705, 2,054) | 0.5 |
| K | 2,121 (1,899, 2,378) | 1,802 (1,713, 2,002) | 0.3 |
| Ca | 95 (88, 101) | 111 (94, 119) | 0.3 |
| Ti | 33 (26, 47) | 68 (56, 138) | 0.078 |
| V | 4.39 (3.39, 6.48) | 4.96 (4.50, 5.28) | 0.9 |
| Cr | 0 (0, 3) | 32 (20, 159) | 0.018 |
| Mn | 216 (202, 240) | 213 (185, 245) | 0.7 |
| Fe | 45,033 (42,697, 51,654) | 42,405 (36,631, 46,855) | 0.5 |
| Co | 3 (3, 5) | 3 (2, 5) | 0.7 |
| Ni | 12 (9, 15) | 0 (0, 5) | 0.085 |
| Cu | 4,515 (3,967, 4,663) | 3,655 (3,067, 4,432) | 0.6 |
| Zn | 12,947 (10,389, 15,272) | 11,298 (10,158, 13,096) | 0.7 |
| Ga | 11 (2, 23) | 4 (2, 12) | 0.9 |
| Rb | 1,450 (1,374, 1,604) | 1,350 (1,126, 1,565) | 0.4 |
| Sr | 265 (239, 301) | 291 (255, 350) | 0.7 |
| Zr | 96 (34, 182) | 31 (12, 50) | 0.5 |
| As | 2.9 (0.0, 6.8) | 1.7 (0.7, 6.9) | 0.6 |
| Se | 96 (86, 114) | 93 (77, 108) | 0.4 |
| Mo | 266 (248, 333) | 223 (188, 242) | 0.2 |
| Pd | 0.00 (0.00, 0.39) | 0.00 (0.00, 0.00) | 0.4 |
| Ag | 7 (7, 11) | 4 (2, 7) | 0.10 |
| Cd | 11 (5, 33) | 16 (9, 21) | 0.5 |
| Sn | 163 (5, 376) | 11 (6, 19) | 0.9 |
| Sb | 0 (0, 48) | 0 (0, 3) | 0.5 |
| Cs | 5.54 (4.99, 6.11) | 4.26 (3.37, 4.71) | 0.10 |
| Ba | 110 (41, 218) | 28 (17, 217) | 0.5 |
| La | 1.63 (1.27, 2.02) | 2.08 (1.84, 2.55) | 0.2 |
| Ce | 214 (6, 503) | 6 (3, 11) | 0.4 |
| Pr | 0.00 (0.00, 0.00) | 0.57 (0.29, 0.65) | 0.018 |
| Nd | 0.83 (0.45, 1.20) | 2.19 (1.59, 2.73) | 0.018 |
| Sm | 0.00 (0.00, 0.00) | 0.31 (0.10, 0.38) | 0.033 |
| Eu | 0.00 (0.00, 0.00) | 0.12 (0.05, 0.14) | 0.033 |
| Gd | 1.99 (0.00, 4.62) | 0.44 (0.39, 2.45) | 0.8 |
| Tb | 0.000 (0.000, 0.000) | 0.032 (0.012, 0.042) | 0.033 |
| Dy | 0.00 (0.00, 0.00) | 0.21 (0.07, 0.27) | 0.033 |
| Ho | 0.000 (0.000, 0.000) | 0.035 (0.004, 0.063) | 0.033 |
| Er | 0.00 (0.00, 0.00) | 0.17 (0.08, 0.27) | 0.033 |
| Tm | 0.000 (0.000, 0.000) | 0.020 (0.004, 0.033) | 0.033 |
| Yb | 0.00 (0.00, 0.00) | 0.18 (0.07, 0.24) | 0.033 |
| Hf | 4.7 (2.4, 10.6) | 1.6 (0.9, 2.7) | 0.14 |
| Pt | 2.85 (2.40, 3.41) | 1.19 (0.83, 1.94) | 0.056 |
| Hg | 7 (1, 15) | 18 (11, 21) | 0.3 |
| Hg2 | 7 (1, 15) | 18 (10, 21) | 0.3 |
| Tl | 0 (0, 0) | 0 (0, 0) | 0.056 |
| Pb | 31 (21, 39) | 16 (7, 22) | 0.2 |
| Bi | 1 (1, 2) | 2 (1, 5) | 0.3 |
| Th | 0.00 (0.00, 0.00) | 0.03 (0.00, 0.28) | 0.090 |
| U | 0.00 (0.00, 0.01) | 0.38 (0.14, 0.42) | 0.039 |
| *^1^* Median (IQR) | | | |
| *^2^* Wilcoxon rank sum test; Wilcoxon rank sum exact test | | | |

[D]

| **Characteristic** | **F**, N = 4*^1^* | **M**, N = 11*^1^* | **p-value***^2^* |
| --- | --- | --- | --- |
| Be | 0.00 (0.00, 0.03) | 0.00 (0.00, 0.00) | 0.7 |
| Na | 1,431 (1,268, 1,619) | 1,619 (1,484, 1,797) | 0.4 |
| Mg | 81 (79, 83) | 97 (77, 104) | 0.4 |
| Al | 17,741 (15,618, 19,316) | 10,117 (7,815, 16,280) | 0.10 |
| P | 1,901 (1,853, 1,970) | 1,899 (1,759, 2,075) | 0.9 |
| K | 2,434 (2,261, 2,545) | 2,230 (2,189, 2,386) | 0.3 |
| Ca | 108 (101, 116) | 117 (103, 137) | 0.4 |
| Ti | 56 (49, 70) | 72 (59, 111) | 0.3 |
| V | 6.50 (5.79, 7.20) | 6.42 (5.50, 7.78) | 0.9 |
| Cr | 23 (0, 49) | 6 (2, 22) | 0.9 |
| Mn | 209 (200, 227) | 203 (191, 223) | 0.7 |
| Fe | 28,121 (25,996, 31,391) | 29,452 (26,527, 31,885) | 0.9 |
| Co | 2.89 (2.66, 4.37) | 2.26 (1.83, 3.85) | 0.3 |
| Ni | 10 (6, 499) | 1 (0, 5) | 0.14 |
| Cu | 4,607 (3,909, 5,157) | 3,622 (3,224, 4,030) | 0.2 |
| Zn | 12,062 (11,501, 37,582) | 12,953 (11,569, 14,851) | 0.8 |
| Ga | 31 (23, 33) | 4 (2, 7) | 0.2 |
| Rb | 1,613 (1,563, 1,758) | 1,532 (1,369, 2,056) | 0.8 |
| Sr | 262 (172, 367) | 371 (313, 455) | 0.3 |
| Zr | 245 (164, 289) | 58 (21, 143) | 0.2 |
| As | 8.7 (5.8, 9.8) | 1.6 (0.4, 5.5) | 0.4 |
| Se | 89 (85, 92) | 88 (79, 107) | 0.9 |
| Mo | 211 (191, 227) | 198 (169, 222) | 0.5 |
| Pd | 0.000 (0.000, 0.000) | 0.000 (0.000, 0.000) | 0.7 |
| Ag | 9 (8, 31) | 8 (6, 13) | 0.8 |
| Cd | 11 (5, 21) | 10 (8, 16) | 0.9 |
| Sn | 494 (311, 577) | 17 (7, 19) | 0.2 |
| Sb | 0.0 (0.0, 0.2) | 0.3 (0.0, 1.3) | 0.4 |
| Cs | 7.46 (6.64, 8.05) | 5.30 (4.53, 6.41) | 0.040 |
| Ba | 311 (240, 324) | 44 (28, 155) | 0.2 |
| La | 2.68 (2.39, 2.96) | 2.81 (2.22, 3.80) | 0.7 |
| Ce | 688 (436, 798) | 6 (3, 10) | 0.056 |
| Pr | 0.00 (0.00, 0.00) | 0.53 (0.07, 0.82) | 0.018 |
| Nd | 1.75 (1.39, 2.06) | 3.03 (2.17, 4.30) | 0.10 |
| Sm | 0.00 (0.00, 0.00) | 0.22 (0.00, 0.55) | 0.056 |
| Eu | 0.00 (0.00, 0.00) | 0.10 (0.00, 0.19) | 0.056 |
| Gd | 6.18 (4.02, 7.13) | 0.48 (0.24, 1.69) | 0.3 |
| Tb | 0.000 (0.000, 0.000) | 0.035 (0.000, 0.071) | 0.056 |
| Dy | 0.00 (0.00, 0.00) | 0.24 (0.00, 0.36) | 0.056 |
| Ho | 0.00 (0.00, 0.00) | 0.01 (0.00, 0.08) | 0.090 |
| Er | 0.00 (0.00, 0.00) | 0.28 (0.00, 0.46) | 0.056 |
| Tm | 0.000 (0.000, 0.000) | 0.018 (0.000, 0.030) | 0.056 |
| Yb | 0.00 (0.00, 0.00) | 0.18 (0.00, 0.30) | 0.056 |
| Hf | 4.77 (3.32, 5.34) | 1.85 (1.29, 3.99) | 0.4 |
| Pt | 4.01 (3.44, 4.21) | 1.78 (0.95, 2.15) | 0.040 |
| Hg | 0 (0, 3) | 16 (2, 23) | 0.077 |
| Hg2 | 0 (0, 3) | 17 (2, 23) | 0.10 |
| Tl | 0.00 (0.00, 0.09) | 0.00 (0.00, 0.09) | 0.9 |
| Pb | 30 (23, 32) | 21 (17, 32) | 0.7 |
| Bi | 2 (1, 170) | 3 (2, 6) | 0.6 |
| Th | 0.00 (0.00, 0.00) | 0.24 (0.00, 0.51) | 0.033 |
| U | 0.06 (0.00, 0.11) | 0.51 (0.14, 0.65) | 0.075 |
| *^1^* Median (IQR) | | | |
| *^2^* Wilcoxon rank sum test; Wilcoxon rank sum exact test | | | |

[E]

| **Characteristic** | **F**, N = 4*^1^* | **M**, N = 12*^1^* | **p-value***^2^* |
| --- | --- | --- | --- |
| Be | 0.05 (0.00, 0.20) | 0.00 (0.00, 0.01) | 0.2 |
| Na | 1,334 (1,192, 1,448) | 1,557 (1,472, 1,649) | 0.030 |
| Mg | 82 (78, 87) | 97 (82, 103) | 0.4 |
| Al | 9,685 (7,879, 11,642) | 9,922 (8,282, 12,907) | 0.8 |
| P | 1,925 (1,753, 2,129) | 1,796 (1,740, 1,925) | 0.7 |
| K | 2,193 (2,080, 2,380) | 2,109 (1,979, 2,295) | 0.7 |
| Ca | 98 (95, 102) | 103 (93, 133) | 0.6 |
| Ti | 45 (36, 53) | 84 (52, 161) | 0.058 |
| V | 5.7 (4.8, 7.3) | 6.0 (4.7, 7.0) | 0.9 |
| Cr | 34 (19, 78) | 14 (3, 41) | 0.6 |
| Mn | 262 (251, 276) | 245 (230, 295) | 0.7 |
| Fe | 34,136 (33,281, 36,250) | 30,026 (28,063, 35,451) | 0.2 |
| Co | 2.42 (2.34, 3.69) | 2.26 (1.88, 3.40) | 0.4 |
| Ni | 8 (4, 35) | 5 (0, 12) | 0.4 |
| Cu | 3,034 (2,350, 3,846) | 2,854 (2,163, 3,485) | 0.7 |
| Zn | 15,112 (14,362, 16,031) | 14,385 (13,364, 17,261) | 0.8 |
| Ga | 13 (2, 28) | 4 (3, 10) | 0.9 |
| Rb | 1,704 (1,613, 1,877) | 1,556 (1,518, 1,721) | 0.3 |
| Sr | 287 (252, 325) | 284 (236, 392) | 0.9 |
| Zr | 45 (18, 87) | 27 (23, 141) | 0.7 |
| As | 2.2 (0.1, 4.7) | 1.6 (0.7, 9.5) | 0.3 |
| Se | 74 (64, 80) | 85 (78, 102) | 0.2 |
| Mo | 144 (85, 201) | 176 (156, 186) | 0.7 |
| Pd | 0.00 (0.00, 0.14) | 0.00 (0.00, 0.01) | 0.9 |
| Ag | 7 (4, 163) | 3 (1, 23) | 0.3 |
| Cd | 13 (7, 32) | 11 (8, 15) | 0.9 |
| Sn | 6 (5, 85) | 17 (7, 107) | 0.3 |
| Sb | 0 (0, 0) | 2 (0, 5) | 0.045 |
| Cs | 6.88 (5.48, 7.92) | 5.33 (4.49, 6.48) | 0.4 |
| Ba | 112 (52, 188) | 40 (29, 334) | 0.6 |
| La | 2.54 (2.09, 2.82) | 2.54 (2.09, 3.38) | 0.7 |
| Ce | 8 (6, 124) | 5 (2, 136) | 0.8 |
| Pr | 0.00 (0.00, 0.00) | 0.60 (0.21, 0.72) | 0.046 |
| Nd | 1.93 (1.47, 2.11) | 2.69 (2.11, 3.32) | 0.13 |
| Sm | 0.00 (0.00, 0.00) | 0.29 (0.00, 0.50) | 0.045 |
| Eu | 0.00 (0.00, 0.00) | 0.11 (0.00, 0.16) | 0.045 |
| Gd | 0.00 (0.00, 1.05) | 0.52 (0.47, 3.88) | 0.11 |
| Tb | 0.000 (0.000, 0.000) | 0.034 (0.000, 0.054) | 0.045 |
| Dy | 0.00 (0.00, 0.00) | 0.25 (0.00, 0.30) | 0.045 |
| Ho | 0.000 (0.000, 0.000) | 0.041 (0.000, 0.050) | 0.045 |
| Er | 0.00 (0.00, 0.00) | 0.27 (0.00, 0.33) | 0.045 |
| Tm | 0.000 (0.000, 0.000) | 0.018 (0.000, 0.027) | 0.045 |
| Yb | 0.00 (0.00, 0.00) | 0.21 (0.00, 0.25) | 0.045 |
| Hf | 0.94 (0.23, 2.01) | 1.75 (1.08, 3.99) | 0.3 |
| Pt | 1.80 (1.51, 2.23) | 1.65 (1.27, 2.17) | 0.8 |
| Hg | 2.6 (0.5, 4.9) | 8.4 (4.0, 12.2) | 0.089 |
| Hg2 | 2.9 (0.7, 5.0) | 8.4 (3.4, 12.3) | 0.2 |
| Tl | 0.00 (0.00, 0.41) | 0.00 (0.00, 0.10) | 0.6 |
| Pb | 35 (15, 70) | 12 (8, 18) | 0.10 |
| Bi | 3 (2, 156) | 1 (1, 3) | 0.13 |
| Th | 0.00 (0.00, 0.00) | 0.20 (0.00, 0.42) | 0.045 |
| U | 0.03 (0.00, 0.18) | 0.35 (0.18, 0.47) | 0.2 |
| *^1^* Median (IQR) | | | |
| *^2^* Wilcoxon rank sum test; Wilcoxon rank sum exact test | | | |

[F]

| **Characteristic** | **F**, N = 4*^1^* | **M**, N = 12*^1^* | **p-value***^2^* |
| --- | --- | --- | --- |
| Be | 0.03 (0.00, 0.10) | 0.00 (0.00, 0.02) | 0.5 |
| Na | 1,212 (1,196, 1,283) | 1,641 (1,594, 1,687) | 0.004 |
| Mg | 90 (86, 92) | 107 (90, 123) | 0.13 |
| Al | 9,780 (8,632, 13,749) | 9,749 (8,756, 16,886) | 0.9 |
| P | 2,151 (1,999, 2,267) | 2,153 (1,885, 2,522) | 0.7 |
| K | 2,397 (2,308, 2,447) | 2,413 (2,017, 2,682) | 0.9 |
| Ca | 84 (83, 93) | 111 (100, 117) | 0.078 |
| Ti | 55 (49, 61) | 65 (51, 92) | 0.3 |
| V | 7.53 (6.61, 7.94) | 5.41 (5.27, 6.50) | 0.6 |
| Cr | 5 (0, 249) | 13 (5, 32) | 0.5 |
| Mn | 504 (452, 509) | 460 (407, 472) | 0.5 |
| Fe | 91,512 (80,573, 111,211) | 105,783 (100,096, 117,884) | 0.3 |
| Co | 3.8 (3.5, 8.0) | 3.2 (3.0, 6.2) | 0.6 |
| Ni | 5 (0, 16) | 0 (0, 3) | 0.3 |
| Cu | 4,898 (4,090, 5,906) | 5,314 (4,140, 5,962) | 0.9 |
| Zn | 11,854 (10,893, 12,943) | 13,173 (12,196, 15,068) | 0.2 |
| Ga | 15 (2, 44) | 4 (2, 7) | 0.9 |
| Rb | 2,171 (1,891, 2,409) | 1,962 (1,674, 2,308) | 0.6 |
| Sr | 237 (218, 280) | 407 (284, 518) | 0.030 |
| Zr | 42 (30, 84) | 42 (25, 124) | 0.8 |
| As | 1.77 (0.86, 3.89) | 1.73 (0.69, 4.98) | 0.8 |
| Se | 104 (101, 109) | 108 (103, 147) | 0.4 |
| Mo | 418 (371, 492) | 599 (524, 685) | 0.078 |
| Pd | 0.00 (0.00, 0.09) | 0.00 (0.00, 0.00) | 0.9 |
| Ag | 95 (5, 217) | 8 (4, 35) | 0.3 |
| Cd | 19 (5, 57) | 18 (15, 25) | 0.9 |
| Sn | 7 (3, 113) | 20 (9, 100) | 0.2 |
| Sb | 1.5 (0.4, 14.5) | 0.1 (0.0, 1.7) | 0.2 |
| Cs | 8.00 (6.28, 9.95) | 6.24 (5.21, 7.43) | 0.4 |
| Ba | 74 (47, 154) | 38 (19, 150) | 0.4 |
| La | 2.90 (2.42, 3.51) | 2.45 (2.17, 3.38) | 0.8 |
| Ce | 8 (7, 155) | 11 (5, 44) | 0.9 |
| Pr | 0.00 (0.00, 0.10) | 0.62 (0.15, 0.69) | 0.062 |
| Nd | 1.70 (1.32, 2.60) | 2.79 (2.21, 3.03) | 0.3 |
| Sm | 0.00 (0.00, 0.00) | 0.28 (0.00, 0.35) | 0.045 |
| Eu | 0.00 (0.00, 0.00) | 0.12 (0.00, 0.15) | 0.045 |
| Gd | 0.00 (0.00, 1.48) | 0.53 (0.36, 2.40) | 0.11 |
| Tb | 0.000 (0.000, 0.000) | 0.036 (0.000, 0.051) | 0.045 |
| Dy | 0.00 (0.00, 0.00) | 0.21 (0.00, 0.28) | 0.045 |
| Ho | 0.000 (0.000, 0.000) | 0.026 (0.000, 0.053) | 0.071 |
| Er | 0.00 (0.00, 0.00) | 0.31 (0.00, 0.42) | 0.045 |
| Tm | 0.000 (0.000, 0.000) | 0.024 (0.000, 0.031) | 0.045 |
| Yb | 0.00 (0.00, 0.00) | 0.19 (0.00, 0.28) | 0.045 |
| Hf | 1.28 (0.84, 2.10) | 1.70 (1.13, 3.34) | 0.4 |
| Pt | 1.88 (1.81, 2.20) | 1.70 (1.37, 2.07) | 0.4 |
| Hg | 1 (0, 2) | 13 (4, 19) | 0.058 |
| Hg2 | 1 (0, 2) | 12 (4, 19) | 0.058 |
| Tl | 0 (0, 11) | 0 (0, 0) | 0.5 |
| Pb | 28 (22, 43) | 9 (5, 29) | 0.2 |
| Bi | 29 (6, 64) | 3 (2, 5) | 0.2 |
| Th | 0.00 (0.00, 0.05) | 0.09 (0.01, 0.35) | 0.2 |
| U | 0.00 (0.00, 0.07) | 0.38 (0.26, 0.48) | 0.013 |
| *^1^* Median (IQR) | | | |
| *^2^* Wilcoxon rank sum test; Wilcoxon rank sum exact test | | | |

[G]

| **Characteristic** | **F**, N = 4*^1^* | **M**, N = 12*^1^* | **p-value***^2^* |
| --- | --- | --- | --- |
| Be | 0.00 (0.00, 0.05) | 0.00 (0.00, 0.00) | 0.8 |
| Na | 1,058 (1,045, 1,093) | 1,216 (1,139, 1,276) | 0.030 |
| Mg | 99 (99, 101) | 121 (105, 130) | 0.058 |
| Al | 7,053 (6,277, 7,758) | 11,620 (8,320, 15,124) | 0.078 |
| P | 3,652 (3,495, 3,750) | 3,606 (3,310, 3,800) | 0.9 |
| K | 2,394 (2,368, 2,424) | 2,464 (2,260, 2,594) | 0.5 |
| Ca | 97 (85, 109) | 102 (87, 111) | 0.9 |
| Ti | 46 (38, 62) | 91 (71, 117) | 0.10 |
| V | 5.17 (4.42, 5.81) | 5.79 (4.15, 7.37) | 0.6 |
| Cr | 0 (0, 0) | 5 (0, 12) | 0.071 |
| Mn | 363 (318, 430) | 374 (343, 397) | 0.9 |
| Fe | 39,010 (29,674, 48,286) | 35,603 (30,096, 45,430) | 0.5 |
| Co | 2.21 (2.15, 2.37) | 2.25 (2.07, 2.81) | 0.9 |
| Ni | 4.8 (4.0, 17.7) | 0.0 (0.0, 1.1) | 0.023 |
| Cu | 3,986 (3,766, 4,406) | 4,046 (3,451, 4,739) | 0.9 |
| Zn | 8,375 (7,899, 9,208) | 8,549 (7,279, 8,874) | 0.6 |
| Ga | 2 (1, 2) | 4 (3, 5) | 0.013 |
| Rb | 2,012 (1,775, 2,252) | 2,059 (1,801, 2,181) | 0.9 |
| Sr | 285 (263, 302) | 344 (234, 391) | 0.4 |
| Zr | 0 (0, 6) | 30 (23, 80) | 0.013 |
| As | 1 (0, 3) | 3 (2, 12) | 0.2 |
| Se | 72 (60, 84) | 84 (75, 98) | 0.3 |
| Mo | 44 (37, 58) | 80 (73, 108) | 0.3 |
| Pd | 0.00 (0.00, 0.00) | 0.00 (0.00, 0.00) | 0.5 |
| Ag | 8 (6, 10) | 8 (3, 14) | 0.9 |
| Cd | 9 (3, 36) | 14 (9, 17) | 0.5 |
| Sn | 2 (0, 3) | 9 (6, 22) | 0.004 |
| Sb | 0.2 (0.0, 7.3) | 0.0 (0.0, 1.1) | 0.9 |
| Cs | 9.26 (6.94, 11.02) | 7.12 (5.51, 7.87) | 0.3 |
| Ba | 52 (44, 151) | 34 (25, 340) | 0.5 |
| La | 1.83 (1.58, 2.13) | 2.89 (1.96, 3.58) | 0.2 |
| Ce | 6 (5, 6) | 5 (3, 10) | 0.9 |
| Pr | 0.00 (0.00, 0.00) | 0.60 (0.00, 1.00) | 0.045 |
| Nd | 1.00 (0.75, 1.32) | 2.47 (1.58, 4.18) | 0.058 |
| Sm | 0.00 (0.00, 0.00) | 0.24 (0.00, 0.67) | 0.045 |
| Eu | 0.00 (0.00, 0.00) | 0.13 (0.00, 0.22) | 0.045 |
| Gd | 0.00 (0.00, 0.00) | 0.54 (0.37, 1.45) | 0.015 |
| Tb | 0.000 (0.000, 0.000) | 0.032 (0.000, 0.072) | 0.045 |
| Dy | 0.00 (0.00, 0.00) | 0.25 (0.00, 0.40) | 0.045 |
| Ho | 0.00 (0.00, 0.00) | 0.03 (0.00, 0.07) | 0.071 |
| Er | 0.00 (0.00, 0.00) | 0.29 (0.00, 0.41) | 0.045 |
| Tm | 0.000 (0.000, 0.000) | 0.030 (0.000, 0.043) | 0.045 |
| Yb | 0.00 (0.00, 0.00) | 0.23 (0.00, 0.30) | 0.045 |
| Hf | 0.00 (0.00, 0.12) | 1.63 (1.33, 2.30) | 0.010 |
| Pt | 1.90 (1.83, 2.06) | 1.99 (1.59, 2.36) | 0.9 |
| Hg | 2 (0, 3) | 10 (4, 13) | 0.045 |
| Hg2 | 2 (0, 3) | 10 (4, 13) | 0.045 |
| Tl | 0.00 (0.00, 6.75) | 0.00 (0.00, 0.11) | 0.9 |
| Pb | 10 (5, 20) | 10 (6, 24) | 0.8 |
| Bi | 2.5 (0.9, 4.9) | 2.3 (1.0, 3.7) | 0.9 |
| Th | 0.00 (0.00, 0.02) | 0.14 (0.00, 0.51) | 0.11 |
| U | 0.00 (0.00, 0.01) | 0.47 (0.16, 0.56) | 0.020 |
| *^1^* Median (IQR) | | | |
| *^2^* Wilcoxon rank sum test; Wilcoxon rank sum exact test | | | |

[H]

| **Characteristic** | **F**, N = 3*^1^* | **M**, N = 12*^1^* | **p-value***^2^* |
| --- | --- | --- | --- |
| Be | 0.000 (0.000, 0.000) | 0.000 (0.000, 0.016) | 0.4 |
| Na | 1,099 (1,081, 1,111) | 1,253 (1,196, 1,357) | 0.048 |
| Mg | 94 (92, 96) | 129 (112, 131) | 0.070 |
| Al | 11,808 (10,822, 14,060) | 11,858 (9,350, 15,329) | 0.9 |
| P | 3,587 (3,229, 3,626) | 3,584 (3,364, 3,660) | 0.8 |
| K | 2,059 (2,057, 2,172) | 2,295 (2,212, 2,648) | 0.10 |
| Ca | 82 (78, 96) | 121 (100, 134) | 0.2 |
| Ti | 87 (62, 92) | 79 (67, 131) | 0.7 |
| V | 4.60 (4.03, 5.38) | 6.14 (4.92, 7.55) | 0.2 |
| Cr | 7 (4, 14) | 22 (5, 60) | 0.3 |
| Mn | 314 (301, 370) | 345 (323, 367) | 0.4 |
| Fe | 34,012 (30,942, 38,458) | 39,982 (37,275, 41,767) | 0.5 |
| Co | 1.78 (1.75, 2.27) | 2.72 (2.16, 3.22) | 0.3 |
| Ni | 13 (7, 14) | 1 (0, 26) | 0.4 |
| Cu | 3,291 (3,042, 3,456) | 3,707 (3,089, 4,824) | 0.4 |
| Zn | 7,748 (7,633, 7,871) | 9,077 (8,080, 10,121) | 0.10 |
| Ga | 20 (13, 24) | 5 (4, 18) | 0.3 |
| Rb | 1,792 (1,726, 1,930) | 1,981 (1,673, 2,146) | 0.4 |
| Sr | 253 (225, 300) | 422 (263, 692) | 0.2 |
| Zr | 213 (107, 222) | 95 (24, 166) | 0.6 |
| As | 7.87 (3.96, 8.04) | 3.83 (1.90, 8.25) | 0.9 |
| Se | 70 (64, 70) | 94 (82, 98) | 0.048 |
| Mo | 89 (49, 116) | 94 (84, 121) | 0.8 |
| Pd | 0.000 (0.000, 0.000) | 0.000 (0.000, 0.000) | 0.7 |
| Ag | 5 (5, 6) | 4 (2, 13) | 0.7 |
| Cd | 4 (3, 44) | 14 (10, 19) | 0.4 |
| Sn | 351 (176, 420) | 23 (13, 122) | 0.7 |
| Sb | 0 (0, 0) | 3 (0, 11) | 0.3 |
| Cs | 7.80 (6.35, 9.75) | 6.65 (5.55, 8.58) | 0.8 |
| Ba | 188 (121, 241) | 49 (31, 270) | 0.4 |
| La | 2.55 (2.15, 2.77) | 2.83 (2.09, 3.81) | 0.5 |
| Ce | 478 (243, 571) | 12 (5, 133) | 0.3 |
| Pr | 0.00 (0.00, 0.00) | 0.59 (0.16, 0.95) | 0.053 |
| Nd | 1.23 (1.10, 1.53) | 2.86 (2.23, 3.80) | 0.10 |
| Sm | 0.00 (0.00, 0.00) | 0.33 (0.00, 0.63) | 0.080 |
| Eu | 0.00 (0.00, 0.00) | 0.14 (0.00, 0.19) | 0.080 |
| Gd | 4.36 (2.18, 5.29) | 0.73 (0.50, 3.25) | 0.7 |
| Tb | 0.000 (0.000, 0.000) | 0.034 (0.000, 0.075) | 0.080 |
| Dy | 0.00 (0.00, 0.00) | 0.24 (0.00, 0.40) | 0.080 |
| Ho | 0.00 (0.00, 0.00) | 0.06 (0.00, 0.08) | 0.080 |
| Er | 0.00 (0.00, 0.00) | 0.27 (0.10, 0.37) | 0.053 |
| Tm | 0.000 (0.000, 0.000) | 0.023 (0.000, 0.035) | 0.080 |
| Yb | 0.00 (0.00, 0.00) | 0.22 (0.00, 0.27) | 0.080 |
| Hf | 4.68 (2.34, 5.05) | 3.91 (1.71, 4.70) | 0.9 |
| Pt | 3.31 (2.89, 3.70) | 1.86 (1.66, 3.40) | 0.2 |
| Hg | 0 (0, 2) | 11 (4, 20) | 0.069 |
| Hg2 | 0 (0, 2) | 11 (4, 20) | 0.069 |
| Tl | 0.00 (0.00, 0.00) | 0.00 (0.00, 0.08) | 0.2 |
| Pb | 22 (13, 25) | 20 (4, 32) | 0.9 |
| Bi | 3 (2, 6) | 2 (1, 3) | 0.7 |
| Th | 0.00 (0.00, 0.00) | 0.16 (0.00, 0.75) | 0.12 |
| U | 0.01 (0.01, 0.03) | 0.41 (0.13, 0.62) | 0.10 |
| *^1^* Median (IQR) | | | |
| *^2^* Wilcoxon rank sum test; Wilcoxon rank sum exact test | | | |

[I]

| **Characteristic** | **F**, N = 3*^1^* | **M**, N = 9*^1^* | **p-value***^2^* |
| --- | --- | --- | --- |
| Be | 0.00 (0.00, 0.01) | 0.00 (0.00, 0.02) | 0.8 |
| Na | 1,532 (1,508, 1,645) | 1,632 (1,478, 1,770) | 0.9 |
| Mg | 105 (96, 107) | 105 (100, 114) | 0.6 |
| Al | 21,145 (19,142, 21,365) | 13,147 (11,051, 16,511) | 0.10 |
| P | 2,651 (2,462, 2,871) | 2,494 (2,387, 2,899) | 0.9 |
| K | 2,633 (2,502, 2,704) | 2,232 (1,966, 2,416) | 0.15 |
| Ca | 109 (100, 112) | 113 (99, 127) | 0.9 |
| Ti | 63 (54, 90) | 85 (71, 100) | 0.7 |
| V | 6.60 (5.81, 6.79) | 7.97 (4.92, 8.65) | 0.7 |
| Cr | 9 (4, 10) | 0 (0, 7) | 0.7 |
| Mn | 400 (391, 403) | 347 (327, 367) | 0.036 |
| Fe | 73,181 (71,187, 73,649) | 51,381 (44,654, 73,166) | 0.3 |
| Co | 4.07 (3.69, 4.54) | 3.48 (2.61, 4.35) | 0.6 |
| Ni | 5 (3, 5) | 3 (0, 5) | 0.5 |
| Cu | 4,427 (3,238, 4,545) | 2,805 (2,563, 3,730) | 0.9 |
| Zn | 12,114 (11,897, 12,119) | 10,581 (10,015, 13,228) | 0.9 |
| Ga | 35 (30, 37) | 6 (5, 10) | 0.009 |
| Rb | 2,378 (2,270, 2,417) | 1,765 (1,723, 1,957) | 0.2 |
| Sr | 277 (211, 291) | 402 (278, 535) | 0.2 |
| Zr | 262 (235, 278) | 17 (12, 135) | 0.009 |
| As | 10.0 (9.1, 10.7) | 2.5 (1.5, 5.9) | 0.064 |
| Se | 132 (126, 143) | 129 (110, 145) | 0.7 |
| Mo | 257 (257, 295) | 228 (224, 235) | 0.036 |
| Pd | 0.00 (0.00, 0.00) | 0.00 (0.00, 0.01) | 0.3 |
| Ag | 8 (5, 9) | 6 (3, 29) | 0.9 |
| Cd | 8 (7, 83) | 18 (14, 27) | 0.5 |
| Sn | 553 (496, 589) | 21 (14, 46) | 0.009 |
| Sb | 0 (0, 0) | 0 (0, 2) | 0.093 |
| Cs | 9.75 (8.98, 11.19) | 6.34 (5.75, 7.20) | 0.064 |
| Ba | 296 (277, 326) | 73 (48, 199) | 0.15 |
| La | 3.01 (2.59, 3.59) | 3.32 (2.48, 4.20) | 0.9 |
| Ce | 750 (665, 800) | 11 (9, 19) | 0.009 |
| Pr | 0.00 (0.00, 0.04) | 0.80 (0.00, 1.25) | 0.3 |
| Nd | 1.70 (1.21, 2.61) | 2.74 (1.91, 4.86) | 0.3 |
| Sm | 0.00 (0.00, 0.00) | 0.45 (0.00, 0.88) | 0.15 |
| Eu | 0.00 (0.00, 0.00) | 0.24 (0.00, 0.27) | 0.15 |
| Gd | 6.89 (6.14, 7.27) | 0.85 (0.58, 3.93) | 0.042 |
| Tb | 0.00 (0.00, 0.00) | 0.04 (0.00, 0.10) | 0.15 |
| Dy | 0.00 (0.00, 0.00) | 0.24 (0.00, 0.50) | 0.15 |
| Ho | 0.00 (0.00, 0.00) | 0.01 (0.00, 0.04) | 0.15 |
| Er | 0.00 (0.00, 0.00) | 0.22 (0.00, 0.40) | 0.093 |
| Tm | 0.000 (0.000, 0.000) | 0.026 (0.000, 0.030) | 0.15 |
| Yb | 0.00 (0.00, 0.00) | 0.30 (0.00, 0.33) | 0.15 |
| Hf | 5.53 (4.88, 5.65) | 0.92 (0.79, 3.01) | 0.064 |
| Pt | 4.61 (3.92, 4.74) | 1.71 (1.43, 2.63) | 0.009 |
| Hg | 2 (1, 4) | 10 (3, 13) | 0.2 |
| Hg2 | 2 (1, 4) | 10 (3, 13) | 0.2 |
| Tl | 0.00 (0.00, 0.00) | 0.00 (0.00, 0.11) | 0.2 |
| Pb | 27 (26, 29) | 17 (12, 23) | 0.2 |
| Bi | 1.8 (1.7, 2.6) | 2.9 (1.7, 4.6) | 0.7 |
| Th | 0.00 (0.00, 0.03) | 0.54 (0.00, 0.81) | 0.3 |
| U | 0.00 (0.00, 0.08) | 0.66 (0.03, 0.74) | 0.2 |
| *^1^* Median (IQR) | | | |
| *^2^* Wilcoxon rank sum test; Wilcoxon rank sum exact test | | | |

[J]

| **Characteristic** | **F**, N = 4*^1^* | **M**, N = 12*^1^* | **p-value***^2^* |
| --- | --- | --- | --- |
| Be | 0.07 (0.00, 0.27) | 0.00 (0.00, 0.08) | 0.7 |
| Na | 1,239 (1,149, 1,397) | 1,387 (1,288, 1,494) | 0.4 |
| Mg | 117 (107, 124) | 123 (106, 131) | 0.4 |
| Al | 13,867 (11,504, 20,255) | 13,950 (9,731, 19,488) | 0.6 |
| P | 2,970 (2,778, 3,260) | 2,748 (2,512, 3,004) | 0.4 |
| K | 3,060 (2,905, 3,203) | 2,779 (2,532, 2,996) | 0.4 |
| Ca | 126 (92, 167) | 117 (106, 133) | 0.9 |
| Ti | 67 (59, 85) | 98 (77, 128) | 0.4 |
| V | 7.31 (6.18, 10.04) | 7.17 (5.72, 8.76) | 0.9 |
| Cr | 9 (0, 20) | 19 (7, 45) | 0.3 |
| Mn | 496 (436, 563) | 489 (461, 544) | 0.9 |
| Fe | 98,437 (83,515, 121,961) | 104,887 (81,930, 131,327) | 0.9 |
| Co | 4.23 (2.95, 5.50) | 3.86 (3.02, 5.32) | 0.9 |
| Ni | 7 (6, 162) | 0 (0, 8) | 0.11 |
| Cu | 5,952 (5,124, 6,896) | 5,609 (4,930, 6,224) | 0.5 |
| Zn | 19,796 (12,698, 26,354) | 13,586 (11,872, 15,280) | 0.7 |
| Ga | 8 (5, 14) | 5 (4, 11) | 0.9 |
| Rb | 2,559 (2,353, 2,788) | 2,423 (1,934, 2,702) | 0.5 |
| Sr | 557 (209, 942) | 365 (290, 401) | 0.9 |
| Zr | 72 (58, 97) | 66 (29, 184) | 0.9 |
| As | 2 (0, 4) | 4 (1, 9) | 0.4 |
| Se | 123 (112, 142) | 129 (117, 145) | 0.7 |
| Mo | 412 (329, 546) | 604 (323, 825) | 0.5 |
| Pd | 0.00 (0.00, 0.00) | 0.00 (0.00, 0.03) | 0.2 |
| Ag | 8 (7, 9) | 16 (4, 35) | 0.4 |
| Cd | 15 (7, 53) | 20 (14, 28) | 0.6 |
| Sn | 315 (210, 350) | 27 (11, 132) | 0.7 |
| Sb | 0.8 (0.0, 2.2) | 0.7 (0.0, 2.4) | 0.8 |
| Cs | 10.01 (9.58, 10.96) | 7.62 (6.49, 8.87) | 0.058 |
| Ba | 145 (95, 186) | 62 (30, 314) | 0.8 |
| La | 3.98 (2.92, 6.01) | 3.71 (3.11, 4.62) | 0.9 |
| Ce | 77 (60, 185) | 9 (6, 143) | 0.3 |
| Pr | 0.00 (0.00, 0.37) | 0.81 (0.40, 1.07) | 0.2 |
| Nd | 3.21 (2.08, 5.41) | 3.53 (2.73, 4.39) | 0.8 |
| Sm | 0.00 (0.00, 0.24) | 0.43 (0.12, 0.62) | 0.3 |
| Eu | 0.00 (0.00, 0.00) | 0.16 (0.00, 0.23) | 0.045 |
| Gd | 0.53 (0.08, 1.92) | 0.71 (0.52, 5.19) | 0.4 |
| Tb | 0.00 (0.00, 0.00) | 0.05 (0.00, 0.07) | 0.045 |
| Dy | 0.00 (0.00, 0.00) | 0.30 (0.00, 0.45) | 0.045 |
| Ho | 0.00 (0.00, 0.00) | 0.03 (0.00, 0.09) | 0.045 |
| Er | 0.00 (0.00, 0.00) | 0.29 (0.00, 0.33) | 0.045 |
| Tm | 0.000 (0.000, 0.000) | 0.026 (0.000, 0.042) | 0.045 |
| Yb | 0.00 (0.00, 0.00) | 0.25 (0.00, 0.32) | 0.045 |
| Hf | 1.44 (1.02, 2.10) | 2.60 (1.75, 4.68) | 0.2 |
| Pt | 2.69 (1.95, 3.52) | 2.37 (1.65, 2.82) | 0.4 |
| Hg | 2 (1, 3) | 15 (6, 23) | 0.029 |
| Hg2 | 2 (1, 4) | 15 (6, 23) | 0.029 |
| Tl | 0 (0, 0) | 0 (0, 0) | 0.6 |
| Pb | 27 (20, 32) | 21 (5, 40) | 0.9 |
| Bi | 3.6 (2.6, 5.3) | 5.2 (2.2, 8.8) | 0.6 |
| Th | 0.00 (0.00, 0.30) | 0.27 (0.01, 0.44) | 0.3 |
| U | 0.05 (0.00, 0.21) | 0.46 (0.28, 0.68) | 0.13 |
| *^1^* Median (IQR) | | | |
| *^2^* Wilcoxon rank sum test; Wilcoxon rank sum exact test | | | |

[K]

| **Characteristic** | **F**, N = 4*^1^* | **M**, N = 12*^1^* | **p-value***^2^* |
| --- | --- | --- | --- |
| Be | 0.00 (0.00, 0.02) | 0.00 (0.00, 0.00) | 0.9 |
| Na | 1,228 (1,205, 1,272) | 1,619 (1,531, 1,673) | 0.013 |
| Mg | 81 (76, 87) | 96 (91, 104) | 0.020 |
| Al | 11,209 (9,754, 12,579) | 10,169 (7,126, 15,857) | 0.9 |
| P | 1,867 (1,800, 1,994) | 2,017 (1,696, 2,337) | 0.9 |
| K | 2,574 (2,469, 2,641) | 2,420 (2,333, 2,845) | 0.8 |
| Ca | 81 (73, 93) | 93 (83, 108) | 0.3 |
| Ti | 59 (46, 90) | 72 (46, 137) | 0.9 |
| V | 4.81 (3.78, 5.85) | 4.79 (4.35, 6.53) | 0.8 |
| Cr | 7 (2, 12) | 7 (1, 12) | 0.9 |
| Mn | 214 (206, 221) | 218 (198, 240) | 0.9 |
| Fe | 34,481 (29,929, 38,070) | 33,895 (30,147, 41,999) | 0.8 |
| Co | 2.44 (2.41, 3.45) | 2.56 (2.23, 3.55) | 0.9 |
| Ni | 16 (13, 25) | 1 (0, 6) | 0.021 |
| Cu | 3,839 (3,145, 4,520) | 3,776 (3,327, 4,690) | 0.9 |
| Zn | 13,484 (13,242, 13,968) | 13,602 (12,856, 15,272) | 0.9 |
| Ga | 12 (3, 21) | 4 (3, 14) | 0.9 |
| Rb | 1,931 (1,791, 2,104) | 1,936 (1,723, 2,117) | 0.9 |
| Sr | 273 (199, 329) | 334 (246, 411) | 0.3 |
| Zr | 103 (39, 172) | 29 (11, 122) | 0.6 |
| As | 2.97 (0.09, 6.41) | 1.38 (0.27, 4.95) | 0.9 |
| Se | 92 (90, 96) | 102 (84, 117) | 0.6 |
| Mo | 251 (215, 275) | 234 (193, 258) | 0.6 |
| Pd | 0.00 (0.00, 0.00) | 0.00 (0.00, 0.04) | 0.2 |
| Ag | 15 (7, 32) | 10 (3, 17) | 0.5 |
| Cd | 11 (5, 34) | 13 (9, 17) | 0.8 |
| Sn | 176 (11, 368) | 13 (6, 113) | 0.8 |
| Sb | 1 (0, 24) | 0 (0, 2) | 0.4 |
| Cs | 8.38 (6.95, 9.23) | 5.91 (5.17, 7.67) | 0.3 |
| Ba | 132 (61, 215) | 30 (14, 150) | 0.2 |
| La | 1.92 (1.56, 2.38) | 2.29 (1.71, 2.95) | 0.4 |
| Ce | 240 (10, 510) | 8 (3, 101) | 0.3 |
| Pr | 0.00 (0.00, 0.00) | 0.48 (0.04, 0.73) | 0.027 |
| Nd | 1.02 (0.72, 1.45) | 2.14 (1.35, 3.12) | 0.042 |
| Sm | 0.00 (0.00, 0.00) | 0.20 (0.00, 0.40) | 0.045 |
| Eu | 0.00 (0.00, 0.00) | 0.08 (0.00, 0.14) | 0.045 |
| Gd | 2.17 (0.00, 4.79) | 0.80 (0.31, 3.01) | 0.9 |
| Tb | 0.00 (0.00, 0.00) | 0.03 (0.00, 0.05) | 0.045 |
| Dy | 0.00 (0.00, 0.00) | 0.20 (0.00, 0.26) | 0.045 |
| Ho | 0.00 (0.00, 0.00) | 0.04 (0.00, 0.06) | 0.045 |
| Er | 0.00 (0.00, 0.00) | 0.24 (0.00, 0.33) | 0.045 |
| Tm | 0.000 (0.000, 0.000) | 0.018 (0.000, 0.034) | 0.045 |
| Yb | 0.00 (0.00, 0.00) | 0.18 (0.00, 0.27) | 0.045 |
| Hf | 3.50 (2.35, 4.41) | 2.14 (0.83, 4.29) | 0.6 |
| Pt | 2.57 (2.09, 3.04) | 1.85 (1.06, 2.21) | 0.2 |
| Hg | 5 (1, 9) | 9 (4, 12) | 0.4 |
| Hg2 | 5 (2, 9) | 9 (4, 12) | 0.4 |
| Tl | 0.00 (0.00, 0.00) | 0.05 (0.00, 0.12) | 0.071 |
| Pb | 20 (16, 29) | 15 (4, 28) | 0.6 |
| Bi | 4.11 (3.52, 4.68) | 2.02 (1.63, 5.01) | 0.4 |
| Th | 0.00 (0.00, 0.00) | 0.03 (0.00, 0.46) | 0.045 |
| U | 0.00 (0.00, 0.00) | 0.29 (0.05, 0.40) | 0.010 |
| *^1^* Median (IQR) | | | |
| *^2^* Wilcoxon rank sum test; Wilcoxon rank sum exact test | | | |

[L]

| **Characteristic** | **F**, N = 4*^1^* | **M**, N = 12*^1^* | **p-value***^2^* |
| --- | --- | --- | --- |
| Be | 0.06 (0.00, 0.12) | 0.04 (0.00, 0.16) | 0.8 |
| Na | 968 (788, 1,153) | 912 (730, 1,151) | 0.7 |
| Mg | 95 (87, 102) | 108 (82, 121) | 0.3 |
| Al | 12,325 (9,547, 17,413) | 9,294 (7,698, 14,206) | 0.7 |
| P | 1,813 (1,740, 1,900) | 1,665 (1,566, 2,059) | 0.6 |
| K | 1,770 (1,550, 1,964) | 1,996 (1,686, 2,121) | 0.3 |
| Ca | 112 (98, 129) | 102 (85, 110) | 0.4 |
| Ti | 84 (73, 96) | 102 (64, 111) | 0.7 |
| V | 20.6 (17.2, 21.1) | 7.9 (5.8, 10.2) | 0.030 |
| Cr | 0 (0, 24) | 19 (3, 42) | 0.2 |
| Mn | 1,437 (1,323, 1,514) | 955 (852, 1,197) | 0.042 |
| Fe | 72,622 (49,433, 103,958) | 127,440 (97,039, 204,728) | 0.13 |
| Co | 40 (38, 46) | 31 (19, 39) | 0.13 |
| Ni | 20 (15, 53) | 0 (0, 5) | 0.014 |
| Cu | 2,911 (2,711, 3,077) | 4,138 (2,362, 5,268) | 0.4 |
| Zn | 39,652 (36,144, 41,061) | 48,363 (31,964, 52,665) | 0.4 |
| Ga | 9 (4, 16) | 5 (3, 24) | 0.9 |
| Rb | 2,545 (1,986, 3,205) | 2,945 (2,057, 3,073) | 0.9 |
| Sr | 387 (348, 465) | 450 (347, 555) | 0.8 |
| Zr | 48 (11, 114) | 58 (20, 128) | 0.8 |
| As | 3 (3, 5) | 5 (3, 13) | 0.8 |
| Se | 188 (181, 209) | 188 (163, 211) | 0.9 |
| Mo | 3,251 (2,929, 3,588) | 3,441 (1,545, 6,732) | 0.9 |
| Pd | 0.00 (0.00, 0.00) | 0.00 (0.00, 0.02) | 0.2 |
| Ag | 4 (4, 4) | 11 (4, 27) | 0.2 |
| Cd | 813 (516, 1,450) | 452 (321, 1,095) | 0.3 |
| Sn | 165 (13, 348) | 32 (16, 137) | 0.9 |
| Sb | 0.23 (0.00, 1.06) | 0.90 (0.16, 2.81) | 0.4 |
| Cs | 11.5 (7.9, 14.6) | 8.5 (6.7, 10.3) | 0.6 |
| Ba | 109 (65, 183) | 97 (20, 271) | 0.8 |
| La | 12 (12, 15) | 8 (5, 12) | 0.10 |
| Ce | 94 (34, 265) | 17 (12, 160) | 0.2 |
| Pr | 0.48 (0.24, 0.66) | 1.10 (0.33, 1.55) | 0.2 |
| Nd | 3.86 (3.48, 4.59) | 4.08 (2.29, 5.71) | 0.9 |
| Sm | 0.00 (0.00, 0.00) | 0.20 (0.00, 0.49) | 0.071 |
| Eu | 0.00 (0.00, 0.00) | 0.08 (0.00, 0.15) | 0.071 |
| Gd | 2 (1, 3) | 1 (0, 6) | 0.9 |
| Tb | 0.000 (0.000, 0.000) | 0.026 (0.000, 0.061) | 0.071 |
| Dy | 0.00 (0.00, 0.00) | 0.14 (0.00, 0.36) | 0.071 |
| Ho | 0.000 (0.000, 0.000) | 0.022 (0.000, 0.053) | 0.071 |
| Er | 0.00 (0.00, 0.23) | 0.15 (0.00, 0.36) | 0.6 |
| Tm | 0.000 (0.000, 0.000) | 0.014 (0.000, 0.036) | 0.071 |
| Yb | 0.00 (0.00, 0.00) | 0.16 (0.00, 0.36) | 0.071 |
| Hf | 1.04 (0.03, 2.53) | 2.29 (1.12, 3.83) | 0.5 |
| Pt | 2.63 (2.28, 3.00) | 1.75 (1.47, 3.13) | 0.4 |
| Hg | 22 (9, 36) | 14 (8, 24) | 0.6 |
| Hg2 | 22 (9, 35) | 14 (9, 24) | 0.7 |
| Tl | 0.00 (0.00, 0.03) | 0.10 (0.00, 0.30) | 0.2 |
| Pb | 49 (27, 140) | 58 (46, 98) | 0.9 |
| Bi | 3 (2, 4) | 3 (1, 7) | 0.8 |
| Th | 0.00 (0.00, 0.13) | 0.07 (0.00, 0.39) | 0.5 |
| U | 0.00 (0.00, 0.07) | 0.35 (0.23, 0.70) | 0.038 |
| *^1^* Median (IQR) | | | |
| *^2^* Wilcoxon rank sum test; Wilcoxon rank sum exact test | | | |
