# Supplementary material for: Assessment of the Concentration of 51 Elements in the Liver and in Various Parts of the Human Brain—Profiling of the Mineral Status
Source: Nutrients. 2023 Jun 19;15(12):2799. doi: 10.3390/nu15122799 (PMC10302479; doi:10.3390/nu15122799)
Supplement: Supplementary file 1 [file nutrients-15-02799-s001.zip › Table S4.doc]

**Table S4.** Study of the correlation between the content of individual elements in the liver (L) and various areas of the brain (from A to K).

| **ELEMENT** | **PARAMETER1** | **PARAMETER2** | **R** | **CI** | | **CI_LOW** | | **CI_HIGH** | **T** | **DF_ERROR** | **P** | **METHOD** | **N_OBS** |
| --- | --- | --- | --- | --- | --- | --- | --- | --- | --- | --- | --- | --- | --- |
| Be | A | L | 0.26453209 | 0.95 | | -0.266061935 | | 0.6721044 | 1.02635023 | 14 | 3.221341e-01 | Pearson | 16 |
| Na | A | L | 0.13592557 | 0.95 | | -0.385772752 | | 0.5917588 | 0.51335131 | 14 | 6.157108e-01 | Pearson | 16 |
| Mg | A | L | 0.58376034 | 0.95 | | 0.123911729 | | 0.8372019 | 2.69018495 | 14 | 1.759145e-02 | Pearson | 16 |
| Al | A | L | 0.03896571 | 0.95 | | -0.465735537 | | 0.5245393 | 0.14590716 | 14 | 8.860747e-01 | Pearson | 16 |
| P | A | L | 0.49074298 | 0.95 | | -0.006557565 | | 0.7934344 | 2.10740603 | 14 | 5.359340e-02 | Pearson | 16 |
| K | A | L | 0.33711437 | 0.95 | | -0.190410306 | | 0.7135747 | 1.33979301 | 14 | 2.016554e-01 | Pearson | 16 |
| Ca | A | L | -0.08408164 | 0.95 | | -0.556588517 | | 0.4295262 | -0.31572270 | 14 | 7.568705e-01 | Pearson | 16 |
| Ti | A | L | 0.37862458 | 0.95 | | -0.144132414 | | 0.7361624 | 1.53063904 | 14 | 1.481375e-01 | Pearson | 16 |
| V | A | L | -0.29070024 | 0.95 | | -0.687356377 | | 0.2395204 | -1.13679409 | 14 | 2.747086e-01 | Pearson | 16 |
| Cr | A | L | 0.02204905 | 0.95 | | -0.478890472 | | 0.5121566 | 0.08252005 | 14 | 9.354014e-01 | Pearson | 16 |
| Mn | A | L | 0.07722186 | 0.95 | | -0.435140329 | | 0.5518045 | 0.28980313 | 14 | 7.762147e-01 | Pearson | 16 |
| Fe | A | L | 0.07660135 | 0.95 | | -0.435646202 | | 0.5513702 | 0.28746064 | 14 | 7.779708e-01 | Pearson | 16 |
| Co | A | L | 0.51916208 | 0.95 | | 0.031585261 | | 0.8071470 | 2.27282276 | 14 | 3.932244e-02 | Pearson | 16 |
| Ni | A | L | 0.03978992 | 0.95 | | -0.465088860 | | 0.5251374 | 0.14899823 | 14 | 8.836800e-01 | Pearson | 16 |
| Cu | A | L | 0.65375474 | 0.95 | | 0.233825017 | | 0.8681266 | 3.23259577 | 14 | 6.017480e-03 | Pearson | 16 |
| Zn | A | L | 0.63753442 | 0.95 | | 0.207361333 | | 0.8611053 | 3.09627164 | 14 | 7.890608e-03 | Pearson | 16 |
| Ga | A | L | 0.15739699 | 0.95 | | -0.366937752 | | 0.6058336 | 0.59635897 | 14 | 5.604552e-01 | Pearson | 16 |
| Rb | A | L | 0.60941349 | 0.95 | | 0.162926561 | | 0.8487272 | 2.87596527 | 14 | 1.220808e-02 | Pearson | 16 |
| Sr | A | L | 0.43890898 | 0.95 | | -0.072589642 | | 0.7676065 | 1.82770107 | 14 | 8.898167e-02 | Pearson | 16 |
| Zr | A | L | 0.74729081 | 0.95 | | 0.399618617 | | 0.9070078 | 4.20785286 | 14 | 8.770527e-04 | Pearson | 16 |
| As | A | L | -0.06894054 | 0.95 | | -0.545987195 | | 0.4418652 | -0.25856705 | 14 | 7.997320e-01 | Pearson | 16 |
| Se | A | L | 0.93383290 | 0.95 | | 0.815737136 | | 0.9771908 | 9.76793259 | 14 | 1.248473e-07 | Pearson | 16 |
| Mo | A | L | 0.57175912 | 0.95 | | 0.106135003 | | 0.8317317 | 2.60759454 | 14 | 2.066947e-02 | Pearson | 16 |
| Pd | A | L | -0.06880301 | 0.95 | | -0.545890199 | | 0.4419764 | -0.25804880 | 14 | 8.001239e-01 | Pearson | 16 |
| Ag | A | L | 0.05033503 | 0.95 | | -0.456767253 | | 0.5327476 | 0.18857549 | 14 | 8.531318e-01 | Pearson | 16 |
| Cd | A | L | 0.76253760 | 0.95 | | 0.428986626 | | 0.9130976 | 4.41020386 | 14 | 5.930558e-04 | Pearson | 16 |
| Sn | A | L | 0.79048219 | 0.95 | | 0.484707785 | | 0.9240874 | 4.82903815 | 14 | 2.675714e-04 | Pearson | 16 |
| Sb | A | L | -0.10077581 | 0.95 | | -0.568101566 | | 0.4156956 | -0.37899797 | 14 | 7.103754e-01 | Pearson | 16 |
| Cs | A | L | 0.73665539 | 0.95 | | 0.379546948 | | 0.9027199 | 4.07576704 | 14 | 1.134603e-03 | Pearson | 16 |
| Ba | A | L | -0.02660389 | 0.95 | | -0.515510826 | | 0.4753705 | -0.09957791 | 14 | 9.220913e-01 | Pearson | 16 |
| La | A | L | -0.18916737 | 0.95 | | -0.626157148 | | 0.3382567 | -0.72081389 | 14 | 4.828876e-01 | Pearson | 16 |
| Ce | A | L | 0.83052361 | 0.95 | | 0.569123616 | | 0.9394586 | 5.57922314 | 14 | 6.792539e-05 | Pearson | 16 |
| Pr | A | L | 0.35586578 | 0.95 | | -0.169791560 | | 0.7238761 | 1.42479934 | 14 | 1.761253e-01 | Pearson | 16 |
| Nd | A | L | -0.18376326 | 0.95 | | -0.622741551 | | 0.3432055 | -0.69949112 | 14 | 4.957039e-01 | Pearson | 16 |
| Sm | A | L | 0.43385604 | 0.95 | | -0.078795404 | | 0.7650303 | 1.80174552 | 14 | 9.315359e-02 | Pearson | 16 |
| Eu | A | L | 0.50577311 | 0.95 | | 0.013436501 | | 0.8007251 | 2.19369778 | 14 | 4.563601e-02 | Pearson | 16 |
| Gd | A | L | 0.38253663 | 0.95 | | -0.139649866 | | 0.7382508 | 1.54914876 | 14 | 1.436507e-01 | Pearson | 16 |
| Tb | A | L | 0.59645421 | 0.95 | | 0.143041241 | | 0.8429332 | 2.78045889 | 14 | 1.473608e-02 | Pearson | 16 |
| Dy | A | L | 0.51151514 | 0.95 | | 0.021180307 | | 0.8034875 | 2.22736149 | 14 | 4.284191e-02 | Pearson | 16 |
| Ho | A | L | 0.39902632 | 0.95 | | -0.120517255 | | 0.7469795 | 1.62826429 | 14 | 1.257563e-01 | Pearson | 16 |
| Er | A | L | 0.35903781 | 0.95 | | -0.166257523 | | 0.7256026 | 1.43936930 | 14 | 1.720292e-01 | Pearson | 16 |
| Tm | A | L | 0.67756868 | 0.95 | | 0.273838783 | | 0.8782817 | 3.44713720 | 14 | 3.926935e-03 | Pearson | 16 |
| Yb | A | L | 0.58268306 | 0.95 | | 0.122303873 | | 0.8367130 | 2.68266491 | 14 | 1.785222e-02 | Pearson | 16 |
| Hf | A | L | 0.49668797 | 0.95 | | 0.001303605 | | 0.7963286 | 2.14123027 | 14 | 5.033200e-02 | Pearson | 16 |
| Pt | A | L | 0.77579118 | 0.95 | | 0.455101382 | | 0.9183372 | 4.60030438 | 14 | 4.122250e-04 | Pearson | 16 |
| Hg | A | L | 0.28159406 | 0.95 | | -0.248847282 | | 0.6820883 | 1.09806313 | 14 | 2.907036e-01 | Pearson | 16 |
| Hg | A | L | 0.28159406 | 0.95 | | -0.248847282 | | 0.6820883 | 1.09806313 | 14 | 2.907036e-01 | Pearson | 16 |
| Tl | A | L | 0.25105405 | 0.95 | | -0.279425446 | | 0.6641115 | 0.97043842 | 14 | 3.482963e-01 | Pearson | 16 |
| Pb | A | L | 0.05413439 | 0.95 | | -0.453747113 | | 0.5354705 | 0.20284977 | 14 | 8.421710e-01 | Pearson | 16 |
| Bi | A | L | -0.09326588 | 0.95 | | -0.562944920 | | 0.4219471 | -0.35049671 | 14 | 7.311836e-01 | Pearson | 16 |
| Th | A | L | 0.11129099 | 0.95 | | -0.406859794 | | 0.5752606 | 0.41901573 | 14 | 6.815624e-01 | Pearson | 16 |
| U | A | L | -0.05239370 | 0.95 | | -0.534224246 | | 0.4551322 | -0.19630891 | 14 | 8.471894e-01 | Pearson | 16 |
| ELEMENT | PARAMETER1 | PARAMETER2 | R | | CI | | CI_LOW | CI_HIGH | T | DF_ERROR | P | METHOD | N_OBS |
| Be | B | L | -0.027318486 | | 0.95 | | -0.51603570 | 0.4748168 | -0.10225458 | 14 | 9.200048e-01 | Pearson | 16 |
| Na | B | L | 0.014054880 | | 0.95 | | -0.48502969 | 0.5062332 | 0.05259374 | 14 | 9.587987e-01 | Pearson | 16 |
| Mg | B | L | 0.268129144 | | 0.95 | | -0.26246063 | 0.6742216 | 1.04137961 | 14 | 3.153501e-01 | Pearson | 16 |
| Al | B | L | 0.253329601 | | 0.95 | | -0.27718356 | 0.6654676 | 0.97983476 | 14 | 3.437974e-01 | Pearson | 16 |
| P | B | L | 0.532858445 | | 0.95 | | 0.05048943 | 0.8136465 | 2.35613730 | 14 | 3.356833e-02 | Pearson | 16 |
| K | B | L | 0.209074448 | | 0.95 | | -0.31977182 | 0.6385961 | 0.79996438 | 14 | 4.370956e-01 | Pearson | 16 |
| Ca | B | L | -0.026282597 | | 0.95 | | -0.51527471 | 0.4756193 | -0.09837446 | 14 | 9.230296e-01 | Pearson | 16 |
| Ti | B | L | 0.237474398 | | 0.95 | | -0.29268503 | 0.6559617 | 0.91471434 | 14 | 3.758275e-01 | Pearson | 16 |
| V | B | L | 0.503729090 | | 0.95 | | 0.01069409 | 0.7997387 | 2.18180956 | 14 | 4.666278e-02 | Pearson | 16 |
| Cr | B | L | -0.017752724 | | 0.95 | | -0.50897898 | 0.4821960 | -0.06643508 | 14 | 9.479709e-01 | Pearson | 16 |
| Mn | B | L | -0.154268526 | | 0.95 | | -0.60380021 | 0.3697091 | -0.58421362 | 14 | 5.683716e-01 | Pearson | 16 |
| Fe | B | L | 0.440400446 | | 0.95 | | -0.07075030 | 0.7683649 | 1.83540312 | 14 | 8.777621e-02 | Pearson | 16 |
| Co | B | L | -0.074913809 | | 0.95 | | -0.55018782 | 0.4370203 | -0.28109167 | 14 | 7.827517e-01 | Pearson | 16 |
| Ni | B | L | -0.121430078 | | 0.95 | | -0.58209693 | 0.3982472 | -0.45773701 | 14 | 6.541648e-01 | Pearson | 16 |
| Cu | B | L | 0.373701356 | | 0.95 | | -0.14974328 | 0.7335244 | 1.50748081 | 14 | 1.539179e-01 | Pearson | 16 |
| Zn | B | L | 0.111117310 | | 0.95 | | -0.40700653 | 0.5751429 | 0.41835364 | 14 | 6.820351e-01 | Pearson | 16 |
| Ga | B | L | 0.076410019 | | 0.95 | | -0.43580212 | 0.5512363 | 0.28673840 | 14 | 7.785125e-01 | Pearson | 16 |
| Rb | B | L | -0.159492360 | | 0.95 | | -0.60719233 | 0.3650763 | -0.60450392 | 14 | 5.551797e-01 | Pearson | 16 |
| Sr | B | L | 0.218378542 | | 0.95 | | -0.31099212 | 0.6443338 | 0.83730681 | 14 | 4.164852e-01 | Pearson | 16 |
| Zr | B | L | 0.755950551 | | 0.95 | | 0.41621170 | 0.9104748 | 4.32078195 | 14 | 7.046510e-04 | Pearson | 16 |
| As | B | L | 0.869040795 | | 0.95 | | 0.65588140 | 0.9538422 | 6.57244221 | 14 | 1.244321e-05 | Pearson | 16 |
| Se | B | L | 0.737974957 | | 0.95 | | 0.38201916 | 0.9032537 | 4.09178198 | 14 | 1.099643e-03 | Pearson | 16 |
| Mo | B | L | 0.063806680 | | 0.95 | | -0.44600550 | 0.5423576 | 0.23923022 | 14 | 8.143938e-01 | Pearson | 16 |
| Pd | B | L | -0.034186327 | | 0.95 | | -0.52106157 | 0.4694749 | -0.12798834 | 14 | 8.999785e-01 | Pearson | 16 |
| Ag | B | L | 0.146081079 | | 0.95 | | -0.37691808 | 0.5984507 | 0.55251236 | 14 | 5.893104e-01 | Pearson | 16 |
| Cd | B | L | -0.155740789 | | 0.95 | | -0.60475788 | 0.3684061 | -0.58992699 | 14 | 5.646402e-01 | Pearson | 16 |
| Sn | B | L | 0.755590756 | | 0.95 | | 0.41551775 | 0.9103312 | 4.31598769 | 14 | 7.112110e-04 | Pearson | 16 |
| Sb | B | L | -0.033114535 | | 0.95 | | -0.52027945 | 0.4703110 | -0.12397123 | 14 | 9.031004e-01 | Pearson | 16 |
| Cs | B | L | 0.376203517 | | 0.95 | | -0.14689584 | 0.7348665 | 1.51923192 | 14 | 1.509614e-01 | Pearson | 16 |
| Ba | B | L | 0.873882446 | | 0.95 | | 0.66720048 | 0.9556231 | 6.72601905 | 14 | 9.687856e-06 | Pearson | 16 |
| La | B | L | -0.124048768 | | 0.95 | | -0.58385208 | 0.3960077 | -0.46776092 | 14 | 6.471538e-01 | Pearson | 16 |
| Ce | B | L | 0.799546181 | | 0.95 | | 0.50333052 | 0.9276051 | 4.98102710 | 14 | 2.014856e-04 | Pearson | 16 |
| Pr | B | L | 0.228279681 | | 0.95 | | -0.30154874 | 0.6503875 | 0.87730918 | 14 | 3.951247e-01 | Pearson | 16 |
| Nd | B | L | -0.225698747 | | 0.95 | | -0.64881465 | 0.3040204 | -0.86685471 | 14 | 4.006351e-01 | Pearson | 16 |
| Sm | B | L | 0.446874368 | | 0.95 | | -0.06272587 | 0.7716462 | 1.86905533 | 14 | 8.267896e-02 | Pearson | 16 |
| Eu | B | L | 0.607067413 | | 0.95 | | 0.15929955 | 0.8476826 | 2.85841227 | 14 | 1.263844e-02 | Pearson | 16 |
| Gd | B | L | -0.067473017 | | 0.95 | | -0.54495150 | 0.4430509 | -0.25303756 | 14 | 8.039169e-01 | Pearson | 16 |
| Tb | B | L | 0.715407070 | | 0.95 | | 0.34042815 | 0.8940533 | 3.83108330 | 14 | 1.834731e-03 | Pearson | 16 |
| Dy | B | L | 0.640992495 | | 0.95 | | 0.21295091 | 0.8626094 | 3.12472571 | 14 | 7.456949e-03 | Pearson | 16 |
| Ho | B | L | 0.434292207 | | 0.95 | | -0.07826129 | 0.7652531 | 1.80397766 | 14 | 9.278809e-02 | Pearson | 16 |
| Er | B | L | 0.349928449 | | 0.95 | | -0.17637032 | 0.7206320 | 1.39767860 | 14 | 1.839646e-01 | Pearson | 16 |
| Tm | B | L | 0.742315400 | | 0.95 | | 0.39018710 | 0.9050060 | 4.14519407 | 14 | 9.908033e-04 | Pearson | 16 |
| Yb | B | L | 0.197282250 | | 0.95 | | -0.33077039 | 0.6312547 | 0.75296073 | 14 | 4.639477e-01 | Pearson | 16 |
| Hf | B | L | -0.176654964 | | 0.95 | | -0.61822322 | 0.3496706 | -0.67154383 | 14 | 5.128035e-01 | Pearson | 16 |
| Pt | B | L | 0.728565196 | | 0.95 | | 0.36450058 | 0.8994359 | 3.97975915 | 14 | 1.369357e-03 | Pearson | 16 |
| Hg | B | L | 0.338952022 | | 0.95 | | -0.18841002 | 0.7145915 | 1.34804146 | 14 | 1.990533e-01 | Pearson | 16 |
| Hg | B | L | 0.338952022 | | 0.95 | | -0.18841002 | 0.7145915 | 1.34804146 | 14 | 1.990533e-01 | Pearson | 16 |
| Tl | B | L | -0.044690224 | | 0.95 | | -0.52868353 | 0.4612329 | -0.16738274 | 14 | 8.694626e-01 | Pearson | 16 |
| Pb | B | L | 0.078090155 | | 0.95 | | -0.43443191 | 0.5524118 | 0.29308159 | 14 | 7.737591e-01 | Pearson | 16 |
| Bi | B | L | 0.007675349 | | 0.95 | | -0.48989389 | 0.5014727 | 0.02871937 | 14 | 9.774938e-01 | Pearson | 16 |
| Th | B | L | 0.334578548 | | 0.95 | | -0.19316339 | 0.7121690 | 1.32843907 | 14 | 2.052822e-01 | Pearson | 16 |
| U | B | L | -0.066742707 | | 0.95 | | -0.54443554 | 0.4436403 | -0.25028642 | 14 | 8.060014e-01 | Pearson | 16 |

| ELEMENT | | PARAMETER1 | | PARAMETER2 | | R | | CI | | CI_LOW | CI_HIGH | | T | | DF_ERROR | | P | | METHOD | | N_OBS |
| --- | --- | --- | --- | --- | --- | --- | --- | --- | --- | --- | --- | --- | --- | --- | --- | --- | --- | --- | --- | --- | --- |
| Be | | C | | L | | 0.030340130 | | 0.95 | | -0.48953107 | 0.5342988 | | 0.10944328 | | 13 | | 0.9145222859 | | Pearson | | 15 |
| Na | | C | | L | | 0.491594897 | | 0.95 | | -0.02762451 | 0.8019149 | | 2.03539474 | | 13 | | 0.0627254520 | | Pearson | | 15 |
| Mg | | C | | L | | 0.562185194 | | 0.95 | | 0.07011434 | 0.8342075 | | 2.45097905 | | 13 | | 0.0291592869 | | Pearson | | 15 |
| Al | | C | | L | | 0.009141841 | | 0.95 | | -0.50548822 | 0.5189743 | | 0.03296275 | | 13 | | 0.9742050192 | | Pearson | | 15 |
| P | | C | | L | | 0.520250121 | | 0.95 | | 0.01088934 | 0.8152460 | | 2.19643779 | | 13 | | 0.0468026592 | | Pearson | | 15 |
| K | | C | | L | | 0.017759225 | | 0.95 | | -0.49904361 | 0.5252437 | | 0.06404189 | | 13 | | 0.9499111549 | | Pearson | | 15 |
| Ca | | C | | L | | 0.166958645 | | 0.95 | | -0.37759900 | 0.6257068 | | 0.61054766 | | 13 | | 0.5520241924 | | Pearson | | 15 |
| Ti | | C | | L | | 0.040098126 | | 0.95 | | -0.48206674 | 0.5412434 | | 0.14469222 | | 13 | | 0.8871730419 | | Pearson | | 15 |
| V | | C | | L | | 0.116135882 | | 0.95 | | -0.42118409 | 0.5931132 | | 0.42158661 | | 13 | | 0.6802144322 | | Pearson | | 15 |
| Cr | | C | | L | | -0.004007322 | | 0.95 | | -0.51521254 | 0.5093010 | | -0.01444872 | | 13 | | 0.9886913818 | | Pearson | | 15 |
| Mn | | C | | L | | 0.181579049 | | 0.95 | | -0.36459725 | 0.6347956 | | 0.66575993 | | 13 | | 0.5172040846 | | Pearson | | 15 |
| Fe | | C | | L | | -0.095183775 | | 0.95 | | -0.57920509 | 0.4384579 | | -0.34475527 | | 13 | | 0.7357920455 | | Pearson | | 15 |
| Co | | C | | L | | -0.040691714 | | 0.95 | | -0.54166366 | 0.4816102 | | -0.14683768 | | 13 | | 0.8855128739 | | Pearson | | 15 |
| Ni | | C | | L | | 0.099152444 | | 0.95 | | -0.43521594 | 0.5818613 | | 0.35926961 | | 13 | | 0.7251616267 | | Pearson | | 15 |
| Cu | | C | | L | | 0.692558867 | | 0.95 | | 0.27943002 | 0.8893168 | | 3.46158893 | | 13 | | 0.0042133493 | | Pearson | | 15 |
| Zn | | C | | L | | 0.495285019 | | 0.95 | | -0.02274982 | 0.8036491 | | 2.05561500 | | 13 | | 0.0604809580 | | Pearson | | 15 |
| Ga | | C | | L | | -0.106454822 | | 0.95 | | -0.58672204 | 0.4292143 | | -0.38602187 | | 13 | | 0.7057239631 | | Pearson | | 15 |
| Rb | | C | | L | | 0.348063134 | | 0.95 | | -0.19982922 | 0.7301418 | | 1.33866472 | | 13 | | 0.2036206295 | | Pearson | | 15 |
| Sr | | C | | L | | -0.425142539 | | 0.95 | | -0.76976280 | 0.1113764 | | -1.69354601 | | 13 | | 0.1141610502 | | Pearson | | 15 |
| Zr | | C | | L | | 0.475978833 | | 0.95 | | -0.04798370 | 0.7945175 | | 1.95139342 | | 13 | | 0.0728962190 | | Pearson | | 15 |
| As | | C | | L | | 0.385089190 | | 0.95 | | -0.15842582 | 0.7495004 | | 1.50448572 | | 13 | | 0.1563593013 | | Pearson | | 15 |
| Se | | C | | L | | 0.374539912 | | 0.95 | | -0.17042025 | 0.7440476 | | 1.45643565 | | 13 | | 0.1689975486 | | Pearson | | 15 |
| Mo | | C | | L | | -0.145912558 | | 0.95 | | -0.61240112 | 0.3959455 | | -0.53178666 | | 13 | | 0.6038430483 | | Pearson | | 15 |
| Pd | | C | | L | | -0.082427920 | | 0.95 | | -0.57059742 | 0.4487848 | | -0.29821290 | | 13 | | 0.7702536039 | | Pearson | | 15 |
| Ag | | C | | L | | -0.140906044 | | 0.95 | | -0.60919652 | 0.4002470 | | -0.51316382 | | 13 | | 0.6164457098 | | Pearson | | 15 |
| Cd | | C | | L | | -0.051021675 | | 0.95 | | -0.54893722 | 0.4736199 | | -0.18420118 | | 13 | | 0.8566979316 | | Pearson | | 15 |
| Sn | | C | | L | | 0.582695318 | | 0.95 | | 0.10040169 | 0.8432529 | | 2.58516291 | | 13 | | 0.0226340214 | | Pearson | | 15 |
| Sb | | C | | L | | -0.089877485 | | 0.95 | | -0.57563746 | 0.4427709 | | -0.32537473 | | 13 | | 0.7500748898 | | Pearson | | 15 |
| Cs | | C | | L | | 0.622835962 | | 0.95 | | 0.16238204 | 0.8605389 | | 2.87040321 | | 13 | | 0.0131329591 | | Pearson | | 15 |
| Ba | | C | | L | | 0.270271549 | | 0.95 | | -0.28087901 | 0.6873682 | | 1.01214581 | | 13 | | 0.3299367473 | | Pearson | | 15 |
| La | | C | | L | | -0.180679843 | | 0.95 | | -0.63424022 | 0.3654031 | | -0.66235144 | | 13 | | 0.5193163431 | | Pearson | | 15 |
| Ce | | C | | L | | 0.675661863 | | 0.95 | | 0.24989010 | 0.8824829 | | 3.30452377 | | 13 | | 0.0056979126 | | Pearson | | 15 |
| Pr | | C | | L | | 0.277871634 | | 0.95 | | -0.27329258 | 0.6916786 | | 1.04295381 | | 13 | | 0.3159773986 | | Pearson | | 15 |
| Nd | | C | | L | | -0.157642688 | | 0.95 | | -0.61984987 | 0.3857731 | | -0.57558580 | | 13 | | 0.5747238203 | | Pearson | | 15 |
| Sm | | C | | L | | 0.612690781 | | 0.95 | | 0.14636627 | 0.8562211 | | 2.79517210 | | 13 | | 0.0151692457 | | Pearson | | 15 |
| Eu | | C | | L | | 0.599272139 | | 0.95 | | 0.12555179 | 0.8504574 | | 2.69904303 | | 13 | | 0.0182271950 | | Pearson | | 15 |
| Gd | | C | | L | | 0.305910790 | | 0.95 | | -0.24469785 | 0.7073302 | | 1.15851609 | | 13 | | 0.2674934532 | | Pearson | | 15 |
| Tb | | C | | L | | 0.534766389 | | 0.95 | | 0.03099410 | 0.8218821 | | 2.28180800 | | 13 | | 0.0399848567 | | Pearson | | 15 |
| Dy | | C | | L | | 0.580853576 | | 0.95 | | 0.09764496 | 0.8424467 | | 2.57282139 | | 13 | | 0.0231696376 | | Pearson | | 15 |
| Ho | | C | | L | | 0.454249127 | | 0.95 | | -0.07560711 | 0.7840642 | | 1.83843869 | | 13 | | 0.0889482328 | | Pearson | | 15 |
| Er | | C | | L | | 0.323836929 | | 0.95 | | -0.22590046 | 0.7171346 | | 1.23411343 | | 13 | | 0.2390059343 | | Pearson | | 15 |
| Tm | | C | | L | | 0.418317382 | | 0.95 | | -0.11956737 | 0.7663585 | | 1.66053422 | | 13 | | 0.1207210589 | | Pearson | | 15 |
| Yb | | C | | L | | 0.763726019 | | 0.95 | | 0.41306658 | 0.9171670 | | 4.26565524 | | 13 | | 0.0009200518 | | Pearson | | 15 |
| Hf | | C | | L | | -0.272348436 | | 0.95 | | -0.68854906 | 0.2788127 | | -1.02054415 | | 13 | | 0.3260877361 | | Pearson | | 15 |
| Pt | | C | | L | | 0.555105794 | | 0.95 | | 0.05986663 | 0.8310509 | | 2.40624083 | | 13 | | 0.0317122897 | | Pearson | | 15 |
| Hg | | C | | L | | 0.762849709 | | 0.95 | | 0.41132380 | 0.9168330 | | 4.25393829 | | 13 | | 0.0009402923 | | Pearson | | 15 |
| Hg | | C | | L | | 0.762849709 | | 0.95 | | 0.41132380 | 0.9168330 | | 4.25393829 | | 13 | | 0.0009402923 | | Pearson | | 15 |
| Tl | | C | | L | | 0.003707030 | | 0.95 | | -0.50952339 | 0.5149919 | | 0.01336598 | | 13 | | 0.9895387570 | | Pearson | | 15 |
| Pb | | C | | L | | -0.075526962 | | 0.95 | | -0.56589550 | 0.4543131 | | -0.27309636 | | 13 | | 0.7890694149 | | Pearson | | 15 |
| Bi | | C | | L | | -0.083953186 | | 0.95 | | -0.57163235 | 0.4475574 | | -0.30376992 | | 13 | | 0.7661105070 | | Pearson | | 15 |
| Th | | C | | L | | 0.436881586 | | 0.95 | | -0.09711554 | 0.7755727 | | 1.75115691 | | 13 | | 0.1034620951 | | Pearson | | 15 |
| U | | C | | L | | -0.045450258 | | 0.95 | | -0.54502360 | 0.4779402 | | -0.16404276 | | 13 | | 0.8722205007 | | Pearson | | 15 |
| ELEMENT | PARAMETER1 | | PARAMETER2 | | R | | CI | | CI_LOW | | | CI_HIGH | | T | | DF_ERROR | | P | | METHOD | N_OBS |
| Be | D | | L | | 0.521427723 | | 0.95 | | 0.012505089 | | | 0.815787232 | | 2.203263107 | | 13 | | 4.621965e-02 | | Pearson | 15 |
| Na | D | | L | | 0.371127648 | | 0.95 | | -0.174265704 | | | 0.742273278 | | 1.441035865 | | 13 | | 1.732254e-01 | | Pearson | 15 |
| Mg | D | | L | | 0.287927336 | | 0.95 | | -0.263148451 | | | 0.697336847 | | 1.084043569 | | 13 | | 2.980414e-01 | | Pearson | 15 |
| Al | D | | L | | 0.490028292 | | 0.95 | | -0.029686574 | | | 0.801177042 | | 2.026854305 | | 13 | | 6.369638e-02 | | Pearson | 15 |
| P | D | | L | | 0.334917553 | | 0.95 | | -0.214072879 | | | 0.723118056 | | 1.281576753 | | 13 | | 2.223783e-01 | | Pearson | 15 |
| K | D | | L | | -0.137541970 | | 0.95 | | -0.607034589 | | | 0.403124043 | | -0.500673052 | | 13 | | 6.249704e-01 | | Pearson | 15 |
| Ca | D | | L | | 0.242306427 | | 0.95 | | -0.308213258 | | | 0.671250576 | | 0.900482842 | | 13 | | 3.842417e-01 | | Pearson | 15 |
| Ti | D | | L | | 0.440245336 | | 0.95 | | -0.092988369 | | | 0.777226930 | | 1.767865987 | | 13 | | 1.005301e-01 | | Pearson | 15 |
| V | D | | L | | 0.148715061 | | 0.95 | | -0.393527154 | | | 0.614188303 | | 0.542229320 | | 13 | | 5.968332e-01 | | Pearson | 15 |
| Cr | D | | L | | 0.579661684 | | 0.95 | | 0.095864873 | | | 0.841924260 | | 2.564866339 | | 13 | | 2.352137e-02 | | Pearson | 15 |
| Mn | D | | L | | -0.118927060 | | 0.95 | | -0.594944732 | | | 0.418853117 | | -0.431862545 | | 13 | | 6.729177e-01 | | Pearson | 15 |
| Fe | D | | L | | 0.186121657 | | 0.95 | | -0.360513722 | | | 0.637594221 | | 0.683005533 | | 13 | | 5.065936e-01 | | Pearson | 15 |
| Co | D | | L | | 0.429447084 | | 0.95 | | -0.106172661 | | | 0.771899847 | | 1.714546735 | | 13 | | 1.101529e-01 | | Pearson | 15 |
| Ni | D | | L | | -0.008918945 | | 0.95 | | -0.518811421 | | | 0.505654154 | | -0.032158994 | | 13 | | 9.748338e-01 | | Pearson | 15 |
| Cu | D | | L | | 0.434532180 | | 0.95 | | -0.099987308 | | | 0.774414474 | | 1.739540425 | | 13 | | 1.055450e-01 | | Pearson | 15 |
| Zn | D | | L | | 0.040132978 | | 0.95 | | -0.482039948 | | | 0.541268090 | | 0.144818185 | | 13 | | 8.870756e-01 | | Pearson | 15 |
| Ga | D | | L | | -0.016291729 | | 0.95 | | -0.524179952 | | | 0.500145150 | | -0.058748461 | | 13 | | 9.540459e-01 | | Pearson | 15 |
| Rb | D | | L | | 0.150946105 | | 0.95 | | -0.391596585 | | | 0.615607653 | | 0.550552152 | | 13 | | 5.912762e-01 | | Pearson | 15 |
| Sr | D | | L | | 0.042549855 | | 0.95 | | -0.480179318 | | | 0.542977575 | | 0.153554753 | | 13 | | 8.803188e-01 | | Pearson | 15 |
| Zr | D | | L | | 0.458370203 | | 0.95 | | -0.070430038 | | | 0.786061153 | | 1.859528714 | | 13 | | 8.572780e-02 | | Pearson | 15 |
| As | D | | L | | -0.129380883 | | 0.95 | | -0.601760814 | | | 0.410059481 | | -0.470443500 | | 13 | | 6.458333e-01 | | Pearson | 15 |
| Se | D | | L | | 0.924698814 | | 0.95 | | 0.783635242 | | | 0.975078059 | | 8.757692222 | | 13 | | 8.192210e-07 | | Pearson | 15 |
| Mo | D | | L | | 0.435767150 | | 0.95 | | -0.098478867 | | | 0.775023562 | | 1.745641164 | | 13 | | 1.044465e-01 | | Pearson | 15 |
| Pd | D | | L | | -0.048133449 | | 0.95 | | -0.546911118 | | | 0.475862731 | | -0.173749010 | | 13 | | 8.647391e-01 | | Pearson | 15 |
| Ag | D | | L | | -0.001331626 | | 0.95 | | -0.513244364 | | | 0.511279984 | | -0.004801249 | | 13 | | 9.962421e-01 | | Pearson | 15 |
| Cd | D | | L | | 0.738072561 | | 0.95 | | 0.363089003 | | | 0.907297775 | | 3.944085181 | | 13 | | 1.680195e-03 | | Pearson | 15 |
| Sn | D | | L | | 0.623265050 | | 0.95 | | 0.163064810 | | | 0.860720805 | | 2.873636133 | | 13 | | 1.305177e-02 | | Pearson | 15 |
| Sb | D | | L | | -0.156127248 | | 0.95 | | -0.618892214 | | | 0.387094731 | | -0.569913678 | | 13 | | 5.784527e-01 | | Pearson | 15 |
| Cs | D | | L | | 0.366393905 | | 0.95 | | -0.179572990 | | | 0.739803115 | | 1.419784027 | | 13 | | 1.792046e-01 | | Pearson | 15 |
| Ba | D | | L | | -0.073026213 | | 0.95 | | -0.564183725 | | | 0.456306423 | | -0.264004641 | | 13 | | 7.959152e-01 | | Pearson | 15 |
| La | D | | L | | -0.511033453 | | 0.95 | | -0.810992010 | | | 0.001665353 | | -2.143603181 | | 13 | | 5.155483e-02 | | Pearson | 15 |
| Ce | D | | L | | 0.515955052 | | 0.95 | | 0.005018664 | | | 0.813267542 | | 2.171688530 | | 13 | | 4.897514e-02 | | Pearson | 15 |
| Pr | D | | L | | 0.283589576 | | 0.95 | | -0.267539352 | | | 0.694902294 | | 1.066271744 | | 13 | | 3.057037e-01 | | Pearson | 15 |
| Nd | D | | L | | -0.047677240 | | 0.95 | | -0.546590550 | | | 0.476216369 | | -0.172098444 | | 13 | | 8.660104e-01 | | Pearson | 15 |
| Sm | D | | L | | 0.456324821 | | 0.95 | | -0.073003100 | | | 0.785070871 | | 1.849042483 | | 13 | | 8.731566e-02 | | Pearson | 15 |
| Eu | D | | L | | 0.506964174 | | 0.95 | | -0.007157452 | | | 0.809103676 | | 2.120597493 | | 13 | | 5.376201e-02 | | Pearson | 15 |
| Gd | D | | L | | 0.009075676 | | 0.95 | | -0.505537478 | | | 0.518925965 | | 0.032724162 | | 13 | | 9.743917e-01 | | Pearson | 15 |
| Tb | D | | L | | 0.539811973 | | 0.95 | | 0.038078929 | | | 0.824170548 | | 2.312132327 | | 13 | | 3.779781e-02 | | Pearson | 15 |
| Dy | D | | L | | 0.622494139 | | 0.95 | | 0.161838435 | | | 0.860394009 | | 2.867830804 | | 13 | | 1.319792e-02 | | Pearson | 15 |
| Ho | D | | L | | 0.237445325 | | 0.95 | | -0.312873733 | | | 0.668406887 | | 0.881326433 | | 13 | | 3.941442e-01 | | Pearson | 15 |
| Er | D | | L | | 0.179438006 | | 0.95 | | -0.366514653 | | | 0.633472430 | | 0.657647016 | | 13 | | 5.222399e-01 | | Pearson | 15 |
| Tm | D | | L | | 0.433946052 | | 0.95 | | -0.100702369 | | | 0.774125175 | | 1.736649194 | | 13 | | 1.060691e-01 | | Pearson | 15 |
| Yb | D | | L | | 0.665777579 | | 0.95 | | 0.232969817 | | | 0.878444268 | | 3.217173961 | | 13 | | 6.740997e-03 | | Pearson | 15 |
| Hf | D | | L | | 0.394817279 | | 0.95 | | -0.147221057 | | | 0.754485303 | | 1.549408406 | | 13 | | 1.452776e-01 | | Pearson | 15 |
| Pt | D | | L | | 0.689502834 | | 0.95 | | 0.274028790 | | | 0.888087307 | | 3.432412298 | | 13 | | 4.456076e-03 | | Pearson | 15 |
| Hg | D | | L | | 0.541597816 | | 0.95 | | 0.040598691 | | | 0.824978315 | | 2.322949126 | | 13 | | 3.704554e-02 | | Pearson | 15 |
| Hg | D | | L | | 0.541597816 | | 0.95 | | 0.040598691 | | | 0.824978315 | | 2.322949126 | | 13 | | 3.704554e-02 | | Pearson | 15 |
| Tl | D | | L | | 0.110430034 | | 0.95 | | -0.425927170 | | | 0.589353614 | | 0.400611329 | | 13 | | 6.952123e-01 | | Pearson | 15 |
| Pb | D | | L | | 0.007684509 | | 0.95 | | -0.506572452 | | | 0.517908612 | | 0.027707709 | | 13 | | 9.783161e-01 | | Pearson | 15 |
| Bi | D | | L | | -0.066346820 | | 0.95 | | -0.559590844 | | | 0.461604584 | | -0.239745109 | | 13 | | 8.142666e-01 | | Pearson | 15 |
| Th | D | | L | | 0.413364262 | | 0.95 | | -0.125466193 | | | 0.763875573 | | 1.636791238 | | 13 | | 1.256409e-01 | | Pearson | 15 |
| U | D | | L | | 0.006138789 | | 0.95 | | -0.507720671 | | | 0.516776539 | | 0.022134134 | | 13 | | 9.826771e-01 | | Pearson | 15 |

| ELEMENT | PARAMETER1 | PARAMETER2 | R | CI | CI_LOW | CI_HIGH | T | DF_ERROR | P | METHOD | N_OBS |
| --- | --- | --- | --- | --- | --- | --- | --- | --- | --- | --- | --- |
| Be | E | L | -0.098369449 | 0.95 | -0.56645324 | 0.4177041 | -0.36985860 | 14 | 7.170227e-01 | Pearson | 16 |
| Na | E | L | -0.094905257 | 0.95 | -0.56407372 | 0.4205866 | -0.35671305 | 14 | 7.266257e-01 | Pearson | 16 |
| Mg | E | L | 0.373418693 | 0.95 | -0.15006440 | 0.7333726 | 1.50615573 | 14 | 1.542543e-01 | Pearson | 16 |
| Al | E | L | -0.013142574 | 0.95 | -0.50555430 | 0.4857272 | -0.04917926 | 14 | 9.614712e-01 | Pearson | 16 |
| P | E | L | 0.321056411 | 0.95 | -0.20770505 | 0.7046216 | 1.26843389 | 14 | 2.253299e-01 | Pearson | 16 |
| K | E | L | 0.173609209 | 0.95 | -0.35242551 | 0.6162782 | 0.65960249 | 14 | 5.202127e-01 | Pearson | 16 |
| Ca | E | L | 0.235165621 | 0.95 | -0.29491929 | 0.6545663 | 0.90529798 | 14 | 3.806236e-01 | Pearson | 16 |
| Ti | E | L | 0.386293362 | 0.95 | -0.13532505 | 0.7402499 | 1.56701546 | 14 | 1.394299e-01 | Pearson | 16 |
| V | E | L | 0.060202222 | 0.95 | -0.44889942 | 0.5397986 | 0.22566540 | 14 | 8.247227e-01 | Pearson | 16 |
| Cr | E | L | -0.083639687 | 0.95 | -0.55628124 | 0.4298891 | -0.31405147 | 14 | 7.581128e-01 | Pearson | 16 |
| Mn | E | L | -0.093050065 | 0.95 | -0.56279619 | 0.4221260 | -0.34967857 | 14 | 7.317843e-01 | Pearson | 16 |
| Fe | E | L | -0.181411640 | 0.95 | -0.62125000 | 0.3453499 | -0.69023307 | 14 | 5.013308e-01 | Pearson | 16 |
| Co | E | L | 0.459295232 | 0.95 | -0.04714350 | 0.7778936 | 1.93465831 | 14 | 7.350114e-02 | Pearson | 16 |
| Ni | E | L | -0.007837865 | 0.95 | -0.50159436 | 0.4897704 | -0.02932751 | 14 | 9.770174e-01 | Pearson | 16 |
| Cu | E | L | 0.425237277 | 0.95 | -0.08928963 | 0.7606115 | 1.75795382 | 14 | 1.005878e-01 | Pearson | 16 |
| Zn | E | L | 0.483672823 | 0.95 | -0.01582723 | 0.7899745 | 2.06768387 | 14 | 5.767277e-02 | Pearson | 16 |
| Ga | E | L | -0.017475153 | 0.95 | -0.50877322 | 0.4824091 | -0.06539602 | 14 | 9.487834e-01 | Pearson | 16 |
| Rb | E | L | 0.402891338 | 0.95 | -0.11597623 | 0.7490082 | 1.64707451 | 14 | 1.217963e-01 | Pearson | 16 |
| Sr | E | L | 0.405579173 | 0.95 | -0.11280544 | 0.7504152 | 1.66021754 | 14 | 1.190939e-01 | Pearson | 16 |
| Zr | E | L | 0.766954125 | 0.95 | 0.43762740 | 0.9148491 | 4.47198212 | 14 | 5.267142e-04 | Pearson | 16 |
| As | E | L | 0.363827053 | 0.95 | -0.16089604 | 0.7282007 | 1.46147623 | 14 | 1.659654e-01 | Pearson | 16 |
| Se | E | L | 0.916673966 | 0.95 | 0.77157032 | 0.9711078 | 8.58251234 | 14 | 5.986641e-07 | Pearson | 16 |
| Mo | E | L | 0.036625343 | 0.95 | -0.46756888 | 0.5228383 | 0.13713149 | 14 | 8.928795e-01 | Pearson | 16 |
| Pd | E | L | -0.003817687 | 0.95 | -0.49857948 | 0.4928203 | -0.01428458 | 14 | 9.888045e-01 | Pearson | 16 |
| Ag | E | L | -0.040902123 | 0.95 | -0.52594373 | 0.4642154 | -0.15316991 | 14 | 8.804501e-01 | Pearson | 16 |
| Cd | E | L | 0.774788861 | 0.95 | 0.45310694 | 0.9179427 | 4.58541983 | 14 | 4.240723e-04 | Pearson | 16 |
| Sn | E | L | 0.808263105 | 0.95 | 0.52150356 | 0.9309669 | 5.13609581 | 14 | 1.513081e-04 | Pearson | 16 |
| Sb | E | L | 0.047096836 | 0.95 | -0.45933213 | 0.5304189 | 0.17641599 | 14 | 8.624937e-01 | Pearson | 16 |
| Cs | E | L | 0.753326695 | 0.95 | 0.41116007 | 0.9094266 | 4.28602674 | 14 | 7.536516e-04 | Pearson | 16 |
| Ba | E | L | 0.290300454 | 0.95 | -0.23993191 | 0.6871260 | 1.13508672 | 14 | 2.753993e-01 | Pearson | 16 |
| La | E | L | 0.103452078 | 0.95 | -0.41345600 | 0.5699304 | 0.38917035 | 14 | 7.030053e-01 | Pearson | 16 |
| Ce | E | L | 0.904255680 | 0.95 | 0.74045523 | 0.9666608 | 7.92384779 | 14 | 1.530472e-06 | Pearson | 16 |
| Pr | E | L | 0.289640381 | 0.95 | -0.24061098 | 0.6867454 | 1.13226917 | 14 | 2.765422e-01 | Pearson | 16 |
| Nd | E | L | -0.243500305 | 0.95 | -0.65959020 | 0.2868262 | -0.93936895 | 14 | 3.634669e-01 | Pearson | 16 |
| Sm | E | L | 0.614941632 | 0.95 | 0.17152103 | 0.8511815 | 2.91780468 | 14 | 1.123944e-02 | Pearson | 16 |
| Eu | E | L | 0.718081228 | 0.95 | 0.34528097 | 0.8951514 | 3.86059059 | 14 | 1.731031e-03 | Pearson | 16 |
| Gd | E | L | 0.200134813 | 0.95 | -0.32812290 | 0.6330378 | 0.76429892 | 14 | 4.573785e-01 | Pearson | 16 |
| Tb | E | L | 0.578731321 | 0.95 | 0.11642643 | 0.8349158 | 2.65526155 | 14 | 1.883460e-02 | Pearson | 16 |
| Dy | E | L | 0.682240224 | 0.95 | 0.28185571 | 0.8802528 | 3.49146644 | 14 | 3.595676e-03 | Pearson | 16 |
| Ho | E | L | 0.632466238 | 0.95 | 0.19921962 | 0.8588938 | 3.05513666 | 14 | 8.562073e-03 | Pearson | 16 |
| Er | E | L | 0.308994124 | 0.95 | -0.22048252 | 0.6978151 | 1.21563871 | 14 | 2.442246e-01 | Pearson | 16 |
| Tm | E | L | 0.648885524 | 0.95 | 0.22581492 | 0.8660278 | 3.19089477 | 14 | 6.537860e-03 | Pearson | 16 |
| Yb | E | L | 0.526159017 | 0.95 | 0.04119928 | 0.8104761 | 2.31507376 | 14 | 3.629702e-02 | Pearson | 16 |
| Hf | E | L | 0.551207017 | 0.95 | 0.07636827 | 0.8222452 | 2.47184279 | 14 | 2.688869e-02 | Pearson | 16 |
| Pt | E | L | 0.581921550 | 0.95 | 0.12116876 | 0.8363671 | 2.67736201 | 14 | 1.803837e-02 | Pearson | 16 |
| Hg | E | L | 0.508014102 | 0.95 | 0.01645173 | 0.8018047 | 2.20678873 | 14 | 4.452980e-02 | Pearson | 16 |
| Hg | E | L | 0.508014102 | 0.95 | 0.01645173 | 0.8018047 | 2.20678873 | 14 | 4.452980e-02 | Pearson | 16 |
| Tl | E | L | -0.225744199 | 0.95 | -0.64884238 | 0.3039769 | -0.86703866 | 14 | 4.005377e-01 | Pearson | 16 |
| Pb | E | L | -0.063392281 | 0.95 | -0.54206385 | 0.4463387 | -0.23767022 | 14 | 8.155799e-01 | Pearson | 16 |
| Bi | E | L | -0.072034541 | 0.95 | -0.54816601 | 0.4393594 | -0.27023059 | 14 | 7.909256e-01 | Pearson | 16 |
| Th | E | L | -0.128968660 | 0.95 | -0.58713799 | 0.3917836 | -0.48662047 | 14 | 6.340570e-01 | Pearson | 16 |
| U | E | L | 0.044091970 | 0.95 | -0.46170467 | 0.5282515 | 0.16513765 | 14 | 8.711964e-01 | Pearson | 16 |
| ELEMENT | PARAMETER1 | PARAMETER2 | R | CI | CI_LOW | CI_HIGH | T | DF_ERROR | P | METHOD | N_OBS |
| Be | F | L | -0.092691150 | 0.95 | -0.56254877 | 0.4224235 | -0.3483181 | 14 | 7.327835e-01 | Pearson | 16 |
| Na | F | L | -0.219038679 | 0.95 | -0.64473911 | 0.3103657 | -0.8399653 | 14 | 4.150425e-01 | Pearson | 16 |
| Mg | F | L | 0.560106947 | 0.95 | 0.08915545 | 0.8263718 | 2.5297887 | 14 | 2.404082e-02 | Pearson | 16 |
| Al | F | L | 0.039999649 | 0.95 | -0.46492422 | 0.5252895 | 0.1497849 | 14 | 8.830708e-01 | Pearson | 16 |
| P | F | L | 0.589841939 | 0.95 | 0.13303418 | 0.8399548 | 2.7330468 | 14 | 1.617430e-02 | Pearson | 16 |
| K | F | L | 0.318416781 | 0.95 | -0.21051669 | 0.7031382 | 1.2568231 | 14 | 2.293830e-01 | Pearson | 16 |
| Ca | F | L | -0.243171499 | 0.95 | -0.65939270 | 0.2871469 | -0.9380207 | 14 | 3.641355e-01 | Pearson | 16 |
| Ti | F | L | -0.130240227 | 0.95 | -0.58798480 | 0.3906882 | -0.4915007 | 14 | 6.306883e-01 | Pearson | 16 |
| V | F | L | 0.042209462 | 0.95 | -0.46318735 | 0.5268904 | 0.1580742 | 14 | 8.766557e-01 | Pearson | 16 |
| Cr | F | L | 0.105554681 | 0.95 | -0.41169203 | 0.5713640 | 0.3971682 | 14 | 6.972324e-01 | Pearson | 16 |
| Mn | F | L | -0.201451935 | 0.95 | -0.63385955 | 0.3268977 | -0.7695409 | 14 | 4.543610e-01 | Pearson | 16 |
| Fe | F | L | 0.329480856 | 0.95 | -0.19867279 | 0.7093339 | 1.3057125 | 14 | 2.126997e-01 | Pearson | 16 |
| Co | F | L | 0.203836982 | 0.95 | -0.32467445 | 0.6353451 | 0.7790443 | 14 | 4.489224e-01 | Pearson | 16 |
| Ni | F | L | -0.009154143 | 0.95 | -0.50257889 | 0.4887691 | -0.0342531 | 14 | 9.731589e-01 | Pearson | 16 |
| Cu | F | L | 0.567151037 | 0.95 | 0.09938748 | 0.8296178 | 2.5765533 | 14 | 2.195596e-02 | Pearson | 16 |
| Zn | F | L | 0.508765778 | 0.95 | 0.01746511 | 0.8021664 | 2.2111932 | 14 | 4.416328e-02 | Pearson | 16 |
| Ga | F | L | -0.048088030 | 0.95 | -0.53113249 | 0.4585479 | -0.1801373 | 14 | 8.596262e-01 | Pearson | 16 |
| Rb | F | L | 0.405727654 | 0.95 | -0.11262997 | 0.7504928 | 1.6609451 | 14 | 1.189458e-01 | Pearson | 16 |
| Sr | F | L | -0.091752399 | 0.95 | -0.56190125 | 0.4232010 | -0.3447603 | 14 | 7.353990e-01 | Pearson | 16 |
| Zr | F | L | 0.837242221 | 0.95 | 0.58384913 | 0.9419955 | 5.7287656 | 14 | 5.215102e-05 | Pearson | 16 |
| As | F | L | 0.052835963 | 0.95 | -0.45478056 | 0.5345411 | 0.1979706 | 14 | 8.459138e-01 | Pearson | 16 |
| Se | F | L | 0.939432155 | 0.95 | 0.83045383 | 0.9791605 | 10.2558364 | 14 | 6.825417e-08 | Pearson | 16 |
| Mo | F | L | 0.704910869 | 0.95 | 0.32157173 | 0.8897223 | 3.7185256 | 14 | 2.291723e-03 | Pearson | 16 |
| Pd | F | L | -0.086072324 | 0.95 | -0.55797098 | 0.4278896 | -0.3232528 | 14 | 7.512816e-01 | Pearson | 16 |
| Ag | F | L | -0.096620215 | 0.95 | -0.56525268 | 0.4191609 | -0.3632191 | 14 | 7.218669e-01 | Pearson | 16 |
| Cd | F | L | 0.774854636 | 0.95 | 0.45323772 | 0.9179686 | 4.5863939 | 14 | 4.232863e-04 | Pearson | 16 |
| Sn | F | L | 0.892901208 | 0.95 | 0.71260710 | 0.9625615 | 7.4201241 | 14 | 3.251126e-06 | Pearson | 16 |
| Sb | F | L | 0.090207525 | 0.95 | -0.42447895 | 0.5608344 | 0.3389074 | 14 | 7.397091e-01 | Pearson | 16 |
| Cs | F | L | 0.704635557 | 0.95 | 0.32108119 | 0.8896082 | 3.7156406 | 14 | 2.304845e-03 | Pearson | 16 |
| Ba | F | L | 0.055580302 | 0.95 | -0.45259467 | 0.5365042 | 0.2082844 | 14 | 8.380066e-01 | Pearson | 16 |
| La | F | L | 0.472761478 | 0.95 | -0.02996652 | 0.7845964 | 2.0074111 | 14 | 6.441134e-02 | Pearson | 16 |
| Ce | F | L | 0.876906988 | 0.95 | 0.67432020 | 0.9567326 | 6.8262073 | 14 | 8.242550e-06 | Pearson | 16 |
| Pr | F | L | 0.375661985 | 0.95 | -0.14751284 | 0.7345763 | 1.5166854 | 14 | 1.515980e-01 | Pearson | 16 |
| Nd | F | L | -0.095482958 | 0.95 | -0.56447108 | 0.4201066 | -0.3589043 | 14 | 7.250216e-01 | Pearson | 16 |
| Sm | F | L | 0.434490132 | 0.95 | -0.07801882 | 0.7653542 | 1.8049911 | 14 | 9.262256e-02 | Pearson | 16 |
| Eu | F | L | 0.580220419 | 0.95 | 0.11863738 | 0.8355937 | 2.6655543 | 14 | 1.845962e-02 | Pearson | 16 |
| Gd | F | L | 0.783515436 | 0.95 | 0.47057997 | 0.9213681 | 4.7180188 | 14 | 3.297652e-04 | Pearson | 16 |
| Tb | F | L | 0.427951588 | 0.95 | -0.08599695 | 0.7620065 | 1.7716810 | 14 | 9.820281e-02 | Pearson | 16 |
| Dy | F | L | 0.530170074 | 0.95 | 0.04675139 | 0.8123763 | 2.3395861 | 14 | 3.464394e-02 | Pearson | 16 |
| Ho | F | L | 0.600733509 | 0.95 | 0.14956737 | 0.8448528 | 2.8116090 | 14 | 1.385991e-02 | Pearson | 16 |
| Er | F | L | 0.397033717 | 0.95 | -0.12284992 | 0.7459311 | 1.6186071 | 14 | 1.278323e-01 | Pearson | 16 |
| Tm | F | L | 0.564390534 | 0.95 | 0.09536584 | 0.8283478 | 2.5581293 | 14 | 2.275568e-02 | Pearson | 16 |
| Yb | F | L | 0.799977276 | 0.95 | 0.50422315 | 0.9277719 | 4.9884829 | 14 | 1.987161e-04 | Pearson | 16 |
| Hf | F | L | 0.730835712 | 0.95 | 0.36870425 | 0.9003595 | 4.0063338 | 14 | 1.299806e-03 | Pearson | 16 |
| Pt | F | L | 0.826016959 | 0.95 | 0.55933924 | 0.9377502 | 5.4833646 | 14 | 8.059365e-05 | Pearson | 16 |
| Hg | F | L | 0.247131087 | 0.95 | -0.28327685 | 0.6617672 | 0.9542796 | 14 | 3.561298e-01 | Pearson | 16 |
| Hg | F | L | 0.247131087 | 0.95 | -0.28327685 | 0.6617672 | 0.9542796 | 14 | 3.561298e-01 | Pearson | 16 |
| Tl | F | L | -0.055089812 | 0.95 | -0.53615369 | 0.4529858 | -0.2064407 | 14 | 8.394188e-01 | Pearson | 16 |
| Pb | F | L | 0.213896935 | 0.95 | -0.31523244 | 0.6415761 | 0.8192905 | 14 | 4.263485e-01 | Pearson | 16 |
| Bi | F | L | -0.051841958 | 0.95 | -0.53382878 | 0.4555708 | -0.1942360 | 14 | 8.487813e-01 | Pearson | 16 |
| Th | F | L | -0.182814533 | 0.95 | -0.62214019 | 0.3440713 | -0.6957546 | 14 | 4.979704e-01 | Pearson | 16 |
| U | F | L | 0.882068556 | 0.95 | 0.68655804 | 0.9586207 | 7.0054144 | 14 | 6.194659e-06 | Pearson | 16 |

| ELEMENT | PARAMETER1 | PARAMETER2 | | R | | | CI | | CI_LOW | | CI_HIGH | | T | | DF_ERROR | | P | | METHOD | N_OBS |
| --- | --- | --- | --- | --- | --- | --- | --- | --- | --- | --- | --- | --- | --- | --- | --- | --- | --- | --- | --- | --- |
| Be | G | L | | 0.704524078 | | | 0.95 | | 0.32088262 | | 0.8895620 | | 3.714473290 | | 14 | | 2.310176e-03 | | Pearson | 16 |
| Na | G | L | | 0.249115505 | | | 0.95 | | -0.28133077 | | 0.6629540 | | 0.962447190 | | 14 | | 3.521550e-01 | | Pearson | 16 |
| Mg | G | L | | 0.515353892 | | | 0.95 | | 0.02639033 | | 0.8053273 | | 2.250091497 | | 14 | | 4.104682e-02 | | Pearson | 16 |
| Al | G | L | | 0.258238134 | | | 0.95 | | -0.27232789 | | 0.6683837 | | 1.000162853 | | 14 | | 3.342059e-01 | | Pearson | 16 |
| P | G | L | | 0.331367110 | | | 0.95 | | -0.19663808 | | 0.7103844 | | 1.314106725 | | 14 | | 2.099354e-01 | | Pearson | 16 |
| K | G | L | | 0.189190771 | | | 0.95 | | -0.33823523 | | 0.6261719 | | 0.720906362 | | 14 | | 4.828324e-01 | | Pearson | 16 |
| Ca | G | L | | -0.167945448 | | | 0.95 | | -0.61264702 | | 0.3575243 | | -0.637448459 | | 14 | | 5.341196e-01 | | Pearson | 16 |
| Ti | G | L | | -0.030875796 | | | 0.95 | | -0.51864314 | | 0.4720545 | | -0.115581755 | | 14 | | 9.096255e-01 | | Pearson | 16 |
| V | G | L | | -0.109884810 | | | 0.95 | | -0.57430734 | | 0.4080470 | | -0.413656279 | | 14 | | 6.853927e-01 | | Pearson | 16 |
| Cr | G | L | | -0.028953881 | | | 0.95 | | -0.51723554 | | 0.4735481 | | -0.108380942 | | 14 | | 9.152315e-01 | | Pearson | 16 |
| Mn | G | L | | -0.321620778 | | | 0.95 | | -0.70493839 | | 0.2071028 | | -1.270920596 | | 14 | | 2.244692e-01 | | Pearson | 16 |
| Fe | G | L | | 0.139873611 | | | 0.95 | | -0.38234181 | | 0.5943678 | | 0.528555162 | | 14 | | 6.053925e-01 | | Pearson | 16 |
| Co | G | L | | 0.420568232 | | | 0.95 | | -0.09492735 | | 0.7582046 | | 1.734475951 | | 14 | | 1.047858e-01 | | Pearson | 16 |
| Ni | G | L | | 0.953746917 | | | 0.95 | | 0.86878221 | | 0.9841625 | | 11.871146656 | | 14 | | 1.074227e-08 | | Pearson | 16 |
| Cu | G | L | | -0.141413105 | | | 0.95 | | -0.59538257 | | 0.3810000 | | -0.534490660 | | 14 | | 6.013878e-01 | | Pearson | 16 |
| Zn | G | L | | 0.348011121 | | | 0.95 | | -0.17848479 | | 0.7195809 | | 1.388961824 | | 14 | | 1.865444e-01 | | Pearson | 16 |
| Ga | G | L | | 0.130280125 | | | 0.95 | | -0.39065385 | | 0.5880114 | | 0.491653846 | | 14 | | 6.305828e-01 | | Pearson | 16 |
| Rb | G | L | | 0.482842383 | | | 0.95 | | -0.01691041 | | 0.7895668 | | 2.063053283 | | 14 | | 5.816652e-02 | | Pearson | 16 |
| Sr | G | L | | 0.021379756 | | | 0.95 | | -0.47940635 | | 0.5116625 | | 0.080014010 | | 14 | | 9.373586e-01 | | Pearson | 16 |
| Zr | G | L | | 0.423380846 | | | 0.95 | | -0.09153516 | | 0.7596556 | | 1.748598629 | | 14 | | 1.022424e-01 | | Pearson | 16 |
| As | G | L | | 0.150422923 | | | 0.95 | | -0.37310300 | | 0.6012926 | | 0.569308785 | | 14 | | 5.781671e-01 | | Pearson | 16 |
| Se | G | L | | 0.903498079 | | | 0.95 | | 0.73857945 | | 0.9663882 | | 7.887639431 | | 14 | | 1.613941e-06 | | Pearson | 16 |
| Mo | G | L | | 0.001080941 | | | 0.95 | | -0.49488956 | | 0.4965202 | | 0.004044512 | | 14 | | 9.968300e-01 | | Pearson | 16 |
| Pd | G | L | | -0.065056702 | | | 0.95 | | -0.54324301 | | 0.4449994 | | -0.243936654 | | 14 | | 8.108183e-01 | | Pearson | 16 |
| Ag | G | L | | 0.642409335 | | | 0.95 | | 0.21524919 | | 0.8632245 | | 3.136476743 | | 14 | | 7.284834e-03 | | Pearson | 16 |
| Cd | G | L | | 0.733699446 | | | 0.95 | | 0.37402741 | | 0.9015223 | | 4.040259583 | | 14 | | 1.216222e-03 | | Pearson | 16 |
| Sn | G | L | | 0.644403750 | | | 0.95 | | 0.21849240 | | 0.8640893 | | 3.153111014 | | 14 | | 7.047920e-03 | | Pearson | 16 |
| Sb | G | L | | 0.105047140 | | | 0.95 | | -0.41211818 | | 0.5710182 | | 0.395237158 | | 14 | | 6.986245e-01 | | Pearson | 16 |
| Cs | G | L | | 0.806396766 | | | 0.95 | | 0.51759061 | | 0.9302489 | | 5.102111174 | | 14 | | 1.610669e-04 | | Pearson | 16 |
| Ba | G | L | | 0.254478780 | | | 0.95 | | -0.27604918 | | 0.6661514 | | 0.984586615 | | 14 | | 3.415380e-01 | | Pearson | 16 |
| La | G | L | | -0.358842969 | | | 0.95 | | -0.72549668 | | 0.1664750 | | -1.438472683 | | 14 | | 1.722789e-01 | | Pearson | 16 |
| Ce | G | L | | 0.682373008 | | | 0.95 | | 0.28208440 | | 0.8803087 | | 3.492738009 | | 14 | | 3.586600e-03 | | Pearson | 16 |
| Pr | G | L | | 0.398185452 | | | 0.95 | | -0.12150233 | | 0.7465373 | | 1.624185674 | | 14 | | 1.266295e-01 | | Pearson | 16 |
| Nd | G | L | | 0.123315152 | | | 0.95 | | -0.39663572 | | 0.5833608 | | 0.464951765 | | 14 | | 6.491151e-01 | | Pearson | 16 |
| Sm | G | L | | 0.612507875 | | | 0.95 | | 0.16772900 | | 0.8501023 | | 2.899301180 | | 14 | | 1.165814e-02 | | Pearson | 16 |
| Eu | G | L | | 0.619132589 | | | 0.95 | | 0.17808190 | | 0.8530352 | | 2.949983376 | | 14 | | 1.054624e-02 | | Pearson | 16 |
| Gd | G | L | | 0.666869026 | | | 0.95 | | 0.25568593 | | 0.8737412 | | 3.348469279 | | 14 | | 4.778497e-03 | | Pearson | 16 |
| Tb | G | L | | 0.665819440 | | | 0.95 | | 0.25392070 | | 0.8732939 | | 3.338996278 | | 14 | | 4.869416e-03 | | Pearson | 16 |
| Dy | G | L | | 0.704379018 | | | 0.95 | | 0.32062429 | | 0.8895019 | | 3.712955221 | | 14 | | 2.317127e-03 | | Pearson | 16 |
| Ho | G | L | | 0.133381661 | | | 0.95 | | -0.38797586 | | 0.5900726 | | 0.503567985 | | 14 | | 6.223954e-01 | | Pearson | 16 |
| Er | G | L | | 0.167060902 | | | 0.95 | | -0.35831779 | | 0.6120782 | | 0.633994448 | | 14 | | 5.363064e-01 | | Pearson | 16 |
| Tm | G | L | | 0.786005795 | | | 0.95 | | 0.47561178 | | 0.9223417 | | 4.757155288 | | 14 | | 3.062915e-04 | | Pearson | 16 |
| Yb | G | L | | 0.678778218 | | | 0.95 | | 0.27590913 | | 0.8787927 | | 3.458539651 | | 14 | | 3.838905e-03 | | Pearson | 16 |
| Hf | G | L | | 0.314432272 | | | 0.95 | | -0.21474435 | | 0.7008925 | | 1.239358194 | | 14 | | 2.355877e-01 | | Pearson | 16 |
| Pt | G | L | | 0.636025146 | | | 0.95 | | 0.20493053 | | 0.8604476 | | 3.083952117 | | 14 | | 8.086038e-03 | | Pearson | 16 |
| Hg | G | L | | 0.616318363 | | | 0.95 | | 0.17367195 | | 0.8517911 | | 2.928331001 | | 14 | | 1.100789e-02 | | Pearson | 16 |
| Hg | G | L | | 0.616318363 | | | 0.95 | | 0.17367195 | | 0.8517911 | | 2.928331001 | | 14 | | 1.100789e-02 | | Pearson | 16 |
| Tl | G | L | | 0.009298549 | | | 0.95 | | -0.48865918 | | 0.5026868 | | 0.034793489 | | 14 | | 9.727356e-01 | | Pearson | 16 |
| Pb | G | L | | -0.226572078 | | | 0.95 | | -0.64934728 | | 0.3031848 | | -0.870390106 | | 14 | | 3.987659e-01 | | Pearson | 16 |
| Bi | G | L | | 0.922281197 | | | 0.95 | | 0.78585099 | | 0.9731034 | | 8.928034563 | | 14 | | 3.732558e-07 | | Pearson | 16 |
| Th | G | L | | 0.578533596 | | | 0.95 | | 0.11613320 | | 0.8348258 | | 2.653897870 | | 14 | | 1.888483e-02 | | Pearson | 16 |
| U | G | L | | 0.469452227 | | | 0.95 | | -0.03421536 | | 0.7829559 | | 1.989369419 | | 14 | | 6.656443e-02 | | Pearson | 16 |
| ELEMENT | PARAMETER1 | | PARAMETER2 | | R | CI | | CI_LOW | | CI_HIGH | | T | | DF_ERROR | | P | | METHOD | | N_OBS |
| Be | H | | L | | 0.73872669 | 0.95 | | 0.36433712 | | 0.9075518 | | 3.95177570 | | 13 | | 1.655981e-03 | | Pearson | | 15 |
| Na | H | | L | | -0.14432378 | 0.95 | | -0.61138583 | | 0.3973131 | | -0.52587241 | | 13 | | 6.078314e-01 | | Pearson | | 15 |
| Mg | H | | L | | 0.21484653 | 0.95 | | -0.33419737 | | 0.6550193 | | 0.79316226 | | 13 | | 4.419197e-01 | | Pearson | | 15 |
| Al | H | | L | | 0.28337765 | 0.95 | | -0.26775329 | | 0.6947831 | | 1.06540533 | | 13 | | 3.060809e-01 | | Pearson | | 15 |
| P | H | | L | | 0.11240861 | 0.95 | | -0.42428578 | | 0.5906596 | | 0.40788013 | | 13 | | 6.899994e-01 | | Pearson | | 15 |
| K | H | | L | | -0.09185523 | 0.95 | | -0.57696934 | | 0.4411663 | | -0.33259481 | | 13 | | 7.447424e-01 | | Pearson | | 15 |
| Ca | H | | L | | 0.02593599 | 0.95 | | -0.49287522 | | 0.5311421 | | 0.09354501 | | 13 | | 9.268970e-01 | | Pearson | | 15 |
| Ti | H | | L | | -0.09882403 | 0.95 | | -0.58164191 | | 0.4354847 | | -0.35806788 | | 13 | | 7.260396e-01 | | Pearson | | 15 |
| V | H | | L | | -0.24722645 | 0.95 | | -0.67411593 | | 0.3034692 | | -0.91994480 | | 13 | | 3.743570e-01 | | Pearson | | 15 |
| Cr | H | | L | | 0.56524101 | 0.95 | | 0.07457008 | | 0.8355645 | | 2.47053180 | | 13 | | 2.810656e-02 | | Pearson | | 15 |
| Mn | H | | L | | -0.43164766 | 0.95 | | -0.77298937 | | 0.1035010 | | -1.72533793 | | 13 | | 1.081420e-01 | | Pearson | | 15 |
| Fe | H | | L | | 0.05682012 | 0.95 | | -0.46909664 | | 0.5529873 | | 0.20519938 | | 13 | | 8.405945e-01 | | Pearson | | 15 |
| Co | H | | L | | 0.27944786 | 0.95 | | -0.27171055 | | 0.6925689 | | 1.04936957 | | 13 | | 3.131257e-01 | | Pearson | | 15 |
| Ni | H | | L | | -0.11981948 | 0.95 | | -0.59552929 | | 0.4181063 | | -0.43515024 | | 13 | | 6.705904e-01 | | Pearson | | 15 |
| Cu | H | | L | | 0.28332454 | 0.95 | | -0.26780689 | | 0.6947532 | | 1.06518823 | | 13 | | 3.061755e-01 | | Pearson | | 15 |
| Zn | H | | L | | 0.21600886 | 0.95 | | -0.33311417 | | 0.6557147 | | 0.79766272 | | 13 | | 4.393939e-01 | | Pearson | | 15 |
| Ga | H | | L | | 0.40922327 | 0.95 | | -0.13036876 | | 0.7617918 | | 1.61707588 | | 13 | | 1.298580e-01 | | Pearson | | 15 |
| Rb | H | | L | | 0.25727094 | 0.95 | | -0.29369854 | | 0.6799261 | | 0.95991495 | | 13 | | 3.546123e-01 | | Pearson | | 15 |
| Sr | H | | L | | 0.20474411 | 0.95 | | -0.34355136 | | 0.6489439 | | 0.75419257 | | 13 | | 4.641766e-01 | | Pearson | | 15 |
| Zr | H | | L | | 0.59874846 | 0.95 | | 0.12474785 | | 0.8502313 | | 2.69536546 | | 13 | | 1.835544e-02 | | Pearson | | 15 |
| As | H | | L | | 0.36538032 | 0.95 | | -0.18070526 | | 0.7392729 | | 1.41525026 | | 13 | | 1.805021e-01 | | Pearson | | 15 |
| Se | H | | L | | 0.90029207 | 0.95 | | 0.72015454 | | 0.9667180 | | 7.45727336 | | 13 | | 4.783685e-06 | | Pearson | | 15 |
| Mo | H | | L | | 0.12332075 | 0.95 | | -0.41516944 | | 0.5978179 | | 0.44805938 | | 13 | | 6.614865e-01 | | Pearson | | 15 |
| Pd | H | | L | | -0.07836919 | 0.95 | | -0.56783591 | | 0.4520411 | | -0.28343587 | | 13 | | 7.813062e-01 | | Pearson | | 15 |
| Ag | H | | L | | 0.97910599 | 0.95 | | 0.93660781 | | 0.9932132 | | 17.36025098 | | 13 | | 2.247728e-10 | | Pearson | | 15 |
| Cd | H | | L | | 0.74666857 | 0.95 | | 0.37959862 | | 0.9106257 | | 4.04715429 | | 13 | | 1.383752e-03 | | Pearson | | 15 |
| Sn | H | | L | | 0.80124008 | 0.95 | | 0.49016206 | | 0.9312681 | | 4.82818763 | | 13 | | 3.297643e-04 | | Pearson | | 15 |
| Sb | H | | L | | 0.13154369 | 0.95 | | -0.40822755 | | 0.6031625 | | 0.47844501 | | 13 | | 6.402797e-01 | | Pearson | | 15 |
| Cs | H | | L | | 0.77456915 | 0.95 | | 0.43484568 | | 0.9212827 | | 4.41548829 | | 13 | | 6.974884e-04 | | Pearson | | 15 |
| Ba | H | | L | | 0.02700643 | 0.95 | | -0.49206382 | | 0.5319106 | | 0.09740860 | | 13 | | 9.238877e-01 | | Pearson | | 15 |
| La | H | | L | | -0.26470167 | 0.95 | | -0.68419045 | | 0.2863955 | | -0.98969760 | | 13 | | 3.403860e-01 | | Pearson | | 15 |
| Ce | H | | L | | 0.88222478 | 0.95 | | 0.67502800 | | 0.9604366 | | 6.75597797 | | 13 | | 1.349983e-05 | | Pearson | | 15 |
| Pr | H | | L | | 0.41394939 | 0.95 | | -0.12477132 | | 0.7641694 | | 1.63958688 | | 13 | | 1.250527e-01 | | Pearson | | 15 |
| Nd | H | | L | | 0.13841086 | 0.95 | | -0.40238197 | | 0.6075936 | | 0.50389751 | | 13 | | 6.227643e-01 | | Pearson | | 15 |
| Sm | H | | L | | 0.73802290 | 0.95 | | 0.36299431 | | 0.9072785 | | 3.94350238 | | 13 | | 1.682045e-03 | | Pearson | | 15 |
| Eu | H | | L | | 0.76927248 | 0.95 | | 0.42415710 | | 0.9192764 | | 4.34115019 | | 13 | | 7.999723e-04 | | Pearson | | 15 |
| Gd | H | | L | | 0.09490385 | 0.95 | | -0.43868603 | | 0.5790174 | | 0.34373216 | | 13 | | 7.365436e-01 | | Pearson | | 15 |
| Tb | H | | L | | 0.74821693 | 0.95 | | 0.38259748 | | 0.9112229 | | 4.06619582 | | 13 | | 1.335150e-03 | | Pearson | | 15 |
| Dy | H | | L | | 0.75739798 | 0.95 | | 0.40053900 | | 0.9147499 | | 4.18228433 | | 13 | | 1.074469e-03 | | Pearson | | 15 |
| Ho | H | | L | | 0.66068827 | 0.95 | | 0.22435877 | | 0.8763529 | | 3.17339973 | | 13 | | 7.333700e-03 | | Pearson | | 15 |
| Er | H | | L | | -0.15828200 | 0.95 | | -0.62025346 | | 0.3852148 | | -0.57797992 | | 13 | | 5.731537e-01 | | Pearson | | 15 |
| Tm | H | | L | | 0.58915854 | 0.95 | | 0.11013484 | | 0.8460731 | | 2.62895476 | | 13 | | 2.082874e-02 | | Pearson | | 15 |
| Yb | H | | L | | 0.55436515 | 0.95 | | 0.05880052 | | 0.8307197 | | 2.40160457 | | 13 | | 3.198878e-02 | | Pearson | | 15 |
| Hf | H | | L | | 0.20582786 | 0.95 | | -0.34255309 | | 0.6495984 | | 0.75836075 | | 13 | | 4.617630e-01 | | Pearson | | 15 |
| Pt | H | | L | | 0.85743237 | 0.95 | | 0.61552861 | | 0.9516856 | | 6.00764931 | | 13 | | 4.390974e-05 | | Pearson | | 15 |
| Hg | H | | L | | 0.47199633 | 0.95 | | -0.05310707 | | 0.7926157 | | 1.93036069 | | 13 | | 7.566906e-02 | | Pearson | | 15 |
| Hg | H | | L | | 0.47199633 | 0.95 | | -0.05310707 | | 0.7926157 | | 1.93036069 | | 13 | | 7.566906e-02 | | Pearson | | 15 |
| Tl | H | | L | | 0.34475651 | 0.95 | | -0.20343399 | | 0.7283826 | | 1.32422250 | | 13 | | 2.082403e-01 | | Pearson | | 15 |
| Pb | H | | L | | 0.16545197 | 0.95 | | -0.37892677 | | 0.6247631 | | 0.60488213 | | 13 | | 5.556690e-01 | | Pearson | | 15 |
| Bi | H | | L | | 0.99998377 | 0.95 | | 0.99994968 | | 0.9999948 | | 632.86479257 | | 13 | | 1.445869e-30 | | Pearson | | 15 |
| Th | H | | L | | 0.47458640 | 0.95 | | -0.04977816 | | 0.7938532 | | 1.94402209 | | 13 | | 7.385727e-02 | | Pearson | | 15 |
| U | H | | L | | -0.04773861 | 0.95 | | -0.54663368 | | 0.4761688 | | -0.17232047 | | 13 | | 8.658394e-01 | | Pearson | | 15 |

| ELEMENT | | PARAMETER1 | | PARAMETER2 | | R | CI | | CI_LOW | | | CI_HIGH | | T | | DF_ERROR | | P | METHOD | | N_OBS | |
| --- | --- | --- | --- | --- | --- | --- | --- | --- | --- | --- | --- | --- | --- | --- | --- | --- | --- | --- | --- | --- | --- | --- |
| Be | | I | | L | | -0.02553410 | 0.95 | | -0.59077842 | | | 0.5565228 | | -0.08077226 | | 10 | | 9.372168e-01 | Pearson | | 12 | |
| Na | | I | | L | | -0.13147253 | 0.95 | | -0.65588604 | | | 0.4785357 | | -0.41939305 | | 10 | | 6.837959e-01 | Pearson | | 12 | |
| Mg | | I | | L | | 0.63402375 | 0.95 | | 0.09451193 | | | 0.8856620 | | 2.59268584 | | 10 | | 2.682605e-02 | Pearson | | 12 | |
| Al | | I | | L | | 0.06058510 | 0.95 | | -0.53180740 | | | 0.6131670 | | 0.19193951 | | 10 | | 8.516311e-01 | Pearson | | 12 | |
| P | | I | | L | | -0.01115964 | 0.95 | | -0.58133806 | | | 0.5663693 | | -0.03529208 | | 10 | | 9.725414e-01 | Pearson | | 12 | |
| K | | I | | L | | -0.06012754 | 0.95 | | -0.61288032 | | | 0.5321367 | | -0.19048464 | | 10 | | 8.527408e-01 | Pearson | | 12 | |
| Ca | | I | | L | | -0.21670060 | 0.95 | | -0.70315453 | | | 0.4079336 | | -0.70194707 | | 10 | | 4.987237e-01 | Pearson | | 12 | |
| Ti | | I | | L | | 0.42994252 | 0.95 | | -0.19111594 | | | 0.8051722 | | 1.50588493 | | 10 | | 1.630133e-01 | Pearson | | 12 | |
| V | | I | | L | | -0.31565891 | 0.95 | | -0.75312630 | | | 0.3153752 | | -1.05198631 | | 10 | | 3.175621e-01 | Pearson | | 12 | |
| Cr | | I | | L | | -0.13683277 | 0.95 | | -0.65898523 | | | 0.4743162 | | -0.43681178 | | 10 | | 6.715291e-01 | Pearson | | 12 | |
| Mn | | I | | L | | 0.76993358 | 0.95 | | 0.35122749 | | | 0.9320111 | | 3.81547098 | | 10 | | 3.398644e-03 | Pearson | | 12 | |
| Fe | | I | | L | | -0.01222665 | 0.95 | | -0.58204412 | | | 0.5656440 | | -0.03866694 | | 10 | | 9.699170e-01 | Pearson | | 12 | |
| Co | | I | | L | | 0.72930789 | 0.95 | | 0.26727412 | | | 0.9186906 | | 3.37083079 | | 10 | | 7.111475e-03 | Pearson | | 12 | |
| Ni | | I | | L | | -0.12457717 | 0.95 | | -0.65187309 | | | 0.4839225 | | -0.39704056 | | 10 | | 6.996793e-01 | Pearson | | 12 | |
| Cu | | I | | L | | 0.67597415 | 0.95 | | 0.16676956 | | | 0.9005241 | | 2.90072529 | | 10 | | 1.581394e-02 | Pearson | | 12 | |
| Zn | | I | | L | | 0.67459246 | 0.95 | | 0.16429925 | | | 0.9000428 | | 2.88983545 | | 10 | | 1.611171e-02 | Pearson | | 12 | |
| Ga | | I | | L | | -0.12689504 | 0.95 | | -0.65322535 | | | 0.4821169 | | -0.40454765 | | 10 | | 6.943274e-01 | Pearson | | 12 | |
| Rb | | I | | L | | 0.39243774 | 0.95 | | -0.23421362 | | | 0.7887064 | | 1.34923464 | | 10 | | 2.070164e-01 | Pearson | | 12 | |
| Sr | | I | | L | | -0.06202003 | 0.95 | | -0.61406499 | | | 0.5307736 | | -0.19650283 | | 10 | | 8.481527e-01 | Pearson | | 12 | |
| Zr | | I | | L | | 0.29899897 | 0.95 | | -0.33184611 | | | 0.7450526 | | 0.99084554 | | 10 | | 3.451301e-01 | Pearson | | 12 | |
| As | | I | | L | | -0.23765066 | 0.95 | | -0.71415063 | | | 0.3893542 | | -0.77368284 | | 10 | | 4.570184e-01 | Pearson | | 12 | |
| Se | | I | | L | | 0.87071007 | 0.95 | | 0.59326322 | | | 0.9632659 | | 5.59871001 | | 10 | | 2.280967e-04 | Pearson | | 12 | |
| Mo | | I | | L | | 0.38610437 | 0.95 | | -0.24125623 | | | 0.7858686 | | 1.32360829 | | 10 | | 2.150939e-01 | Pearson | | 12 | |
| Pd | | I | | L | | 0.10091671 | 0.95 | | -0.50206249 | | | 0.6378750 | | 0.32076420 | | 10 | | 7.549918e-01 | Pearson | | 12 | |
| Ag | | I | | L | | 0.35676177 | 0.95 | | -0.27304470 | | | 0.7724975 | | 1.20764860 | | 10 | | 2.549687e-01 | Pearson | | 12 | |
| Cd | | I | | L | | 0.79674814 | 0.95 | | 0.41059161 | | | 0.9405696 | | 4.16927165 | | 10 | | 1.920190e-03 | Pearson | | 12 | |
| Sn | | I | | L | | 0.60698710 | 0.95 | | 0.05077192 | | | 0.8758022 | | 2.41529320 | | 10 | | 3.635296e-02 | Pearson | | 12 | |
| Sb | | I | | L | | -0.13824871 | 0.95 | | -0.65980096 | | | 0.4731969 | | -0.44141953 | | 10 | | 6.683008e-01 | Pearson | | 12 | |
| Cs | | I | | L | | 0.75382352 | 0.95 | | 0.31711036 | | | 0.9267808 | | 3.62788457 | | 10 | | 4.628768e-03 | Pearson | | 12 | |
| Ba | | I | | L | | -0.13180786 | 0.95 | | -0.65608044 | | | 0.4782726 | | -0.42048162 | | 10 | | 6.830265e-01 | Pearson | | 12 | |
| La | | I | | L | | 0.12461843 | 0.95 | | -0.48389041 | | | 0.6518972 | | 0.39717414 | | 10 | | 6.995839e-01 | Pearson | | 12 | |
| Ce | | I | | L | | 0.49433704 | 0.95 | | -0.11107730 | | | 0.8321555 | | 1.79832548 | | 10 | | 1.023310e-01 | Pearson | | 12 | |
| Pr | | I | | L | | 0.60664977 | 0.95 | | 0.05023935 | | | 0.8756778 | | 2.41316892 | | 10 | | 3.648521e-02 | Pearson | | 12 | |
| Nd | | I | | L | | 0.06897151 | 0.95 | | -0.52574042 | | | 0.6183953 | | 0.21862768 | | 10 | | 8.313374e-01 | Pearson | | 12 | |
| Sm | | I | | L | | 0.45640050 | 0.95 | | -0.15920029 | | | 0.8164502 | | 1.62205722 | | 10 | | 1.358568e-01 | Pearson | | 12 | |
| Eu | | I | | L | | 0.59460049 | 0.95 | | 0.03142106 | | | 0.8712089 | | 2.33860947 | | 10 | | 4.143767e-02 | Pearson | | 12 | |
| Gd | | I | | L | | 0.41583543 | 0.95 | | -0.20761267 | | | 0.7990459 | | 1.44593075 | | 10 | | 1.787992e-01 | Pearson | | 12 | |
| Tb | | I | | L | | 0.46588669 | 0.95 | | -0.14743512 | | | 0.8204279 | | 1.66499679 | | 10 | | 1.268852e-01 | Pearson | | 12 | |
| Dy | | I | | L | | 0.42121404 | 0.95 | | -0.20136451 | | | 0.8013911 | | 1.46863578 | | 10 | | 1.726722e-01 | Pearson | | 12 | |
| Ho | | I | | L | | 0.26212215 | 0.95 | | -0.36698603 | | | 0.7267040 | | 0.85893598 | | 10 | | 4.104924e-01 | Pearson | | 12 | |
| Er | | I | | L | | -0.25134027 | 0.95 | | -0.72121109 | | | 0.3769317 | | -0.82116822 | | 10 | | 4.306918e-01 | Pearson | | 12 | |
| Tm | | I | | L | | 0.38662292 | 0.95 | | -0.24068209 | | | 0.7861016 | | 1.32569811 | | 10 | | 2.144255e-01 | Pearson | | 12 | |
| Yb | | I | | L | | 0.54108683 | 0.95 | | -0.04759426 | | | 0.8507916 | | 2.03464267 | | 10 | | 6.926505e-02 | Pearson | | 12 | |
| Hf | | I | | L | | -0.04772031 | 0.95 | | -0.60505152 | | | 0.5409975 | | -0.15107699 | | 10 | | 8.829191e-01 | Pearson | | 12 | |
| Pt | | I | | L | | 0.43018493 | 0.95 | | -0.19082937 | | | 0.8052767 | | 1.50692671 | | 10 | | 1.627500e-01 | Pearson | | 12 | |
| Hg | | I | | L | | 0.39275818 | 0.95 | | -0.23385554 | | | 0.7888495 | | 1.35053716 | | 10 | | 2.066127e-01 | Pearson | | 12 | |
| Hg | | I | | L | | 0.39275818 | 0.95 | | -0.23385554 | | | 0.7888495 | | 1.35053716 | | 10 | | 2.066127e-01 | Pearson | | 12 | |
| Tl | | I | | L | | 0.36829977 | 0.95 | | -0.26070690 | | | 0.7777996 | | 1.25272374 | | 10 | | 2.388083e-01 | Pearson | | 12 | |
| Pb | | I | | L | | -0.10017881 | 0.95 | | -0.63743265 | | | 0.5026198 | | -0.31839493 | | 10 | | 7.567354e-01 | Pearson | | 12 | |
| Bi | | I | | L | | 0.98681228 | 0.95 | | 0.95213798 | | | 0.9964125 | | 19.27841492 | | 10 | | 3.072762e-09 | Pearson | | 12 | |
| Th | | I | | L | | 0.20542817 | 0.95 | | -0.41772088 | | | 0.6971401 | | 0.66377784 | | 10 | | 5.218411e-01 | Pearson | | 12 | |
| U | | I | | L | | -0.07775035 | 0.95 | | -0.62381662 | | | 0.5193241 | | -0.24661474 | | 10 | | 8.101951e-01 | Pearson | | 12 | |
| ELEMENT | PARAMETER1 | | PARAMETER2 | | R | | | CI | | CI_LOW | CI_HIGH | | T | | DF_ERROR | | P | | | METHOD | | N_OBS |
| Be | J | | L | | -0.145548038 | | | 0.95 | | -0.59810101 | 0.3773852 | | -0.55045256 | | 14 | | 0.5906845307 | | | Pearson | | 16 |
| Na | J | | L | | 0.260562363 | | | 0.95 | | -0.27001923 | 0.6697600 | | 1.00981719 | | 14 | | 0.3297182325 | | | Pearson | | 16 |
| Mg | J | | L | | 0.467426134 | | | 0.95 | | -0.03680776 | 0.7819494 | | 1.97837656 | | 14 | | 0.0679083437 | | | Pearson | | 16 |
| Al | J | | L | | 0.442592718 | | | 0.95 | | -0.06804037 | 0.7694780 | | 1.84675845 | | 14 | | 0.0860256020 | | | Pearson | | 16 |
| P | J | | L | | 0.263680473 | | | 0.95 | | -0.26691239 | 0.6716022 | | 1.02279875 | | 14 | | 0.3237525183 | | | Pearson | | 16 |
| K | J | | L | | -0.022527217 | | | 0.95 | | -0.51250943 | 0.4785217 | | -0.08431052 | | 14 | | 0.9340032963 | | | Pearson | | 16 |
| Ca | J | | L | | 0.474958409 | | | 0.95 | | -0.02713574 | 0.7856830 | | 2.01944885 | | 14 | | 0.0630103944 | | | Pearson | | 16 |
| Ti | J | | L | | 0.587240617 | | | 0.95 | | 0.12912268 | 0.8387788 | | 2.71462749 | | 14 | | 0.0167691531 | | | Pearson | | 16 |
| V | J | | L | | 0.227909009 | | | 0.95 | | -0.30190416 | 0.6501618 | | 0.87580652 | | 14 | | 0.3959135618 | | | Pearson | | 16 |
| Cr | J | | L | | -0.060461370 | | | 0.95 | | -0.53998286 | 0.4486917 | | -0.22664036 | | 14 | | 0.8239792047 | | | Pearson | | 16 |
| Mn | J | | L | | -0.183825659 | | | 0.95 | | -0.62278108 | 0.3431485 | | -0.69973695 | | 14 | | 0.4955549832 | | | Pearson | | 16 |
| Fe | J | | L | | -0.098494462 | | | 0.95 | | -0.56653896 | 0.4175999 | | -0.37033324 | | 14 | | 0.7166769387 | | | Pearson | | 16 |
| Co | J | | L | | 0.207780506 | | | 0.95 | | -0.32098568 | 0.6377944 | | 0.79478937 | | 14 | | 0.4400025207 | | | Pearson | | 16 |
| Ni | J | | L | | 0.019686486 | | | 0.95 | | -0.48070995 | 0.5104109 | | 0.07367436 | | 14 | | 0.9423117975 | | | Pearson | | 16 |
| Cu | J | | L | | -0.052391676 | | | 0.95 | | -0.53422279 | 0.4551339 | | -0.19630130 | | 14 | | 0.8471952814 | | | Pearson | | 16 |
| Zn | J | | L | | -0.051485647 | | | 0.95 | | -0.53357327 | 0.4558539 | | -0.19289748 | | 14 | | 0.8498096011 | | | Pearson | | 16 |
| Ga | J | | L | | 0.005297854 | | | 0.95 | | -0.49169876 | 0.4996909 | | 0.01982303 | | 14 | | 0.9844642949 | | | Pearson | | 16 |
| Rb | J | | L | | 0.412888679 | | | 0.95 | | -0.10412881 | 0.7542258 | | 1.69622240 | | 14 | | 0.1119563136 | | | Pearson | | 16 |
| Sr | J | | L | | 0.187597319 | | | 0.95 | | -0.33969750 | 0.6251665 | | 0.71461210 | | 14 | | 0.4865945312 | | | Pearson | | 16 |
| Zr | J | | L | | 0.654715869 | | | 0.95 | | 0.23541288 | 0.8685399 | | 3.24090917 | | 14 | | 0.0059187828 | | | Pearson | | 16 |
| As | J | | L | | -0.158914456 | | | 0.95 | | -0.60681786 | 0.3655901 | | -0.60225671 | | 14 | | 0.5566325462 | | | Pearson | | 16 |
| Se | J | | L | | 0.824269920 | | | 0.95 | | 0.55556613 | 0.9370864 | | 5.44710652 | | 14 | | 0.0000860071 | | | Pearson | | 16 |
| Mo | J | | L | | -0.078572705 | | | 0.95 | | -0.55274906 | 0.4340379 | | -0.29490387 | | 14 | | 0.7723952601 | | | Pearson | | 16 |
| Pd | J | | L | | -0.045607073 | | | 0.95 | | -0.52934514 | 0.4605093 | | -0.17082379 | | 14 | | 0.8668065761 | | | Pearson | | 16 |
| Ag | J | | L | | 0.086840539 | | | 0.95 | | -0.42725706 | 0.5585038 | | 0.32615970 | | 14 | | 0.7491278790 | | | Pearson | | 16 |
| Cd | J | | L | | 0.752871165 | | | 0.95 | | 0.41028519 | 0.9092444 | | 4.28004143 | | 14 | | 0.0007624367 | | | Pearson | | 16 |
| Sn | J | | L | | 0.811017294 | | | 0.95 | | 0.52730010 | 0.9320248 | | 5.18706635 | | 14 | | 0.0001378079 | | | Pearson | | 16 |
| Sb | J | | L | | 0.247595700 | | | 0.95 | | -0.28282160 | 0.6620452 | | 0.95619077 | | 14 | | 0.3551969656 | | | Pearson | | 16 |
| Cs | J | | L | | 0.593885457 | | | 0.95 | | 0.13914263 | 0.8417779 | | 2.76193773 | | 14 | | 0.0152824248 | | | Pearson | | 16 |
| Ba | J | | L | | -0.225446836 | | | 0.95 | | -0.64866093 | 0.3042613 | | -0.86583534 | | 14 | | 0.4011751750 | | | Pearson | | 16 |
| La | J | | L | | 0.163980705 | | | 0.95 | | -0.36107501 | 0.6100939 | | 0.62197901 | | 14 | | 0.5439526201 | | | Pearson | | 16 |
| Ce | J | | L | | 0.709218916 | | | 0.95 | | 0.32927452 | 0.8915039 | | 3.76411088 | | 14 | | 0.0020941452 | | | Pearson | | 16 |
| Pr | J | | L | | 0.516488163 | | | 0.95 | | 0.02793489 | 0.8058699 | | 2.25684287 | | 14 | | 0.0405273847 | | | Pearson | | 16 |
| Nd | J | | L | | 0.423827923 | | | 0.95 | | -0.09099486 | 0.7598860 | | 1.75084914 | | 14 | | 0.1018422208 | | | Pearson | | 16 |
| Sm | J | | L | | 0.517522106 | | | 0.95 | | 0.02934486 | 0.8063640 | | 2.26301118 | | 14 | | 0.0400582148 | | | Pearson | | 16 |
| Eu | J | | L | | 0.557775760 | | | 0.95 | | 0.08579099 | 0.8252937 | | 2.51448999 | | 14 | | 0.0247632456 | | | Pearson | | 16 |
| Gd | J | | L | | 0.675303117 | | | 0.95 | | 0.26997092 | 0.8773233 | | 3.42591839 | | 14 | | 0.0040961858 | | | Pearson | | 16 |
| Tb | J | | L | | 0.709852100 | | | 0.95 | | 0.33041093 | 0.8917653 | | 3.77088163 | | 14 | | 0.0020663088 | | | Pearson | | 16 |
| Dy | J | | L | | 0.819892528 | | | 0.95 | | 0.54616055 | 0.9354198 | | 5.35836397 | | 14 | | 0.0001009166 | | | Pearson | | 16 |
| Ho | J | | L | | 0.686124134 | | | 0.95 | | 0.28856379 | 0.8818863 | | 3.52892947 | | 14 | | 0.0033377310 | | | Pearson | | 16 |
| Er | J | | L | | 0.342475877 | | | 0.95 | | -0.18456201 | 0.7165368 | | 1.36390749 | | 14 | | 0.1941245282 | | | Pearson | | 16 |
| Tm | J | | L | | 0.719609558 | | | 0.95 | | 0.34806343 | 0.8957781 | | 3.87761255 | | 14 | | 0.0016739388 | | | Pearson | | 16 |
| Yb | J | | L | | 0.703648148 | | | 0.95 | | 0.31932356 | 0.8891990 | | 3.70532060 | | 14 | | 0.0023524105 | | | Pearson | | 16 |
| Hf | J | | L | | 0.540009266 | | | 0.95 | | 0.06049851 | 0.8170123 | | 2.40065096 | | 14 | | 0.0308304655 | | | Pearson | | 16 |
| Pt | J | | L | | 0.610431959 | | | 0.95 | | 0.16450488 | 0.8491802 | | 2.88362266 | | 14 | | 0.0120248757 | | | Pearson | | 16 |
| Hg | J | | L | | 0.552609461 | | | 0.95 | | 0.07837295 | 0.8228973 | | 2.48089179 | | 14 | | 0.0264236006 | | | Pearson | | 16 |
| Hg | J | | L | | 0.552609461 | | | 0.95 | | 0.07837295 | 0.8228973 | | 2.48089179 | | 14 | | 0.0264236006 | | | Pearson | | 16 |
| Tl | J | | L | | -0.213536596 | | | 0.95 | | -0.64135383 | 0.3155725 | | -0.81784431 | | 14 | | 0.4271467411 | | | Pearson | | 16 |
| Pb | J | | L | | -0.084983755 | | | 0.95 | | -0.55721533 | 0.4287850 | | -0.31913462 | | 14 | | 0.7543363684 | | | Pearson | | 16 |
| Bi | J | | L | | -0.050260964 | | | 0.95 | | -0.53269441 | 0.4568260 | | -0.18829729 | | 14 | | 0.8533457112 | | | Pearson | | 16 |
| Th | J | | L | | 0.335911147 | | | 0.95 | | -0.19171765 | 0.7129081 | | 1.33440159 | | 14 | | 0.2033711107 | | | Pearson | | 16 |
| U | J | | L | | 0.143757186 | | | 0.95 | | -0.37895277 | 0.5969249 | | 0.54353585 | | 14 | | 0.5953105975 | | | Pearson | | 16 |

| ELEMENT | PARAMETER1 | PARAMETER2 | R | CI | CI_LOW | CI_HIGH | T | DF_ERROR | P | METHOD | N_OBS |
| --- | --- | --- | --- | --- | --- | --- | --- | --- | --- | --- | --- |
| Be | K | L | 0.029147995 | 0.95 | -0.47339737 | 0.5173778 | 0.10910817 | 14 | 9.146652e-01 | Pearson | 16 |
| Na | K | L | 0.258549342 | 0.95 | -0.27201913 | 0.6685681 | 1.00145446 | 14 | 3.336030e-01 | Pearson | 16 |
| Mg | K | L | 0.484033432 | 0.95 | -0.01535651 | 0.7901514 | 2.06969692 | 14 | 5.745932e-02 | Pearson | 16 |
| Al | K | L | -0.047329882 | 0.95 | -0.53058676 | 0.4591478 | -0.17729089 | 14 | 8.618193e-01 | Pearson | 16 |
| P | K | L | 0.344882012 | 0.95 | -0.18192523 | 0.7178618 | 1.37477842 | 14 | 1.908052e-01 | Pearson | 16 |
| K | K | L | -0.030992741 | 0.95 | -0.51872870 | 0.4719635 | -0.11601995 | 14 | 9.092845e-01 | Pearson | 16 |
| Ca | K | L | -0.198059460 | 0.95 | -0.63174101 | 0.3300499 | -0.75604795 | 14 | 4.621533e-01 | Pearson | 16 |
| Ti | K | L | 0.463683832 | 0.95 | -0.04157829 | 0.7800860 | 1.95817674 | 14 | 7.044249e-02 | Pearson | 16 |
| V | K | L | -0.086959653 | 0.95 | -0.55858636 | 0.4271589 | -0.32661048 | 14 | 7.487941e-01 | Pearson | 16 |
| Cr | K | L | -0.283170912 | 0.95 | -0.68300350 | 0.2472392 | -1.10474640 | 14 | 2.878949e-01 | Pearson | 16 |
| Mn | K | L | 0.059455908 | 0.95 | -0.44949728 | 0.5392676 | 0.22285789 | 14 | 8.268648e-01 | Pearson | 16 |
| Fe | K | L | 0.271690447 | 0.95 | -0.25888049 | 0.6763113 | 1.05630584 | 14 | 3.087163e-01 | Pearson | 16 |
| Co | K | L | 0.432582586 | 0.95 | -0.08035315 | 0.7643794 | 1.79523740 | 14 | 9.422661e-02 | Pearson | 16 |
| Ni | K | L | 0.047311676 | 0.95 | -0.45916222 | 0.5305736 | 0.17722254 | 14 | 8.618720e-01 | Pearson | 16 |
| Cu | K | L | 0.348232531 | 0.95 | -0.17824087 | 0.7197023 | 1.38996741 | 14 | 1.862452e-01 | Pearson | 16 |
| Zn | K | L | 0.307556919 | 0.95 | -0.22199292 | 0.6969994 | 1.20939224 | 14 | 2.465397e-01 | Pearson | 16 |
| Ga | K | L | 0.048614422 | 0.95 | -0.45813115 | 0.5315112 | 0.18211384 | 14 | 8.581040e-01 | Pearson | 16 |
| Rb | K | L | 0.310147880 | 0.95 | -0.21926817 | 0.6984692 | 1.22065989 | 14 | 2.423759e-01 | Pearson | 16 |
| Sr | K | L | -0.257405334 | 0.95 | -0.66788980 | 0.2731536 | -0.99670812 | 14 | 3.358223e-01 | Pearson | 16 |
| Zr | K | L | 0.647010464 | 0.95 | 0.22274554 | 0.8652176 | 3.17501826 | 14 | 6.747551e-03 | Pearson | 16 |
| As | K | L | 0.130976491 | 0.95 | -0.39005334 | 0.5884747 | 0.49432755 | 14 | 6.287409e-01 | Pearson | 16 |
| Se | K | L | 0.933020590 | 0.95 | 0.81361475 | 0.9769044 | 9.70211338 | 14 | 1.356820e-07 | Pearson | 16 |
| Mo | K | L | 0.195754371 | 0.95 | -0.33218503 | 0.6302978 | 0.74689602 | 14 | 4.674854e-01 | Pearson | 16 |
| Pd | K | L | 0.999615169 | 0.95 | 0.99885905 | 0.9998702 | 134.83063826 | 14 | 3.348030e-23 | Pearson | 16 |
| Ag | K | L | 0.142959826 | 0.95 | -0.37964974 | 0.5964007 | 0.54045801 | 14 | 5.973750e-01 | Pearson | 16 |
| Cd | K | L | 0.769868184 | 0.95 | 0.44336216 | 0.9160018 | 4.51358470 | 14 | 4.863850e-04 | Pearson | 16 |
| Sn | K | L | 0.725575574 | 0.95 | 0.35898810 | 0.8982175 | 3.94519340 | 14 | 1.465536e-03 | Pearson | 16 |
| Sb | K | L | 0.030552983 | 0.95 | -0.47230554 | 0.5184069 | 0.11437219 | 14 | 9.105668e-01 | Pearson | 16 |
| Cs | K | L | 0.672427561 | 0.95 | 0.26508040 | 0.8761045 | 3.39924282 | 14 | 4.319392e-03 | Pearson | 16 |
| Ba | K | L | 0.151358531 | 0.95 | -0.37227857 | 0.6019035 | 0.57293256 | 14 | 5.757774e-01 | Pearson | 16 |
| La | K | L | 0.095129564 | 0.95 | -0.42040026 | 0.5642280 | 0.35756382 | 14 | 7.260028e-01 | Pearson | 16 |
| Ce | K | L | 0.783844375 | 0.95 | 0.47124343 | 0.9214968 | 4.72315406 | 14 | 3.265820e-04 | Pearson | 16 |
| Pr | K | L | 0.229028560 | 0.95 | -0.30083023 | 0.6508432 | 0.88034627 | 14 | 3.935334e-01 | Pearson | 16 |
| Nd | K | L | 0.115917165 | 0.95 | -0.40294149 | 0.5783879 | 0.43666593 | 14 | 6.690130e-01 | Pearson | 16 |
| Sm | K | L | 0.159256368 | 0.95 | -0.36528620 | 0.6070394 | 0.60358618 | 14 | 5.557728e-01 | Pearson | 16 |
| Eu | K | L | 0.252540240 | 0.95 | -0.27796190 | 0.6649975 | 0.97657332 | 14 | 3.453543e-01 | Pearson | 16 |
| Gd | K | L | 0.148345948 | 0.95 | -0.37493020 | 0.5999346 | 0.56126987 | 14 | 5.834867e-01 | Pearson | 16 |
| Tb | K | L | 0.254152494 | 0.95 | -0.27637142 | 0.6659574 | 0.98323697 | 14 | 3.421787e-01 | Pearson | 16 |
| Dy | K | L | 0.362063468 | 0.95 | -0.16287396 | 0.7272452 | 1.45332022 | 14 | 1.681815e-01 | Pearson | 16 |
| Ho | K | L | 0.506709744 | 0.95 | 0.01469565 | 0.8011766 | 2.19916192 | 14 | 4.517120e-02 | Pearson | 16 |
| Er | K | L | 0.065688481 | 0.95 | -0.44449040 | 0.5436901 | 0.24631579 | 14 | 8.090126e-01 | Pearson | 16 |
| Tm | K | L | 0.394957821 | 0.95 | -0.12527399 | 0.7448370 | 1.60857521 | 14 | 1.300201e-01 | Pearson | 16 |
| Yb | K | L | 0.415172079 | 0.95 | -0.10140211 | 0.7554115 | 1.70755021 | 14 | 1.097894e-01 | Pearson | 16 |
| Hf | K | L | 0.350340661 | 0.95 | -0.17591509 | 0.7208577 | 1.39955525 | 14 | 1.834131e-01 | Pearson | 16 |
| Pt | K | L | 0.680189224 | 0.95 | 0.27832906 | 0.8793882 | 3.47190730 | 14 | 3.738224e-03 | Pearson | 16 |
| Hg | K | L | 0.572385704 | 0.95 | 0.10705582 | 0.8320185 | 2.61184346 | 14 | 2.049914e-02 | Pearson | 16 |
| Hg | K | L | 0.572385704 | 0.95 | 0.10705582 | 0.8320185 | 2.61184346 | 14 | 2.049914e-02 | Pearson | 16 |
| Tl | K | L | -0.009009567 | 0.95 | -0.50247082 | 0.4888791 | -0.03371208 | 14 | 9.735827e-01 | Pearson | 16 |
| Pb | K | L | -0.129132889 | 0.95 | -0.58724742 | 0.3916422 | -0.48725063 | 14 | 6.336215e-01 | Pearson | 16 |
| Bi | K | L | 0.367253867 | 0.95 | -0.15704067 | 0.7300532 | 1.47737609 | 14 | 1.617153e-01 | Pearson | 16 |
| Th | K | L | 0.390350491 | 0.95 | -0.13063197 | 0.7424019 | 1.58641383 | 14 | 1.349676e-01 | Pearson | 16 |
| U | K | L | -0.039762522 | 0.95 | -0.52511753 | 0.4651104 | -0.14889549 | 14 | 8.837596e-01 | Pearson | 16 |
